# Epinephrine extensively changes the biofilm matrix composition in *Micrococcus luteus* C01 isolated from human skin

**DOI:** 10.3389/fmicb.2022.1003942

**Published:** 2022-09-20

**Authors:** Andrei V. Gannesen, Rustam H. Ziganshin, Evelina L. Zdorovenko, Alena I. Klimko, Elena A. Ianutsevich, Olga A. Danilova, Vera M. Tereshina, Maxim V. Gorbachevskii, Maria A. Ovcharova, Ekaterina D. Nevolina, Sergey V. Martyanov, Alexander S. Shashkov, Andrey S. Dmitrenok, Andrei A. Novikov, Marina V. Zhurina, Ekaterina A. Botchkova, Philipp V. Toukach, Vladimir K. Plakunov

**Affiliations:** ^1^Winogradsky Institute of Microbiology, Federal Research Center “Fundamentals of Biotechnology” of Russian Academy of Sciences, Moscow, Russia; ^2^Shemyakin and Ovchinnikov Institute of Bioorganic Chemistry, Russian Academy of Sciences, Moscow, Russia; ^3^N.D. Zelinsky Institute of Organic Chemistry, Russian Academy of Sciences, Moscow, Russia; ^4^Faculty of Biology, Lomonosov Moscow State University, Moscow, Russia; ^5^Faculty of Chemical and Environmental Engineering, Gubkin University, Moscow, Russia

**Keywords:** *Micrococcus luteus*, epinephrine, biofilms, biofilm matrix, human skin microbiota, host–microbiota interactions, NMR, mass spectrometry

## Abstract

The importance of the impact of human hormones on commensal microbiota and microbial biofilms is established in lots of studies. In the present investigation, we continued and extended the research of epinephrine effects on the skin commensal *Micrococcus luteus* C01 and its biofilms, and also the matrix changes during the biofilm growth. Epinephrine in concentration 4.9 × 10^–9^ M which is close to normal blood plasma level increased the amount of polysaccharides and extracellular DNA in the matrix, changed extensively its protein, lipid and polysaccharide composition. The Ef-Tu factor was one of the most abundant proteins in the matrix and its amount increased in the presence of the hormone. One of the glucose-mannose polysaccharide was absent in the matrix in presence of epinephrine after 24 h of incubation. The matrix phospholipids were also eradicated by the addition of the hormone. Hence, epinephrine has a great impact on the *M. luteus* biofilms and their matrix composition, and this fact opens wide perspectives for the future research.

## Introduction

Biofilms are considered as a common mode of existence for bacteria and other microorganisms; they are encountered in most natural and artificial environments, including the human body and particularly the skin. The extracellular matrix is an integral part of the biofilm and plays a key role in its growth, metabolism, and maintenance (Singh et al., 2021). Recently, the term “matrixome” was introduced to define the chemical composition of the matrix and highlight its importance for biofilm maintenance (Karygianni et al., 2020). *Micrococcus luteus*, as a regular part of the skin microbiota, can also form biofilms in different microniches of the skin (Lange-Asschenfeldt et al., 2011), where it interacts with other microbes in a commensal community and participates in the maintenance of skin homeostasis (Clabaut et al., 2021; Mart’yanov et al., 2021; Ovcharova et al., 2021; Diuvenji et al., 2022; Kiseleva et al., 2022; Scardaci et al., 2022).

Recently, a great deal of evidence has been obtained regarding the impact of hormones on the commensal bacteria found on the skin. Catecholamines (Borrel et al., 2019), neuropeptides (N’Diaye et al., 2016), steroids (Clabaut et al., 2021; Kiseleva et al., 2022) and natriuretic peptides (Gannesen et al., 2018a) seem to act as effectors on the skin and mucosal microbial community. It has been shown that the effect of some hormones is based on interactions with receptor analogs (as was suggested for C-type natriuretic peptide and *Pseudomonas aeruginosa* by Rosay et al., 2015) or with whole-signal systems (as was described for *Escherichia coli* and catecholamines by Reading et al., 2007). Hence, the effect of human humoral regulators on bacteria seems to be multitargeted and complex, since they can potentially alter bacterial metabolism via different signaling pathways. These results are supported by recent studies showing the dose-dependent effect of natriuretic peptides (Gannesen et al., 2018b; Ovcharova et al., 2021) and catecholamines (Gannesen et al., 2021; Mart’yanov et al., 2021) on the skin commensals *Staphylococcus epidermidis*, *Cutibacterium acnes*, *Staphylococcus aureus*, and *M. luteus*. These effects could potentially alter matrix composition.

Recently, it was found that epinephrine impacts the growth of the biofilm of *M. luteus* C01, isolated from human skin (Danilova et al., 2020; Gannesen et al., 2021), at concentrations close to the physiological norm in human blood plasma (4.9 × 10^–9^ M; Boyanova, 2017). The effect was complex and included shifts in the expression of several genes; it was suggested that alterations in the biofilm matrix were potentially key effects. To prove this, we decided to analyze potential matrix changes using the methods employed in our recent study (Gannesen et al., 2019). The biofilm matrix of *M. luteus* has not been studied in depth. It is known that it consists of mostly exopolysaccharides and proteins (Mauclaire and Egli, 2010), which are common components in the vast majority of bacteria. Thus, the present study aimed first to investigate the composition of the matrix itself and second to determine if the presence of 4.9 × 10^–9^ M epinephrine could change that composition.

## Materials and methods

### Preparation of cultures and biofilm growth

The *M. luteus* C01 strain was isolated from the skin of a healthy volunteer and characterized using 16S rRNA sequencing (Zhurina et al., 2017). It was stored at room temperature in glass tubes with 5 ml of semi-liquid lysogeny broth (LB) with 0.4% agar and covered with sterile mineral oil. For the experiments, a portion of biomass from a tube was plated on a Petri dish with 1.5% agarized reinforced clostridial medium (RCM). The RCM liquid was composed of (in g/L) yeast extract (Dia-M, Russia) 13; peptone (Dia-M, Russia) 10; glucose (Dia-M, Russia) 5; sodium chloride (Dia-M, Russia) 5; sodium acetate (Dia-M, Russia) 3; starch (Dia-M, Russia) 1; cysteine hydrochloride (BD, United States) 0.5; pH 7.0. After incubation at 33°C (the temperature of the skin microniche inhabited by *M. luteus*) for 48 h, a colony was inoculated in 15 ml of liquid LB in a 50 ml conical flask and cultivated overnight at 33°C.

For biofilm growth, 20 ml of RCM with 1.5% agar and 4.9 × 10^–9^ M (4.9 nM) epinephrine (physiological blood plasma concentration; Boyanova, 2017) were added to Petri dishes; controls without the hormone were also prepared. A 90 mm circle cellulose filter was placed onto the agar surface, and 0.5 ml of the overnight culture with an OD_540_ value adjusted to 0.5 was inoculated onto the filter and spread with a sterile glass spatula. Afterward, the biofilms were grown for 24 h or 72 h to obtain young and mature biofilms, respectively. After incubation, the biomass was collected into a 50 ml conical centrifuge tube (Falcon, United States) and the wet biomass was weighed to adjust the for the matrix amounts in subsequent experiments.

### Matrix isolation

After weighing, the biofilm biomass was transferred into 50 ml conical tubes (Corning, United States) and resuspended in distilled water. Then, the samples were placed into an ice bath and sonicated for 10 min with a Sonyprep 105 Plus (MSE, GB) using the appropriate titanium horn (rod or exponential, depending on the sample volume) at an amplitude of 7.3 or 10.1 μm, respectively. After sonication, the samples were transferred into polypropylene ultracentrifuge tubes (Beckman, United States) and layered onto the CsCl gradient solution. The CsCl gradient was prepared as described previously (Gannesen et al., 2019). Briefly, four CsCl solutions (46%, 28%, 14%, and 7%) were layered, and the sample was added to the top of the tube. The samples were centrifuged in an Avanti-J30I (Beckman, United States) ultracentrifuge at 100,000 × *g* and RT for 1 h. After, to avoid the contamination of the matrix with cells, the supernatant was transferred into new conical tubes, and any cells carried over during the supernatant transfer were additionally removed by centrifugation at 3,800 × *g* and RT for 20 min. Before analysis, the matrix samples were dialyzed in bags with a pore size of 0.1- to 0.5 kDa (Spectrum Repligen, United States) to remove CsCl.

### Complementary biofilm extraction test

To ensure that the matrix isolation was successful and that no cells were destroyed and lysed during the isolation process, an LDH test was performed using a standard LDH assay kit (Vital, Russia). This assay was chosen because LDH is a mostly intracellular enzyme that is also found in *M. luteus* (the data were deposited with links to BioProject accession number PRJNA564862 in the NCBI BioProject database^1^). The LDH reaction used in this study can be quantified based on a decrease in the OD_340_ of the samples in the presence of the substrate. A greater decrease indicates a more active reaction, and thus more LDH in the sample, which denotes a higher level of cell disruption. The cell suspensions were adjusted to have the same OD_340_ value as the matrix, and a standard protocol was used to compare the resulting OD values in a 96-well plate.

### Quantification of total organic carbon in the *Micrococcus luteus* C01 biofilm matrix

The total organic carbon content of the matrix was measured using the wet mineralization method based on the classic method described previously (Walkley, 1947; Gannesen et al., 2019). Briefly, 14.71 g of potassium dichromate (Reachim, Russia) was dissolved in 150 ml of MQ water and 100 ml of 96% sulfuric acid (SA, Reachim, Russia) was added. The matrix samples were diluted 10 times, and 1 ml of each diluted sample was mixed with 3 ml of reagent mix and incubated in a boiling water bath (100°C). In parallel, a series of glucose solutions of various concentrations (10–2,000 μg/ml in MQ water) were prepared to make calibration curves. After boiling for 90 min, the samples were cooled and their OD_590_ values were measured. The total organic carbon content was quantified on the basis of a carbon mass proportion of 40% in glucose (the molecular mass of glucose is 180 g/mol, of which carbon is 72 g/mol).

### Quantification of reducing sugars and proteins in the matrix

The concentration of reducing sugars, as a marker of total carbohydrates, was measured using the anthrone method, as described previously (Dreywood, 1946; Gannesen et al., 2019). Briefly, 0.2 g of anthrone (Sigma, United States) was dissolved in 100 ml of 96% SA (Reachim, Russia) to prepare the anthrone reagent. Standard glucose solutions were prepared for calibration curves. One gram of glucose (Dia-M, Russia) was dissolved in 100 ml of a benzoic acid solution made by dissolving 2.5 g of benzoic acid in 1 L of MQ water. The standard glucose solutions were stored at 4°C in the dark. The matrix samples were diluted 10-fold in MQ water. A double volume of the reagent was added to each sample, and the samples were mixed and heated at 100°C for 16 min. In parallel, the same reaction was performed using a series of glucose solutions (made from a standard solution) with concentrations ranging from 5 to 50 μg/ml. MQ water was used as a negative control. After incubation, the tubes were cooled to RT and the OD_625_ values were estimated. Finally, the concentration of reducing sugars was calculated using the calibration curve.

Proteins were quantified using the Bradford method as described in Bradford (1976). Standard Bradford reagent (Sigma) and bovine serum albumin (BSA, Sigma) were used.

### Detection of DNA concentration in the matrix

The DNA concentration in the matrix was estimated using the Dische method (Dische, 1930). This method is based on the reaction of desoxyribose with diphenylamine in acetic acid. When heated, levulinic aldehyde is produced, which then condenses with diphenylamine to form a colored compound. Briefly, the diphenylamine solution was prepared by dissolving 1 g of diphenylamine (Sigma) in 100 ml of pure acetic acid (Sigma, United States). Then, 2.75 ml of 96% SA was added. A calibration series of standard DNA solutions (50–500 μg/ml) was then prepared using herring sperm DNA (Sigma), and the matrix samples were prepared for analysis. All samples (2 ml) were distributed into glass tubes. Then, a double amount of Dische reagent (4 ml) was added, and the samples were heated for 15 min at 100°C in a water bath. The OD595 values were measured after cooling. MQ water was used as a control.

### Extraction of lipids from the biofilm cells and matrix

A sample of wet biomass was homogenized in isopropanol (Rushim, Russia) and incubated at 70°C for 30 min (Nichols, 1963). After decanting the supernatant, the pellet was extracted twice at 70°C with an isopropanol–chloroform mixture (1:1, and once with 1:2). The total final extract was evaporated in a rotary evaporator, and the residue was dissolved in 9 ml of chloroform–methanol (Rushim, Russia) mixture (1:1) with the addition of 12 ml 2.5% NaCl for the removal of water-soluble compounds. After phase separation, the chloroform layer was dried by passing through water-free sodium sulfate, evaporated, and desiccated with a vacuum pump. The residue was dissolved in a chloroform–methanol mixture (2:1) and stored at −21°C.

To extract lipids from the matrix, 20 ml of matrix suspension was mixed with 20 ml of the chloroform–methanol mixture (2:1). The mixture was vortexed for 30 s and left for 30 min at room temperature for phase separation. The lower chloroform phase was isolated after the aqueous phase was extracted twice by the addition of 10 ml of chloroform in the same conditions. Fifty milliliters of 2.5% NaCl was added to the total extract, and the mixture was kept overnight at 4°C for the removal of water-soluble compounds and phase separation. After, the chloroform layer was dried by passing through water-free sodium sulfate, evaporated, and desiccated with a vacuum pump. The residue was dissolved in the chloroform–methanol mixture (2:1) and stored at −21°C.

The separation of polar lipids was conducted using two-dimensional thin-layer chromatography on silica gel 60 glass plates (Merck) in the following systems: chloroform–methanol–water (65:25:4) in the first direction and chloroform–acetone–methanol–acetic acid–water (50:20:10:10:5) in the second direction (Benning et al., 1995). A total of 200–250 μg of lipids was applied to a plate. Neutral lipids were analyzed using one-dimensional thin-layer ascending chromatography on 10 × 10 cm silica gel 60 glass plates (Merck, Germany). For lipid separation, a hexane–diethyl ether–acetic acid (77:23:1) solvent system was used. A total of 125 μg of lipids was applied to a glass plate.

To determine the general composition of polar and neutral lipids, components were visualized by spraying the plates with 5% H_2_SO_4_ (Rushim) in ethanol (v/v) followed by heating for 15 min at 180°C. For the identification of polar lipids, individual standard compounds were used and reactions with molybdene blue dye were conducted according to the modified Dittmer and Lester method (Vaskovsky et al., 1975) for phospholipids. Additionally, a reaction with ninhydrin was performed to identify aminolipids, an α-naphthol test was used for glycolipids, and Dragendorff’s reagent was used to detect choline (Kates, 1972). The individual phospholipid standards used were phosphatidylethanolamines, phosphatidylcholines, phosphatidylglycerols, and diphosphatidylglycerols (Larodan, Sweden). Neutral lipids were identified using individual markers for sterols; mono-, di-, and triacylglycerols; and free fatty acids (Sigma, United States).

### Orbitrap mass spectrometry investigation of the *Micrococcus luteus* C01 matrix proteome

Protein samples from the matrix were prepared for Orbitrap mass spectrometry (MS). First, the matrix samples were dialyzed in bags with a pore size of 0.1- to 0.5 kDa (Spectrum Repligen, United States) to remove CsCl. Then, the samples were mixed with acetone (4 × volume) and incubated for 10 min at RT to precipitate the proteins. After precipitation, the samples were centrifuged at RT and 15,000 × *g* for 30 min to collect the proteins. The liquid supernatant was removed, and the samples were transferred into sterile microcentrifuge tubes (Eppendorf, Germany) and air-dried at room temperature. For the MS experiments, the samples were loaded to a homemade trap column (20 × 0.1 mm) packed with Inertsil ODS3 3 m sorbent (GL Sciences, Japan) in loading buffer (2% acetonitrile, 98% H_2_O, and 0.1% trifluoroacetic acid) at 10 μl/min flow and separated at RT in a homemade (Kovalchuk et al., 2019) fused-silica column (300 × 0.1 mm) packed with Reprosil PUR C18AQ 1.9 (Dr. Maisch, Germany) into an emitter prepared with a P2000 Laser Puller (Sutter, United States). Reverse-phase chromatography was performed using an Ultimate 3000 Nano LC System (ThermoFisher, United States), which was coupled to a Q Exactive Plus Orbitrap mass spectrometer (ThermoFisher, United States) via a nanoelectrospray source (ThermoFisher, United States). The peptides were loaded in a loading solution [98% 0.1% (v/v) formic acid and 2% (v/v) acetonitrile] and eluted with a linear gradient as follows: 3–35% solution B [0.1% (v/v) formic acid and 80% (v/v) acetonitrile] for 105 min; 35–55% B for 18 min; 55–99% B for 0.1 min; 99% B for 10 min; and 99–2% B for 0.1 min at a flow rate of 500 nl/min. After each gradient, the column was re-equilibrated with solution A [0.1% (v/v) formic acid and 2% (v/v) acetonitrile] for 10 min. The MS1 parameters were as follows: resolution of 70K, scan range of 350–2000, max injection time of 30 ms, and AGC target of 3 × 10^6^. Ions were isolated with 1.4 m/z window via preferred peptide match and isotope exclusion. Dynamic exclusion was set to 30 s. MS2 fragmentation was carried out in HCD mode at a resolution of 17,5K, HCD collision energy of 29%, max injection time of 80 ms, and AGC target of 1 × 10^5^. The charge exclusion was unassigned (1, >7).

Raw spectra were processed using the MaxQuant 1.6.6.0 (MQ) (Tyanova et al., 2016a) and Perseus (Tyanova et al., 2016b) software. The data were searched against the *Micrococcus luteus* UniProt Tremble database, which contains canonical and isoform proteins (version from 15.03.2021).

A *MaxQuant* search was performed using the default parameter settings, including trypsin/p protease specificity, maximum two missed cleavages, Met oxidation, protein N-term acetylation, and NQ deamidation, as variable modifications and carbamidomethyl Cys as a fixed modification, with a maximum of five modifications per peptide, 1% PSM, and protein FDR. The following options were selected: second peptide, maxLFQ, and match between runs. All runs were analyzed as independent experiments and processed in Perseus (Tyanova et al., 2016a,b).

Using the Perseus software, protein groups were filtered for contaminants, i.e., reverse and “identified only by site” proteins. Only the proteins with maxLFQ values in at least three out of seven LC-MS runs were used. The missing values were imputed from a normal distribution with an intensity distribution sigma width of 0.3 and an intensity distribution center downshift of 1.8. A two-sample *t*-test with a permutation-based FDR of 5% was applied to search for significantly changed proteins.

A gene ontology analysis was also performed using the UniProt online resources.

Data are available via ProteomeXchange with identifier PXD035739.

### Surface-enhanced Raman spectroscopy of the *Micrococcus luteus* C01 matrix

Surface-enhanced Raman spectroscopy is a powerful method for the non-destructive study of organic samples, particularly bacterial biofilms (Chao and Zhang, 2012). With suitable active substrates and near-infrared excitation, it is possible to register the Raman signals of characteristic bacterial substances [nicotinamide adenine dinucleotide (NAD)-like redox complexes, proteins, etc.] (Efrima and Zeiri, 2009) and discriminate between bacteria, at least at the genus level (Kopitsyn et al., 2019). Surface-enhanced Raman scattering (SERS) substrates were prepared as described elsewhere (Gorbachevskii et al., 2018; Kopitsyn et al., 2019).

Surface-enhanced Raman scattering was used in this study to obtain the spectra of *M. luteus* C01 biofilms and verify successful matrix extraction. The intact biomass of the biofilms, cell biomass after matrix extraction, and isolated biofilm matrix of *M. luteus* C01 were used for SERS analysis. The biomass (intact and after matrix extraction) of *M. luteus* C01 was washed three times with acetone and dried at RT. Before SERS analysis, dry samples were washed twice with a 0.9% NaCl solution. The pellets were resuspended, centrifuged at 3,000 × *g*, and kept at RT for 10 min before analysis.

Nanoparticles were characterized using transmission electron microscopy (TEM) and scanning electron microscopy (SEM). For TEM, 5 μL of a nanoparticle suspension was placed on a TEM grid (Ted Pella, Redding, CA, United States) and dried at RT. TEM images were obtained using a JEM-2100 microscope (Jeol) with an acceleration voltage of 200 kV. SEM images were obtained using a JIB-4501 microscope (Jeol).

Surface-enhanced Raman scattering spectra were registered using a BWS415 spectrophotometer (BWTEK). Samples were placed on an XYZ stage, and the laser position was controlled via a USB-connected Mikmed-2000R microscope (Micromed). Various substrates based on gold nanomaterials were tested to register the SERS signals.

### Isolation of matrix polysaccharides

The matrix of *M. luteus* C01 was dialyzed against distilled water in small-pore bags. After dialysis, an aliquot of trichloroacetic acid was added to the matrix sample to establish a final pH of 2.0 for nucleic acid and protein sedimentation.

The polysaccharide preparations were fractionated using anion-exchange chromatography on a column (20 × 1 cm) of DEAE-Trisacryl M with a stepwise gradient of 0.005, 0.1, and 0.5 M hydrogen sodium phosphate, pH 6.3. As result, two fractions were obtained: one eluted in 0.5 M buffer and the other eluted in 0.1 M buffer.

### Chemical methods

Hydrolysis was performed with 2 M CF_3_CO_2_H (120°C, 2 h). The monosaccharides were analyzed using GLC as alditol acetates (Sawardeker et al., 1965) on a Maestro 7820 GC instrument (Interlab, Russia) equipped with an HP-5 ms column with the following temperature program: 160°C (1 min) to 290°C at 7°C per min. The absolute configurations of the monosaccharides were determined via GLC of the acetylated (*S*)-2-octyl glycosides as described previously (Leontein and Lönngren, 1993).

### Nuclear magnetic resonance spectroscopy

Nuclear magnetic resonance (NMR) spectra were recorded for the solutions in 99.9% D_2_O after deuterium exchange by freeze-drying from 99.9% D_2_O. A ROESY experiment was also carried out in a 9:1 D_2_O/H_2_O mixture. Spectra were measured with Bruker DRX-500 and Bruker Avance II 600 spectrometers with a 5 mm broadband inverse probehead at 30°C. Sodium 3-(trimethylsilyl)propanoate-2,2,3,3-d4 (δ_*H*_ 0.0, δ_*C*_ -1.6) was used as an internal standard for chemical shift reference. Two-dimensional NMR experiments were performed using the standard Bruker pulse programs. The 2D TOCSY and ROESY spectra were recorded with a 60 ms duration of MLEV 17 spinlock and a 150 ms mixing time. The gradient-selected ^1^H,^13^C HMBC spectrum was recorded with a 60 ms delay for the evolution of long-range spin couplings.

### Smith degradation

A PS sample (14 mg) was oxidized with 0.1 M NaIO_4_ in the dark at 20°C for 48 h. After the addition of an excess of ethylene glycol, the products were reduced with NaBH_4_, desalted with GPC on a column (80 × 1.6 cm) of TSK HW-40 (S) in water monitored using a differential refractometer (Knauer, Germany), and hydrolyzed with aq 2% AcOH at 100°C for 2 h. The products were fractionated using GPC on a column of TSK HW-40 (S) as above to produce PS III (2 mg).

The ^13^C NMR chemical shifts were analyzed and compared to published values using GODDESS (Kapaev and Toukach, 2016) and GRASS NMR (Kapaev and Toukach, 2018) simulation, and structure elucidation web services from the Carbohydrate Structure Database platform were also implemented (Toukach and Egorova, 2016).

### Statistics

All the experiments were conducted at least in triplicate. In the tables, where applicable, an average value is presented with standard deviations. A *t*-test was used for statistical evaluation.

## Results

### Biofilm matrix isolation and complementary extraction test

**FIGURE 1:**
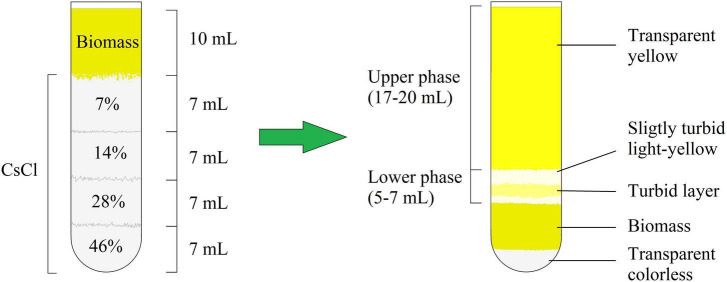
CsCl gradient formation and biofilm matrix following extraction after 72 h of incubation.

After centrifugation, the *M. luteus* C01 biofilm matrix solution consisted of two parts, which were conditionally labeled as the “upper phase” and “lower phase” (Figure 1 and Supplementary Figure 1). Generally, the visual appearance of the matrix was similar to that observed in a previous study (Gannesen et al., 2019). Figure 1 depicts a scheme of the mature 72 h biofilms. In case of the 24 h immature biofilms, the scheme was the same with slight changes in the proportions of biomass added (less volume of biomass and the lower phase). In addition, in the 24 h supernatants, the lower phase was more colored (Supplementary Figure 1B). The additional centrifugation of the lower phase allowed for the decantation of the turbid suspension; thus, this observation could be due a fraction of smaller cell aggregates or single cells that were unable to pass through the dense CsCl solution due to their relatively low mass.

**FIGURE 2:**
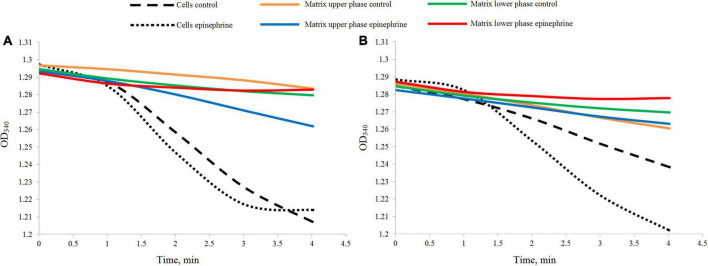
Differences in the dynamics of the LDH reaction measured in the biofilm cell biomass and matrix samples. **(A)** Samples after 24 h of incubation. **(B)** Samples after 72 h of incubation.

The LDH test demonstrated a rather weak reaction in the samples (Figure 2). Nevertheless, the differences between the cell suspensions and matrix samples were well established. After 24 h of incubation, the cell biomass samples, both the control and in the presence of epinephrine, showed a higher rate of the reaction (Figure 2A), while the matrix samples were inactive or had a much slower rate. After 72 h of incubation (Figure 2B), the reaction results were same, except the control cell biomass exhibited a slower rate than in the 24 h samples. This potentially could be explained by the metabolic activity ratio of the biofilm cells. Since the matrix samples had a reaction rate different from that of the cell biomass samples, it was established that the pure matrix fraction was obtained in all our experiments.

Additionally, we carried out a microscopy study of cell disruption after sonication and matrix isolation (Supplementary Figure 2). We found that after sonication, there was no visible cell disruption, and even cell aggregates remained intact (Supplementary Figure 2B); however, there were more single cells that were stained with less intensity (Supplementary Figure 2C). This could be a result of partial matrix removal from the cell aggregates’ surface. After centrifugation, *M. luteus* C01 cells were also found in a turbid layer above the cell biomass (Supplementary Figure 2D).

### General biochemical composition of the *Micrococcus luteus* C01 matrix

The results of the quantitative biochemical tests (DNA, proteins, sugars, total organic carbon) demonstrated that the matrix consists of all the main polymer types (Table 1). The most abundant component was a polysaccharide compartment of the matrix, the second were proteins and finally DNA. All the components varied depending on the matrix phase, incubation time and the presence of epinephrine. Interesting that epinephrine increased the absolute amount of total organic carbon (TOC) and lower molecular weight sugars in mature biofilm. Despite that, the percentage of those compounds did not change probably due to the high amount of other organics (especially pigments), because of less than a half of a total organic mass of the matrix were sugars, DNA and proteins. Nevertheless, the previous hypothesis of matrix synthesis stimulation (Gannesen et al., 2021) was approved. If calculate approximately, the amount of dry matrix in experiments oscillated from 20 to 50 mg after the dialysis.

**TABLE 1:** Biochemical composition of *M. luteus* C01 matrix in the presence of epinephrine and in control samples.

Absolute meanings, μg/g		Sugars		Proteins		DNA		TOC	
					
		24 h	72 h	24 h	72 h	24 h	72 h	24 h	72 h
Upper phase	Control	1380.9 ± 207.6	1401.8 ± 242.4	337.3 ± 33.9	196 ± 30.1	122.2 ± 40.6	107.4 ± 22	1945 ± 678	2340.5 ± 221.5
	Epinephrine	1446.7 ± 316.3	1692.1 ± 219.5	319 ± 32	224.2 ± 37.3	104.6 ± 23.6	137 ± 22.2	2121.1 ± 620	3174.8 ± 384.3
Lower phase	Control	381.8 ± 67	410.2 ± 85.8	70.6 ± 11.8	49.9 ± 10.2	20.9 ± 3.3	17.5 ± 0.8	325.6 ± 58.2	563.4 ± 75.2
	Epinephrine	440 ± 141	406.8 ± 133.8	80.4 ± 11.7	52.2 ± 14.6	27.9 ± 7	12.7 ± 0.8	517.7 ± 162	530.1 ± 198.6
Total matrix	Control	1762.6 ± 254	1811.9 ± 314.5	407.9 ± 26	245.9 ± 30.3	143.1 ± 42.2	125 ± 21.5	2270.6 ± 693.7	2903.9 ± 233.2
	Epinephrine	1887.1 ± 407	2180.7 ± 343.6	399.4 ± 26	276.4 ± 44.7	132.4 ± 27.7	149.7 ± 22.8	2638.8 ± 768.8	3704.9 ± 384.3
**Percent**									
Upper phase	Control	28.4 ± 4.3	23.9 ± 4.1	7.8 ± 0.8	3.8 ± 0.6	2 ± 0.7	1.5 ± 0.3	100 ± 34.8	100 ± 9.5
	Epinephrine	27.3 ± 6	21.3 ± 2.8	6.8 ± 0.7	3.2 ± 0.5	1.7 ± 0.4	1.4 ± 0.2	109.1 ± 31.9	135.6 ± 16.4
Lower phase	Control	46.9 ± 8.2	29.1 ± 6.1	9.8 ± 1.6	4 ± 0.8	2.1 ± 0.3	1 ± 0.05	100 ± 17.9	100 ± 13.3
	Epinephrine	34 ± 11	30.7 ± 10.1	7 ± 1	4.4 ± 1.2	1.6 ± 0.3	0.8 ± 0.05	159 ± 49.7	94.1 ± 35.2
Total matrix	Control	31.1 ± 4.5	24.9 ± 4.3	8.1 ± 0.5	3.8 ± 0.5	2 ± 0.6	1.4 ± 0.2	100 ± 30.5	100 ± 8
	Epinephrine	28.6 ± 6.1	23.5 ± 3.7	7.3 ± 2	3.3 ± 0.5	1.6 ± 0.3	1.3 ± 0.2	116.2 ± 33.9	127.6 ± 13.9

Standard deviations are marked as variations of parameters. Green indicates significant differences (t-test for paired samples, p < 0.05).

### Proteomic analysis of *Micrococcus luteus* C01 biofilm matrix

In total, 727 proteins were identified in the biofilm matrix of *M. luteus* C01 under the different conditions. Four dominating groups of proteins were identified according to the gene ontology analysis: catalytic, binding, cell anatomy, and metabolic compartment. Hence, the addition of epinephrine had an impact mostly on these protein groups. Catalytic compartment proteins include those with oxidoreductase, transferase, and hydrolase activities. In this study, ion-binding proteins were the largest group in the catalytic compartment. Membrane proteins were considerably more well-represented than cytoplasm proteins (277 and 59, respectively), and proteins involved in different metabolic pathways were the least represented group. Interestingly, that alpha and beta subunits of ATP synthase, the UPF0182 protein (predicted protein export machinery, Vergara et al., 2020), and succinate dehydrogenase were the most abundant proteins in the matrix (Supplementary Table 1). The elongation factor EF-Tu was one of the most abundant proteins (Supplementary Table 1), and it is worth noting that its concentration increased significantly during the incubation with epinephrine (Supplementary Table 2). A comparison of protein lists in different conditions showed that the increase in EF-Tu was caused by the addition of epinephrine and was not associated with biofilm maturation. In the total proteome, 94 proteins—frequently associated with membranes—were uncharacterized. The maturation of *M. luteus* C01 biofilms led to an increase in the concentration of the chaperone DnaK in the matrix; this increase was not affected by epinephrine (Supplementary Table 3). DnaK was a member of the top 100 matrix proteins of *M. luteus*. To the contrary, the concentration of the chaperone DnaJ increased in presence of epinephrine, and the same trend was observed for the chaperone PqqD, chaperonin 10 kDa, chaperonin 60 kDa, and copper chaperones. However, the last four proteins were also stimulated during maturation without epinephrine administration; thus, they were potentially not affected by the hormone administration. Peptidoglycan-binding proteins were also among the most common proteins in the matrix (Supplementary Table 1).

After 24 of incubation in the presence of epinephrine, the concentrations of 53 proteins were altered (Table 2), where 22 were increased and 31 were decreased. In mature biofilms, the administration of epinephrine led to changes in the concentrations of only in eight proteins (Table 3). Many proteins are found in the membranes and cytoplasm of cells, and their presence in a matrix can be a result of cell autolysis. Hence, we analyzed both the matrix and—at least partially—cell proteomes. We concluded that (i) the impact of epinephrine was greater in the initial stages of biofilm growth and (ii) epinephrine potentially triggered some signal processes that led to significant changes both in the cellular proteome and in the matrix proteome. Despite the insignificant alterations in total protein amounts measured using the Bradford test, the changes in proteomes potentially led to adjustments in the matrix structure (i.e., DNA-binding proteins, peptidoglycan-binding proteins, chaperones). This could explain why the amount of matrix increased when measured using dichromate combustion. Interestingly, in presence of epinephrine in 72 h mature biofilms, there was a decrease in histidine kinase concentration (accession A0A5E8QFN5). Histidine kinase is an intracellular enzyme that is potentially involved in signaling. Thus, a decrease in the amount of histidine kinase after the autolysis of cells could potentially be a result of changes in signaling pathways and hence in matrix composition.

**TABLE 2:** Changes in *M. luteus* C01 biofilm matrix protein concentrations in the presence of 4.9 × 10^–9^ M epinephrine after 24 h of incubation in comparison with control.

No	Accession	Protein name	Fold change	Mol. weight (kDa)	MS/MS count
1	A0A653LU13	Inositol-1-monophosphatase	6.61	31.378	14
2	D3LLF7	Glutaredoxin domain-containing protein	5.85	18.144	6
3	A0A410XR61	Type 1 glutamine amidotransferase domain-containing protein	3.04	24.132	35
4	A0A6N4C5 × 5	Peptidase S8	2.92	64.195	433
5	D3LSF8	Uncharacterized protein	2.70	20.15	35
6	A0A5F0I8I8	Preprotein translocase subunit YajC	2.64	15.163	86
7	A0A5E8QCZ2	LysM peptidoglycan-binding domain-containing protein	2.60	71.408	33
8	A0A1M7BB19	Probable cytosol aminopeptidase	2.50	54.491	36
9	A0A509Y3L1	MBL fold metallohydrolase	2.49	24.133	20
10	A0A562FT59	Phage shock protein A (PspA) family protein	2.11	29.31	81
11	A0A4Y8PIL2	Non-specific serine/threonine protein kinase	2.03	71.123	62
12	A0A031HLY3	Amidohydrolase, imidazolonepropionase	2.03	43.201	86
13	A0A4Y8PLE4	SSD domain-containing protein	2.00	113.59	293
14	A0A5F0I5R8	Phosphate-binding protein PstS	1.89	38.326	280
15	D3LNP1	ATP-dependent Clp protease proteolytic subunit	1.89	24.332	8
16	C5CBL5	Multisubunit Na+/H+ antiporter, MnhC subunit	1.87	15.009	29
17	A0A5E8QG41	Peptidase	1.83	101.19	374
18	A0A2N6RSH2	NDP-hexose 4-ketoreductase	1.81	93.374	137
19	C5CBP6	Triosephosphate isomerase	1.79	27.48	40
20	A0A031I4R0	ABC transporter substrate-binding protein	1.68	38.056	39
21	A0A5E8QFG8	Uncharacterized protein	1.65	32.171	351
22	A0A5F0I5P1	ABC transporter substrate-binding protein	1.48	60.578	412
23	A0A4Y8ZII3	MFS transporter (fragment)	0.69	51.295	35
24	A0A2I1XY38	Uncharacterized protein	0.61	53.742	183
25	A0A031IU02	Acyltransferase	0.59	26.765	13
26	A0A5E8QD19	1-Deoxy-D-xylulose-5-phosphate synthase	0.55	71.346	23
27	C5CCE6	Coenzyme A biosynthesis bifunctional protein CoaBC	0.54	43.718	9
28	A0A1R4J0T4	Cysteine synthase	0.54	32.51	23
29	A0A031H6Z1	DNA/RNA helicase, superfamily II	0.51	65.708	71
30	A0A410XNH4	ABC transporter ATP-binding protein	0.49	65.461	42
31	A0A562FPF4	1-Acyl-sn-glycerol-3-phosphate acyltransferase	0.48	25.501	39
32	A0A031GTE4	Uncharacterized protein	0.46	62.278	44
33	A0A132HXJ4	Ribonucleoside-diphosphate reductase subunit beta	0.44	37.516	109
34	A0A653IY01	Metallophosphoesterase	0.43	33.418	45
35	A0A5F0IBK0	Glycine dehydrogenase (decarboxylating)	0.41	104.08	179
36	D3LKU8	Nucleoside diphosphate kinase	0.39	15.291	73
37	P33102	50S ribosomal protein	0.37	12.961	27
38	A0A031G7E5	Thymidine phosphorylase	0.37	46.184	10
39	A0A509Y330	Citrate synthase	0.36	48.148	54
40	A0A5F0I6C5	M13 family peptidase	0.36	75.904	46
41	A0A5E8QBT4	Hydrolase	0.36	44.192	5
42	D3LKR4	Signal recognition particle protein	0.33	56.531	47
43	A0A378NPK0	Spectinomycin tetracycline efflux pump	0.32	51.017	11
44	A0A031HS73	Alanine aminopeptidase	0.30	96.24	78
45	A0A031HT42	Isoleucine–tRNA ligase	0.27	125.71	44
46	A0A5E8QBB1	Cytokinin riboside 5-monophosphate phosphoribohydrolase	0.25	28.048	12
47	A0A2I1XS51	IMP dehydrogenase	0.23	37.348	31
48	A0A1M7AJU5	Methylmalonate-semialdehyde dehydrogenase [acylating]	0.21	53.7	140
49	A0A378NUP4	Beta-(1–>2)glucan export ATP-binding/permease protein NdvA	0.20	9.0263	12
50	A0A1R4IF19	2,3-Butanediol dehydrogenase, R-alcohol forming, (R)-and (S)-acetoin-specific	0.17	37.876	34
51	A0A5F0IBI2	Uncharacterized protein	0.16	31.627	13
52	D3LPH3	SNARE-like domain protein	0.14	23.382	6
53	A0A653IZW6	Copper (Cu(I)) transporter ATPase	0.09	84.519	8

Light green—increase in concentration; dark pink—decrease in concentration.

**TABLE 3:** Changes in *M. luteus* C01 biofilm matrix protein concentrations in the presence of 4.9 × 10^–9^ M epinephrine after 72 h of incubation in comparison with control.

Accession	Protein name	Fold change	Mol. weight (kDa)	MS/MS count
A0A653QWC7	DNA protection during starvation protein	25.51	22.266	10
A0A1M7AJU5	Methylmalonate-semialdehyde dehydrogenase (acylating)	20.11	53.7	140
A0A4U1LCZ2	Agmatinase	19.87	35.919	36
A0A5F0I6E2	Phage holin family protein	15.57	16.423	40
P33102	50S ribosomal protein L18	11.89	12.961	27
A0A031IM63	S-adenosylmethionine synthase	10.50	44.787	108
D3LNP8	Hydrolase, alpha/beta domain protein	9.93	30.406	22
A0A378NH58	Manganese transport protein MntH	3.87	48.117	49
A0A031G1Y7	Uncharacterized protein	3.65	14.661	66
A0A378NNE4	Daunorubicin/doxorubicin resistance ATP-binding protein DrrA	3.38	29.108	127
A0A5F0IBV8	Sodium:proton antiporter	0.53	11.837	44
A0A5E8QFN5	Histidine kinase	0.45	64.426	51

Light green—increased concentration due to biofilm maturation; dark green—increased concentration in the presence of epinephrine; dark orange—downregulation caused by epinephrine.

### Lipids in the *Micrococcus luteus* C01 biofilm cells and matrix

The lipids found in the matrix were previously shown to be normal components of the cellular membranes and excretomes of *M. luteus* (Peterson et al., 1975; de Bony et al., 1989; Beuling et al., 1996; Pakkiri et al., 2004). The data are presented in Table 4 and in the Supplementary Figures 3, 4.

**TABLE 4:** Lipids detected in *M. luteus* C01 biofilm matrix.

Component	Control 24 h		Control 72 h		Epinephrine 24 h		Epinephrine 72 h	
				
	Abs	%	Abs	%	Abs	%	Abs	%
Diphosphatidylglycerol	1.75	0.05	0	0	0	0	0	0
Phosphatidylglycerol	11.58	0.33	0	0	0	0	0	0
Unidentified phospholipid	4.77	?	0	0	0	0	0	0
Unidentified polar lipid 1	15.65	?	0	0	0	0	0	0
Unidentified polar lipid 2	14.11	?	3.86	?	0	0	4.4	?
Unidentified glycolipid	39.77	?	8.82	?	5.51	?	14.61	?
Sterols	12.05	0.44	3.76	0.11	4.12	0.13	7.26	0.16
Sterol esters	11.15	0.41	4.82	0.14	2.58	0.08	12.91	0.29
Diacylglycerols	16.48	0.55	7.59	0.2	6.47	0.19	12.01	0.25
Triacylglycerols	12.14	0.48	6.17	0.19	4.84	0.17	4.75	0.119
Free fatty acids	36.41	1.22	10.55	0.28	17.37	0.5	15.54	0.327
Monoacylglycerols	33.24	1.04	8.1	0.2	14.47	0.39	20.84	0.4
Total	209.14	4.52 (?)	53.68	1.11 (?)	55.38	1.452 (?)	92.33	1.53 (?)

The membrane lipids of biofilm cells were mainly represented by a number of glycolipids, sterols, DPG, PG, and PC (Supplementary Figures 3, 4). The storage lipids included mono-, di-, and triacylglycerols; FFA; and SE. The main lipid components of the biofilm matrix in all variants were glycolipids; sterols; mono-, di-, and triacylglycerols; FFA; and SE (Supplementary Figures 3, 4).

In terms of the matrix, the 24 h control samples contained more lipids than the rest of the samples (Table 4). Diphosphatidylglycerols and phosphatidylglycerols were found only in the 24 h control sample, in addition to the two unidentified phospholipids. The total amount of lipids was about 210 μg/g of the wet biomass, which was 4.5% of the total organics. In presence of epinephrine and after 24 h of incubation, the amount of lipids was decreased by four times. In the 72 h epinephrine samples, the amount of lipids was increased in comparison to the 72 h control; however, it was still lower than in the immature biofilms grown on medium without epinephrine additions. Changes occurred in all lipid classes. Interestingly, phospholipids were present only in the 24 h control samples. After biofilm maturation and after epinephrine addition in the 24 h samples, phospholipids were absent.

Thus, epinephrine caused changes in the concentrations of lipids in the matrix; the observed decrease in immature and increase in mature biofilms suggests that the differences lie in the basis of these processes. In addition, the hormone affected the synthesis of phospholipids. We were unable to calculate the amounts of some lipids due to the unknown nature of the compounds. Despite this, we concluded that the total amount of lipids in the 24 h control samples was no more than 7.5% of the total organic matter in the biofilm matrix. In other samples, it was determined to be no more than 2%; thus, the lipid fraction was a minor fraction of the organic compounds in the matrix.

Thus, epinephrine caused changes in all classes of organic compounds. We concluded that the general composition of the *M. luteus* matrix was (in order of amount): polysaccharides, proteins, lipids, and DNA. These compounds comprised about a half of all the organic material in matrix. The rest were pigments [potentially sarcinaxantin (Netzer et al., 2010)] and different metabolites with low molecular weights.

### Surface-enhanced Raman scattering spectroscopy of *Micrococcus luteus* C01 biofilms

**FIGURE 3:**
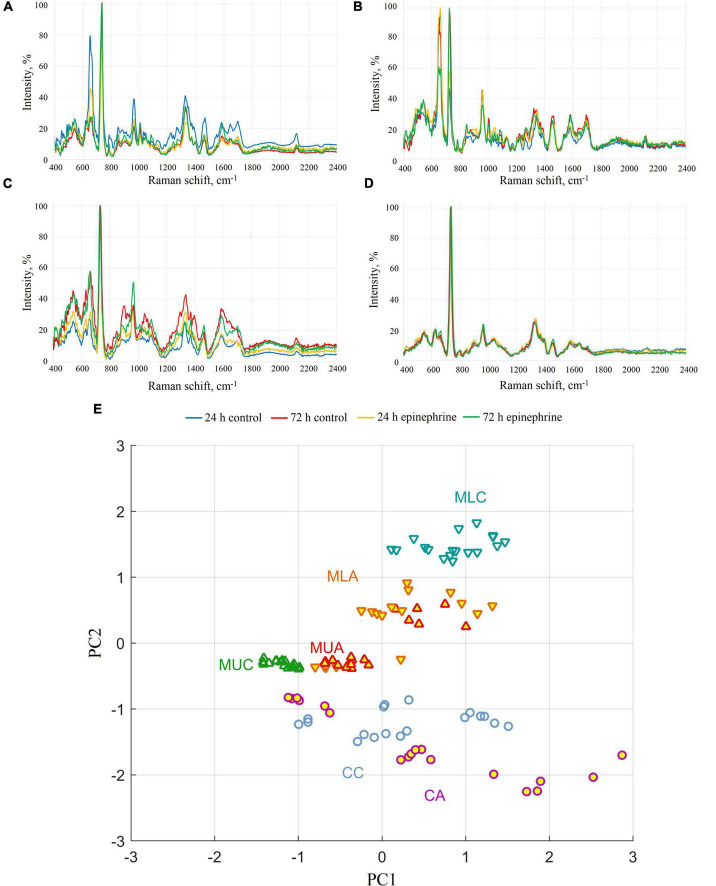
SERS spectra of *M. luteus* C01 biofilms. **(A)** Upper phase of the matrix. **(B)** Lower phase of the matrix. **(C)** Intact biomass. **(D)** Biomass after matrix extraction. **(E)** Principal component analysis of SERS spectra for 72 h samples (CC, control cells; CA, epinephrine-treated cells; MUC, control upper phase of the matrix; MUA, epinephrine-treated upper phase of the matrix; MLC, control lower phase of the matrix; MLA, epinephrine-treated lower phase of the matrix).

The spectra obtained show the differences in matrix composition in comparison to the biomass after matrix isolation (Figure 3). Here, most of the peaks were found in the samples from matrix phases and intact biomass (Figures 3A–C), which additionally verifies the isolation of the matrix and a cell suspension. The results from the PCA analysis of the differences in the samples are depicted in the Supplementary Figure 5.

The analysis of the samples revealed that the spectra of the biomass after matrix extraction practically did not differ from each other. In only the 72 h epinephrine-treated biomass samples after matrix extraction, the peak at 662 cm^–1^ was slightly higher than in the other biomass samples (Figure 3D). This peak indicates guanine and proteins, which is in agreement with the results of the Dische method. In the group of intact biomass samples, differences were observed in the peak at 905 cm^–1^; however, in the control intact 24 h biomass samples, the difference was much less pronounced (Figure 3C). This peak is related to AMP/proteins. In the upper phase matrix samples (Figure 3A), the main difference was observed in the peaks at 650–660 cm^–1^ (two overlapping peaks). These peaks refer to guanine/xanthine/proteins. The intensity of the peak fell from 24 h to 72 h of incubation in the control samples and epinephrine samples. In the lower phase matrix samples (Figure 3B), we observed a change in the ratio between the 650–660 cm^–1^ group and the peaks at 725 cm^–1^ (stretching vibrations of the adenosine ring) in the control and epinephrine samples. The spectra of the epinephrine samples were otherwise similar, although there was still a slight difference in the region of 1330–1340 cm^–1^; however, it was also present in the control. In the 72 h samples, the peak was slightly shifted to the left. In the control samples from the lower phase of the 72 h biofilm matrix, a peak at ∼1000 cm^–1^ related to the respiratory vibration of the phenylalanine ring (Willets, 2009) appeared to be significantly increased. The principal component analysis of the SERS spectra (Figure 3E) showed that cells, upper phase of the matrix, and lower phase of the matrix are discernible by SERS spectra (mostly by PC2 component). In terms of spectra similarity, cells are closer to the upper phase of the matrix, than to the lower phase. The treatment with epinephrine shifts the SERS spectra and eliminates differences between lower and upper phases of the matrix, but cells remain discernible, nevertheless. See also the principal component analysis in the Supplementary Figure 5.

### Polysaccharides in the *Micrococcus luteus* C01 biofilm matrix

Polysaccharides were isolated from the 24 h (control), 24 h (epinephrine), 72 h (control), and 72 h (epinephrine) *M. luteus* C01 matrix groups and purified via GPC on a TSK HW-40 (S) column. The sugar analysis of the alditol acetates performed using GLC revealed a similar composition in all samples studied, which comprised mannose, glucose, and GlcNAc (Supplementary Figure 6). The D configuration of the monosaccharides was determined by GLC of the acetylated (*S*)-2-octyl glycosides (Leontein and Lönngren, 1993).

The NMR spectra (Figures 4, 5 and Supplementary Figures 7–20) suggested that all matrix samples were mixtures of polysaccharides; thus, the polysaccharides were partially separated using anion-exchange chromatography with DEAE-Trisacryl M and a stepwise gradient of 0.005 to 0.5 M hydrogen sodium phosphate buffer (pH 6.3). As result, two fractions were obtained from matrix 24 h (control) samples: one eluted in 0.1 M phosphate buffer and the other eluted in 0.5 M phosphate buffer. Both fractions were studied using 1D and 2D NMR spectroscopy.

**FIGURE 4:**
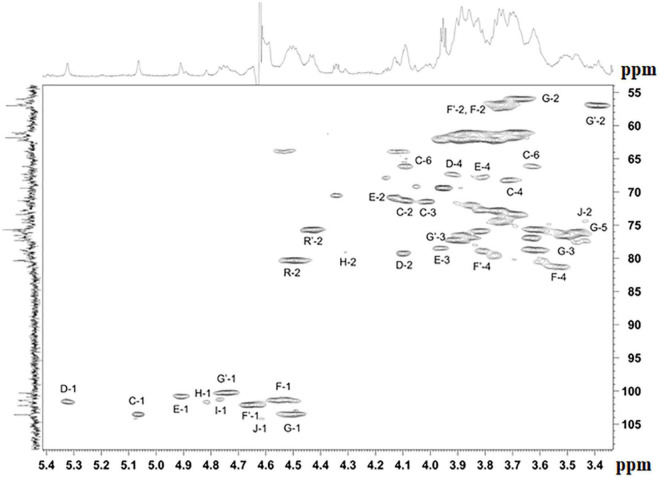
Parts of the 2D ^1^H,^13^C edHSQC spectrum of *M. luteus* C01 matrix polysaccharides [24 h (control)] eluted in 0.1 M phosphate buffer. The corresponding parts of the ^1^H and ^13^C NMR spectra are shown along the horizontal and vertical axes, respectively. Numbers refer to protons and carbons of sugar units designated by the letters shown in Table 5.

**FIGURE 5:**
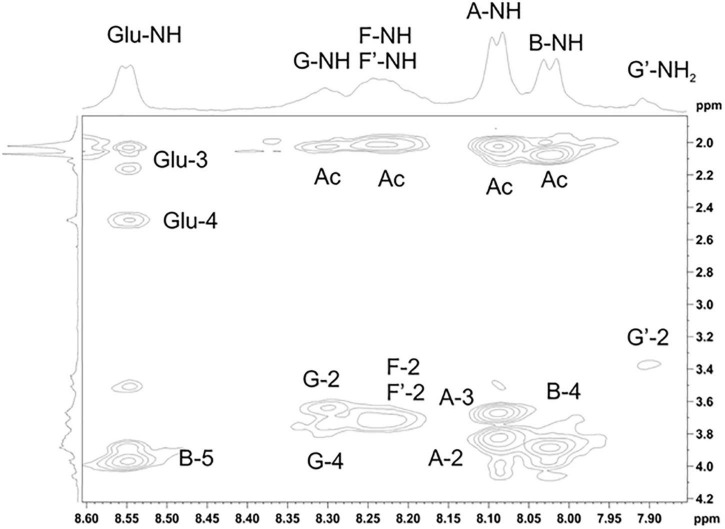
Parts of the 2D ROESY spectrum of *M. luteus* C01 matrix polysaccharides [24 h (control)] eluted in 0.1 M phosphate buffer measured in a 9: 1 H_2_O/D_2_O mixture. The corresponding parts of the ^1^H NMR spectra are shown along the horizontal and vertical axes, respectively. Numbers refer to the protons and carbons of sugar units designated by the letters shown in Table 5.

#### Fraction eluted in 0.5 M phosphate buffer [matrix 24 h (control) 0.5 M]

**FIGURE 6:**
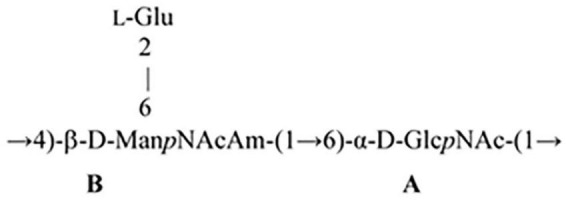
Structure of *M. luteus* C01 cell wall polysaccharide.

The 1D and 2D NMR spectra of the 24 h (control) 0.5 M matrix sample showed signals for anomeric atoms at δ_*H*_ 4.86–5.36 and δ_*C*_ 98.3–101.5 (Supplementary Figure 7). COSY, TOCSY, and HSQC experiments allowed us to determine all direct correlations between carbons and protons and complete the assignment of cross-peaks to each of the sugars (Table 5) and spin systems. GlcNAc (A), ManNAcA (B), and Glu (glutamic acid) were identified by their characteristic proton–proton couplings and carbon chemical shifts when compared to the data averaged and stored in the GODDESS carbohydrate NMR database (Kapaev and Toukach, 2016). The L configuration of glutamic acid was determined by GLC of the acetylated (S) 2,5-di-octyl glutaminic ester. The assignment within each spin system was performed using COSY, and the relative configurations of the monosaccharides were determined based on ^3^*J*_*H,H*_ coupling constants. The ^1^H and ^13^C NMR chemical shift data combined with data from linkage and sequence analyses using ROESY and ^1^H,^13^C HMBC experiments (Table 6) revealed the following structure of PSI (Figure 6). This structure was established earlier as the cell wall polysaccharide of *M. luteus* C01 (Zdorovenko et al., 2021).

**TABLE 5:** ^1^H and ^13^C NMR chemical shifts in cell wall polysaccharides of *M. luteus* C01 (δ, ppm).

Residue	C-1 *H-1*	C-2 *H-2*	C-3 *H-3*	C-4 *H-4*	C-5 *H-5*	C-6 *H-6,6*′	Fit
PSI							GOF 0.79, RMS 1.27, LC 1.000
HOOC-CH-CH_2_-CH_2_-COOH Glu | NH |	176.4	55.5 *4.31*	28.5 *2.16*, *2.02*	31.7 *2.47*	178.0 *4.42*		

6) →4)-β-Man*p*NAcAm-(1→ B	101.5 *4.86*	54.6 *4.52*	74.1 *4.07*	73.6 *3.91*	76.5 *3.99*	170.6	

→6)-α-Glc*p*NAc-(1→ A	98.3 *5.36*	55.0 *3.82*	71.8 *3.70*	70.8 *3.44*	72.1 *3.52*	69.3 *3.91, 3.87*	

PSII							GOF 0.27, RMS 0.38, LC 1.000

→6)-α-Man*p*-(1→ C	103.6 *5.06*	71.3 *4.08*	72.0 *3.85*	67.4 *3.91*	72.7 *3.82*	66.2 *4.08, 3.62*	

→2)-α-Man*p*-(1→ D	101.7 *5.32*	79.3 *4.09*	71.5 *4.01*	68.3 *3.71*	74.6 *3.75*	62.3*^a^* *3.89, 3.75*	

→3)-α-Man*p*-(1→ E	100.9 *4.90*	70.9 *4.12*	78.5 *3.96*	67.8 *3.81*	74.1 *3.72*	61.8*^a^* *3.89, 3.78*	

PSIII/PSIII’							GOF 0.65 RMS 1.34, LC 1.000 (PSIII) GOF 0.58 RMS 1.36, LC 1.000 (PSIII’)*^b^*

→4)-β-D-Glc*p*NAc-(1→ F	101.3 *4.51*	56.8 *3.73*	73.2 *3.70*	81.3 *3.51*	76.6 *3.50*	61.1*^a^* *3.84, 3.70*	

→4)-β-D-Glc*p*NAc-(1→ F’	102.1 *4.66*	57.3 *3.75*	74.4 *3.74*	78.9 *3.81*	75.6 *3.62*	61.8*^a^* *3.84, 3.70*	

→4)-β-D-Glc*p*NAc3R-(1→ G	103.4 *4.48*	55.9 *3.66*	78.8 *3.60*	76.7 *3.85*	76.3 *3.46*	61.1*^a^* *3.86, 3.68*	

→4)-β-D-Glc*p*N3R-(1→ G’	100.3 *4.74*	57.0 *3.39*	77.2 *3.90*	76.0 *3.82*	76.9 *3.63*	62.3*^a^* *3.86, 3.68*	

R-Lac-(2→ R	182.6	80.6 *4.49*	19.7 *1.35*				

R-Lac(2→ R’	175.8	75.9 *4.43*	18.0 *1.45*				

PSIV							GOF 0.23 RMS 0.31, LC 1.000*^c^*

→2)-β-Man*p*-(1→ H	101.6 *4.81*	79.1 *4.31*	73.3 *3.69*	68.1 *3.60*	77.7 *3.46*		

→4)-β-Man*p*-(1→ I	101.4 *4.76*	71.4 *4.12*	72.8 *3.82*	78.1 *3.83*	76.4 *3.49*		

→4)-β-Glc*p*-(1→ J	104.3 *4.61*	74.4 *3.42*	75.2 *3.69*	79.6 *3.67*	76.3 *3.54*		

PSIII/PSIII’ Smith							

→4)-β-D-Glc*p*NAc-(1→ F	101.3 *4.52*	56.9 *3.74*	73.4 *3.71*	81.3 *3.52*	76.6 *3.49*	61.9*^a^* *3.84, 3.69*	

→4)-β-D-Glc*p*NAc-(1→ F’	102.0 *4.64*	57.4 *3.74*	72.8 *3.78*	79.2 *3.78*	75.7 *3.59*	61.9*^a^* *3.84, 3.69*	

→4)-β-D-Glc*p*NAc3R-(1→ G	103.4 *4.50*	56.0 *3.69*	79.4 *3.62*	76.7 *3.86*	76.2 *3.47*	61.1*^a^* *3.86, 3.68*	

→4)-β-D-Glc*p*N3R-(1→ G’	98.9 *4.88*	57.8 *3.16*	78.9 *3.89*	76.9 *3.54*	76.8 *3.63*	61.1*^a^* *3.86, 3.68*	

R-Lac-(2→ R	181.6	79.9 *4.49*	19.8 *1.36*				

R-Lac-(2→ R’	182.1	81.3 *4.07*	18.9 *1.25*				

CH_3_CON (A) at δ_H_ 2.09, δ_C_ 23.4 and 176.8.

CH_3_CON (B) at δ_H_ 2.05, δ_C_ 23.4 and 175.6.

CH_3_CON (F) at δ_H_ 2.02, δ_C_ 23.5 and 175.9.

CH_3_CON (G) at δ_H_ 2.04, δ_C_ 23.5 and 175.9.

^a^The assignment of the unsubstituted –CH_2_OH groups (C-6 of hexoses) was performed tentatively in a range of δ 61.1–62.3 ppm and adjusted from calculations.

^b^Results for PSIII’ are provided from a simulation with muramic acid and N-acetyl-glucosamine used as a monomeric composition.

^c^For the simulation of PSIV, an average value of 62.2 ppm was used for all C-6 chemical shifts.

**TABLE 6:** Correlations for H-1 and C-1 in the 2D ROESY and ^1^H,^13^C HMBC spectra of the 24 h (control) matrix polysaccharides of *M. luteus* C01.

Anomeric atom in sugar residue (δ)	Correlation(s) to atoms in sugar residue(s) (δ)	
	
	ROESY	HMBC
**PSI**		
**A** H-1 (5.32)	**A** H-2 (3.84), **B** H-3 (4.08), H-4 (3.91), H-5 (3.99)	**B** C-4 (74.4), **A** C-3 (72.0), C-5 (72.3)
**A** C-1 (98.6)		**B** H-4 (3.91)
**B** H-1 (4.86)	**B** H-3 (4.08), H-5 (3.99), **A** H-6 (3.91)	**B** C-2 (54.6), **A** C-6 (69.3)
**B** C-1 (101.3)		**A** H-6 (3.91), **B** H-2 (4.53)
**PSII**		
**C** H-1 (5.06)	**D** H-2 (4.09), H-3 (4.01), **C** H-2 (4.08)	**D** C-2 (79.3), **C** C-3 (72.0), C-5 (72.7)
**C** C-1 (103.6)		**D** H-2 (4.09)
**D** H-1 (5.32)	**D** H-2 (4.08), **E** H-3 (3.96), H-4 (3.81)	**E** C-3 (78.5), **D** C-5 (74.6), C-3 (71.5)
**D** C-1 (101.7)		**E** H-3 (3.96)
**E** H-1 (4.90)	**E** H-2 (4.12), **C** H-6 (3.62)	**C** C-6 (66.2), **E** C-3 (78.5), C-5 (74.1)
**E** C-1 (100.9)		**C** H-6 (4.08)
**PSIII, PSIII’**		
**F** H-1 (4.51)	**F** H-3 (3.70), H-5 (3.50), **G** H-4 (3.85)	
**G** H-1 (4.48)	**G** H-2 (3.66), H-3 (3.60), H-5 (3.46), **F** H-4 (3.51)	
**F’** H-1 (4.66)	**F’** H-3 (3.74), H-5 (3.62), **G’** H-4 (3.82)	
**G’** H-1 (4.74)	**G’** H-3 (3.90), H-5 (3.63), **F’** H-4 (3.81), H-3 (3.74)	
**PSIII (Smith), PSIII’ (Smith)**		
**F_Sm_** H-1 (4.52)	**F_Sm_** H-3 (3.71), H-5 (3.49), **G_Sm_** H-4 (3.86)	
**G_Sm_** H-1 (4.50)	**G_Sm_** H-3 (3.62), H-5 (3.47), **F_Sm_** H-4 (3.52)	
**F’_Sm_** H-1 (4.64)	**F’_Sm_** H-3 (3.78), H-5 (3.59), **G’_Sm_** H-4 (3.80)	
**G’_Sm_** H-1 (4.88)	**G’_Sm_** H-3 (3.89), H-5 (3.63), **F’_Sm_** H-4 (3.78)	
**PSIV**		
**H** H-1 ()	**H** H-2 (4.31), H-3 (3.69), H-5 (3.46), **I** H-4 (3.83)	
**I** H-1 ()	**I** H-2 (4.12), H-3 (3.82), H-5 (3.49), **J** H-4 (3.67)	
**J** H-1 ()	**J** H-3 (3.69), H-5 (3.54), **H** H-2 (4.31)	

#### Fraction eluted in 0.1 M phosphate buffer [matrix 24 h (control) 0.1 M]

The NMR spectra of the fraction eluted in 0.1 M buffer *[matrix 24h (control) 0.1M]* showed signals of varying intensities, indicating structural heterogeneity (Figure 4 and Supplementary Figures 7–20). The ^1^H NMR spectrum contained signals for anomeric protons at δ 4.50–5.32, a CH_3_-C group (H-3 of lactic acid) at δ 1.35, other sugar ring protons at δ 3.39–4.50, *N*-acetyl groups at δ 2.02–2.09, and CH_3_-groups at δ 1.36–1.44. The ^13^C NMR spectrum contained signals for anomeric carbons at δ 100.3–103.6, a number of OCH_2_ groups at δ 61.06–66.2, nitrogen-bearing carbons at δ 56.0–57.3, methyl groups at δ 19.8 (C-3 of lactic acid), one COOH group (C-1 of lactic acid) at δ 182.1, other sugar ring carbons at δ 64.7–78.1, and *N*-acetyl groups at δ 23.5 (CH_3_) and 175.9 (CO).

The spectra were assigned using ^1^H,^1^H COSY, TOCSY, ROESY, ^1^H,^13^C HSQC, HSQC-TOCSY, and HMBC experiments (Table 5 and Figure 4). It was found that the sample contained five mannose (**C**, **D**, **E, H, I**), four glucosamine (**F**, **F’**, **G, G’**), and one glucose (**J**) residue. The α-linkage (**C**, **D**, **E**) and β-linkages of Man (**H**, **I**), Glc*p*N (**F**, **G**), and Glc (**J**) were established using characteristic C-5 chemical shifts (Table 5) and the presence of H-1/H-2 cross-peaks for units **C**, **D**, and **E** and H-1/H-5 cross-peaks for units **F-J** in the ROESY spectrum (Table 6). In this way, the β-configuration of the mannose residues (**H** and **I**), GlcNAc (**F** and **G**) and glucose (**J**) was inferred by a comparison of the C-5 chemical shifts at 76.4–77.7 with the published values of δ 73.3 and 77.0 for α- and β-Manp, 72.5 and 76.8 for α- and β-GlcNAc, and 72.4 and 76.8 for α- and β-Glc, respectively (Lipkind et al., 1988), as averaged and stored in the GODDESS carbohydrate NMR database (Kapaev and Toukach, 2016).

The spin systems of amino sugars were distinguished by correlations between protons at nitrogen-bearing carbons (H-2) and the corresponding carbons (C-2) with a characteristic chemical shift. The spin systems of four acetylated and free GlcN (**F, F’, G, G’**) and Glc **(J)** were identified via the correlations of H-1 with H-2 to H-5 in the TOCSY experiment. The assignment of mannose residues was based on the correlations of H-1 to H-2 and H-2 to H-6 in the TOCSY spectrum.

Downfield displacements were observed for the C-3 and C-4 signals of GlcNAc (**G, G’**), C-4 of GlcNAc (**F, F’**), C-4 of Man (**I**), C-4 of Glc (**J**), C-2 of Man (**H, D**), C-3 of Man (**E**), and C-6 of Man (**C**) (Table 5), when compared to their positions in the corresponding non-substituted compounds (Lipkind et al., 1988; Kapaev and Toukach, 2016). These data defined the linkage pattern of the monosaccharide units.

The sequence of the monosaccharides was determined by correlations between the anomeric protons and protons or carbons at linkage positions in the ROESY and HMBC spectra, respectively (Table 6).

A strong cross-peak between H-2 of the lactic acid residue and H-3 of GlcNAc (**G, G’**) at δ 4.49/3.60 and 4.43/3.90, respectively, indicated the location of an ether linkage at position 3, which was verified by a relatively lowfield position of the signal for GlcNAc (**G, G’**) C-3 at δ 78.8 and 77.3 (compared to δ 73.2 for non-substituted GlcNAc C-3), thus confirming the presence of the GlcN3Lac residue.

The ^1^H NMR spectrum of the matrix polysaccharides measured in a 9: 1 H_2_O/D_2_O mixture showed signals for NH protons. Using COSY, TOCSY, and ROESY experiments, three of these signals were assigned to the NH groups of units **A** and **B** and glutamic acid from PSI; the others were assigned to the NH/NH_2_ groups of units **G**, **G’**, **F,** and **F’**. The ROESY spectrum (Figure 5) showed correlations with the acetyl groups, thus indicating the *N*-acetylation of all sugar residues at position 2. An exception was the signal of the NH proton at δ 7.91. This signal correlated with H-2 of the G′ residue and lacked correlations with any acetyl groups in the ROESY and HMBC spectra. Thus, residue G′ was not *N*-acetylated at position 2.

The α-glycosylation effects on C-1 of GlcNAc3RLac (**G**) and C4 of D-GlcNAc (**F**) and the β-glycosylation effect on C-3 of D-GlcNAc F (+7.55, +10.24, and −2.01 ppm, respectively) indicated the same absolute configuration of the linked monosaccharide residues, i.e., the D-configuration of a sugar moiety of GlcNAc3RLac [as compared with published data (Shashkov et al., 1988) for the same and different absolute configurations].

Thus, the fraction eluted in 0.1 M buffer contained several polysaccharides with the following structures:

→6)-α-D-Man*p*-(1→2)-α-D-Man*p*-(1→3)-α-D-Man*p*-(1→            PSII

**C**                      **D**                      **E**

**G          F**

→4)-β-D-Glc*p*N**R**-(1→4)-β-D-Glc*p*NAc-(1→                         PSIII

R-Lac(2→⌋3

→2)-β-D-Man*p*-(1→4)-β-D-Man*p*-(1→4)-β-D-Glc*p*-(1→       PSIV

**H       I       J**

where R is Ac (PSIII) or H (PSIII’)

To further confirm the polysaccharide structures, the polysaccharides from the fraction eluted in 0.1 M buffer were subjected to Smith degradation, and the products from the mild acid hydrolysis of the degraded polymers were fractionated via GPC on a TSK HW-40 column. As a result, the polysaccharides PSIII and PSIII’ and oligosaccharides were isolated. They were studied using 1D and 2D NMR spectroscopy, including ROESY, as described above for the polysaccharides (for the assignment of the ^1^H and ^13^C NMR signals, see Table 5). As a result, the structures of PSIII and PSIII’ were confirmed (Tables 5, 6).

In addition to the major signals tabulated in Table 5, the NMR spectra showed a number of minor signals, which were not assigned owing to their low intensities.

The assignment of all spectra was confirmed with the GODDESS carbohydrate NMR simulator (Kapaev et al., 2014). The unassigned ^13^C NMR spectra and the monomeric composition were fed to the GRASS module (Kapaev and Toukach, 2018) of the Carbohydrate Structure Database (Toukach and Egorova, 2016) to predict the structure topology, anomeric configurations, cycle sizes, linkage pattern, and the sequence of residues. For PSI, an additional HMBC-based restriction was applied to filter out all glutamic acid esters from the results, leaving only its amides with a uronic acid. In all cases, the elucidated structure coincided with the structure predicted as a best match by GRASS. The goodness of fit [explained elsewhere (Kapaev and Toukach, 2018)], root-mean-square deviation, and linear correlation coefficient are provided for every polysaccharide in the rightmost column of Table 5. The difference in goodness of fit between the best match and the second-ranked structure varied from 0.1 to 0.5, confirming the trustworthiness of the ranking, except for PSII, where this difference was not significant (0.03). For all structures, the signal assignments corresponded to those obtained from the interpretation of the NMR spectra. The prediction logs and ranked lists of candidate structures are available at *http://csdb.glycoscience.ru/grass.html*, under the job name mask *mluteusC02_PS*.

PSII is structurally close to a mannan of the cell wall polysaccharide of *M. luteus* C01 (Zdorovenko et al., 2021).

PSIII is not unique among bacterial polysaccharides. It was found in *P. alcalifaciens* O45, O3, O24, and O38 (Ovchinnikova et al., 2012). PSIII and PSIII’ were found in the endospore peptidoglycan of *Bacillus subtilis* 168 (Atrih et al., 1996). PSIII is a carbohydrate base of a classical peptidoglycan that was earlier found to be a cell wall component of Gram-positive bacteria (Knirel and Valvano, 2013). According to a search in the Carbohydrate Structure Database (Toukach and Egorova, 2016), the structure of PSIV is unique among the known polysaccharides of any microorganisms, although a related structural fragment where both mannoses are 1,4-linked had 28 occurrences in a prokaryotic glycome.

In the matrix 24 h (control), the polysaccharides were found in a PSI:PSII:PSIII:PSIII’:PSIV ratio of ∼3.3:1.0:3.0:2.3:0.4. This was determined by a comparison of the integral signal intensities in the ^1^H NMR spectrum.

A comparative study of the NMR spectra of polysaccharides from the matrix 24 h (epinephrine) and matrix 24 h (control) samples showed the presence of the polysaccharides PSI, PSII, PSIII, and PSIII′ in a ratio of ∼3.8:1.0:4.2:2.5. PSIV was not found in matrix 24 h with epinephrine samples.

A comparative study of the NMR spectra of polysaccharides from the matrix 72 h (control) and matrix 72 h (epinephrine) samples with matrix 24 h (control) showed the presence of the polysaccharides PSI, PSII, and PSIII in ratios of ∼1.9:1:3 and ∼4:1:4.5, respectively. PSIII’ and PSIV were not found in the 72 h (control) and 72 h (epinephrine) matrices.

## Discussion

It has been known for decades that prokaryotic and eukaryotic cells are extremely complex systems containing tightly interrelated molecular machineries and processes. As such, any internal or external impact may shift the balance and provoke a cascade of reactions, which are sometimes difficult to trace fully. Human hormones seem to trigger global physiological changes in cells and in biofilms. Recently, the complex effect of a number of hormones on monospecies biofilms and mixed-species communities of some human commensal bacteria has been shown (Clabaut et al., 2021; Mart’yanov et al., 2021; Ovcharova et al., 2021; Diuvenji et al., 2022; Kiseleva et al., 2022; Scardaci et al., 2022). Our work on *M. luteus* C01 showed changes in the expression of seven genes in biofilms in the presence of 4.9 nM epinephrine (Gannesen et al., 2021). Some Fe-S cluster genes were upregulated, while genes for the lysin transporter LysE, chaperone GroES, short-chain alcohol dehydrogenase, and the potential c-di-GMP phosphotransferase were downregulated. Despite the fact that very few genes with differences in expression were revealed using RNAseq and qPCR earlier, the changes in the matrix proteome were much more extensive especially after 24 h of incubation in immature biofilms. Hence, we propose that hormones act as regulators of some reaction cascades that are triggered either by some posttranslational modifications of regulatory proteins or by some unknown signal system. Potentially, the c-di-GMP phosphotransferase-like protein described earlier (Gannesen et al., 2021) may be a key player in the epinephrine sensitivity of *M. luteus* C01. The analysis of the proteome revealed changes in numerous matrix proteins, most of which had catalytic activity. There are certain points that should be given attention. First, in the 24 h immature biofilm matrix, there was a higher amount of proteins with the ability to bind to polysaccharides (some ABC family proteins, peptidoglycan-binding protein A0A5E8QCZ2). This could be one reason for the stabilization of the matrix in the immature biofilms and the resulting higher matrix amount after 72 h of incubation. Second, similar to the matrix of *C. acnes* (Gannesen et al., 2019), there were hundreds of proteins in the micrococcal matrix that seemed to be present due to cell autolysis during biofilm maturation. For instance, as in C. acnes, we found EF-Tu in the matrix of *M. luteus* C01. These proteins could also be a part of a catalytic matrix compartment; however, their actual functions (for instance, in the case of ribosomal proteins) are still a matter of further investigations. Nevertheless, such an extensive shift in the amount of catalytic proteins, which are responsible for different intracellular processes, demonstrates the substantial changes inside the cells in presence of the hormone, verifying the multitargeted and global effect of epinephrine on *M. luteus* and potentially providing evidence of such an effect of all hormones on human commensal bacteria.

The changes in the biofilm matrix lipidome in the presence of epinephrine are also worth noting. After 24 h of incubation, there was a significant decrease in the amount of lipids in the presence of epinephrine; in mature biofilms, the hormone slightly increased the lipid concentration. However, in general, the maturation of biofilms led to a decrease in lipid amounts in *M. luteus* biofilms. Moreover, the phospholipid compartment of the lipidome was absent in the presence of epinephrine after 24 h of incubation and in all 72 h samples. Hence, the hormone either significantly affected lipid synthesis in *M. luteus* cells (especially the synthesis of phospholipids) or stimulated lipid consumption by the cells. The last explanation may be based on the possible intensification of *M. luteus* C01 metabolism in the presence of the hormone (Gannesen et al., 2021).

As was demonstrated in the present research, the changes in biofilm matrix composition in the presence of epinephrine were rather extensive. First, our hypothesis about the role of extracellular DNA in the stabilization of the matrix in *M. luteus* C01 was verified experimentally. The amount of DNA in the matrix of mature 72 h biofilms was higher than in the control according to the Dische method results and SERS analysis. In our previous work, the treatment of mature 72 h biofilms with DNAse I resulted in the removal of the epinephrine stimulatory effect (Gannesen et al., 2021). Here, we showed that in the presence of epinephrine, there were more sugars and DNA in the mature biofilm matrix, and eDNA seemed to be an essential component for matrix stability in *M. luteus* C01. Interestingly, an increased amount of the protein A0A653QWC7, which is responsible for DNA protection during starvation, could also be a reason for the increased DNA amount. After such a long incubation period, the biofilms of *M. luteus* came into a late life cycle phase, and the model system did not allow them to disperse into a liquid medium. Hence, the starvation processes could start at this stage, and additional DNA protection is a reasonable way to protect the matrix. In addition, it is worth noting that the total amount of proteins was not changed in the mature or immature biofilms in the presence of epinephrine. Moreover, there were less proteins in mature biofilms when compared to the immature biofilms. Thus, the structure of the matrix either allowed proteins to diffuse better in the 72 h biofilms or the cells started to use the proteins as an additional nutrient source (this is an additional point to the starvation hypothesis).

The first in-depth investigation of polysaccharides in the *M. luteus* C01 matrix revealed five types of polymers that contained mannose, glucose, glucose amine, and *N*-acetylglucose amine. Interestingly, the hormone affected the synthesis of PSIV—the only minor polymer containing glucose in its chain alongside mannose. Thus, it could be proposed that epinephrine somehow affected the synthesis of at least some glucose-containing polysaccharides in *M. luteus*, which could be related to the downregulation of an unknown protein with EAL domains—a potential c-di-GMP phosphotransferase (Gannesen et al., 2021). This hypothesis still must be approved or denied in future.

Taken together, our results establish that the effect of epinephrine at a concentration of 4.9 nM, which is close to the physiological blood plasma level, on *M. luteus* C01 biofilms is a complex phenomenon. Despite the fact that only seven genes were found to have changed expression patterns in the presence of the hormone, the resulting effect was extensive and based on intricate relationships between cell components. Although we found some proteins with changed concentrations (especially interesting was histidine kinase A0A5E8QFN5 in mature biofilms), it is very difficult to propose the key epinephrine targets and reaction cascades inside cells that could result in such extensive changes. However, we suggest the main directions of epinephrine impact are as follows: (i) cell metabolism intensification; (ii) glucose-mannose polymer synthesis; and (iii) eDNA-based matrix stabilization. Due to changes in the DNA-polysaccharide matrix structure, the intensification of cell metabolism (potentially via the stimulation of enzymes with Fe-S clusters), and intracellular protein assembly processes, the proteome of the matrix could be changed. Thus, a focus of future investigations should be searching for epinephrine targets and sensors in *M. luteus* cells and distinguishing the reaction cascades in the presence of the hormone. Nevertheless, the effect of epinephrine on *M. luteus* C01 biofilms seems to be global, and we suggest that each hormone has an effect to the same extent on bacterial cells and biofilms.

## Data availability statement

The mass spectrometry proteomics data have been deposited to the ProteomeXchange Consortium via the PRIDE (Perez-Riverol et al., 2022) partner repository with the dataset identifier PXD035739.

## Author contributions

AG contributed to the conceptualization, methodology design, matrix isolation, quantitative biochemistry tests, LDH tests, general data analysis, article writing, and project administration. RZ contributed to the proteomic samples preparation, proteomic data obtaining, data analysis, and proteomic part writing. EZ contributed to the polysaccharides research, NMR research, NMR data analysis, and NMR part writing. AK contributed to the biofilm growth, matrix isolation, quantitative biochemistry tests, and article writing. El and OD contributed to the lipid research, lipid chromatography studies. VT contributed to the lipid research, lipid chromatography studies, and lipid part writing. MG and AN contributed to the SERS investigation, SERS data analysis, and SERS part writing. MO and EN contributed to the biofilm growth, matrix isolation, and quantitative biochemistry tests. SM and EB contributed to the data analysis and article writing. AS and AD contributed to the polysaccharides research, NMR research, and NMR part writing. MZ contributed to the biofilm growth, matrix isolation, and data analysis. PT contributed to the NMR research, NMR data analysis, and NMR part writing. VP contributed to the conceptualization, article writing, and project administration. All authors contributed to the article and approved the submitted version.

## Footnotes

1
https://www.ncbi.nlm.nih.gov/bioproject/
