# Supplementary material for: Epinephrine extensively changes the biofilm matrix composition in Micrococcus luteus C01 isolated from human skin
Source: Front Microbiol. 2022 Sep 20;13:1003942. doi: 10.3389/fmicb.2022.1003942 (PMC9530943; doi:10.3389/fmicb.2022.1003942)
Supplement: Supplementary file 1 [file Data_Sheet_1.DOCX]

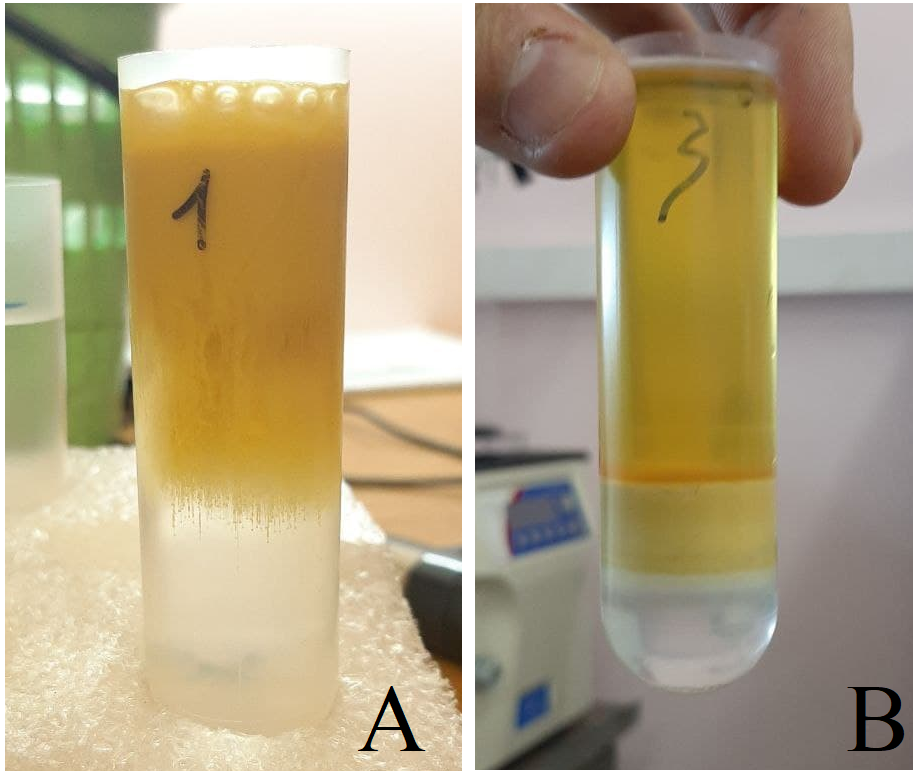


Supplementary Figure 1. A photo of a 24 h biofilm biomass and matrix before the centrifugation (A) and after the centrifugation (B).


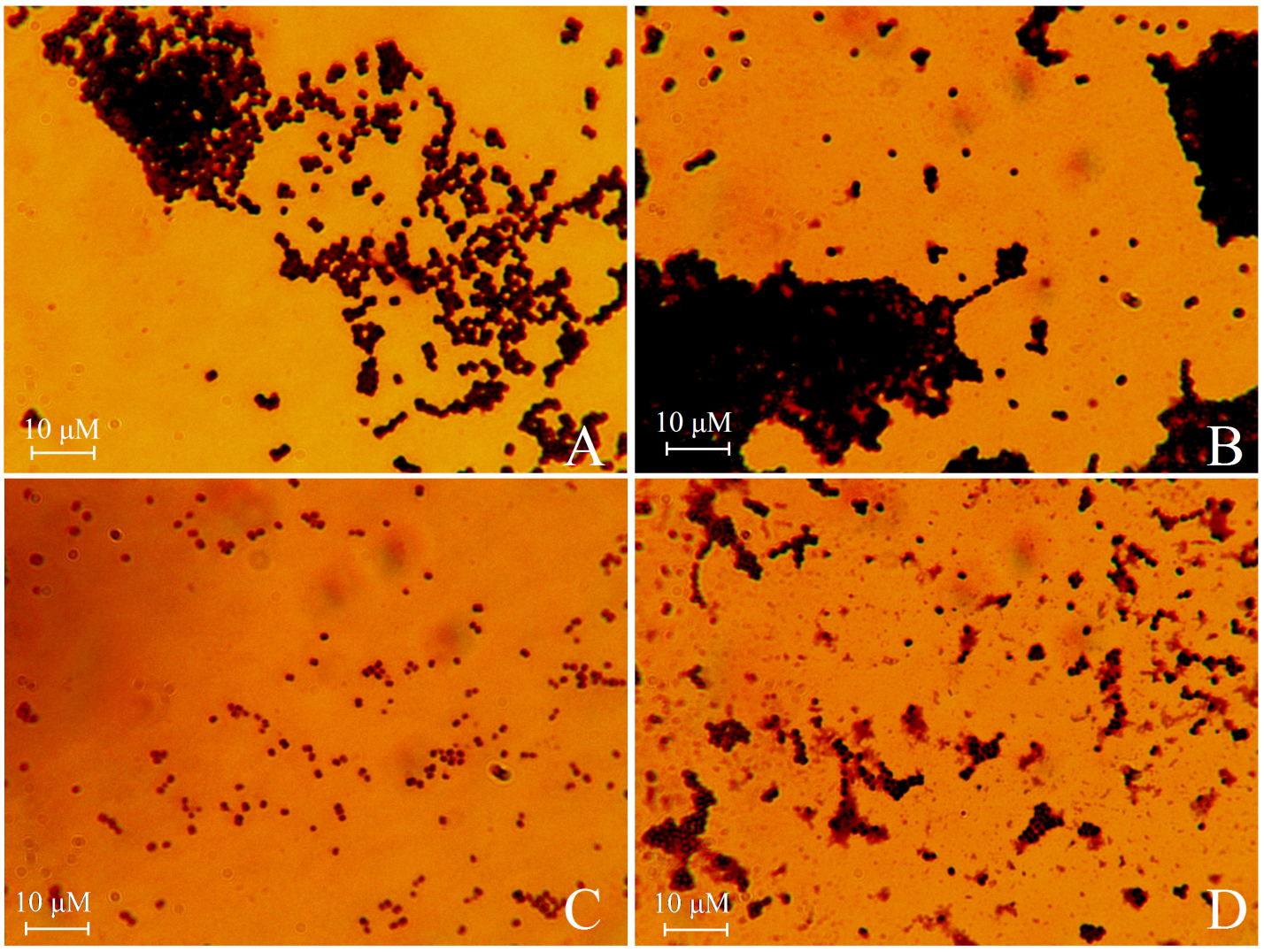


Supplementary Figure 2. Cell disruption control with light microscopy. A- biomass before the sonication. B – biomass after sonication (aggregates). C – biomass after sonication (less stained cells). D - a turbid layer above the cell biomass after centrifugation.


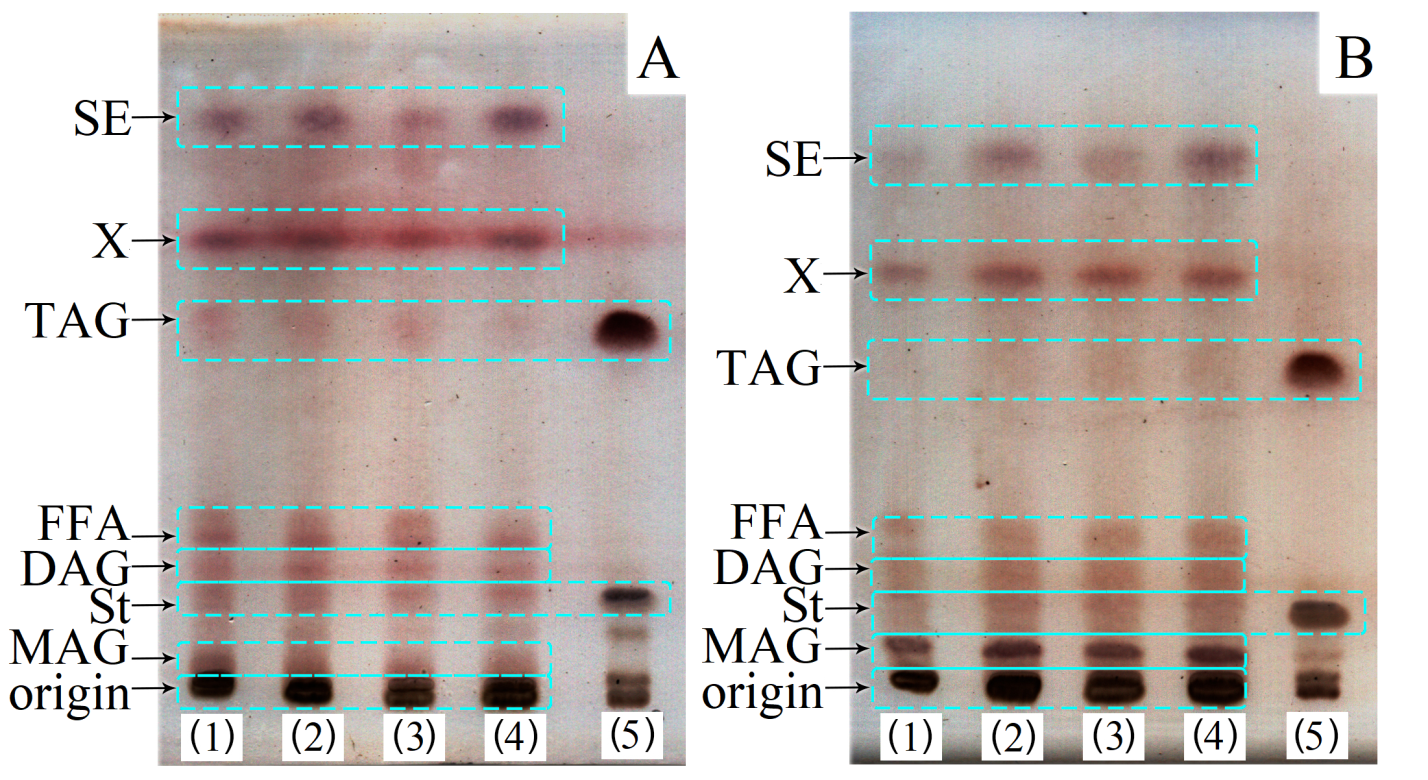


Supplementary Figure 3. Identification of neutral lipids of the biomass (A) and matrix (B) of *M. luteus* C01 biofilms. The components were identified by standard substances. (1) — control 24h; (2) — control 72h; (3) — epinephrine 24h; (4) — epinephrine 72h; (5) — reference standards for sterols and triacylglycerols. Abbreviations: DAG - diacylglycerols; FFA - free fatty acids; MAG - monoacylglycerols; St - sterols; SE - sterol esters; TAG - triacylglycerols; X - unidentified lipid.

Supplementary Figure 4. Identification of the polar lipids. A-D - thin-layer chromatograms of the polar lipids of the biomass. The components were visualized by staining with 5 % sulfuric acid in ethanol and heating at 180°C for 15 min. (A) — control 24h; (B) — control 72h; (C) — epinephrine 24h; (D) — epinephrine 72h. E-H - Identification of the polar lipids of the biomass. The components were visualized by molybdenum blue (E); α-naphthol (F); ninhydrin (G); Dragendorff (H). I-L Thin-layer chromatograms of the polar lipids of the matrix. The components were visualized by staining with 5 % sulfuric acid in ethanol and heating at 180°C for 15 min. (I) — control 24h; (J) — control 72h; (K) — epinephrine 24h; (L) — epinephrine 72h. Abbreviations: DPG - diphosphatidylglycerol; PC - phosphatidylcholine; PG - phosphatidylglycerol; PL - unidentified phosholipid; AL – unidentified aminolipid; APL - unidentified aminophosholipid; GL - unidentified glycolipid; X — unidentified lipid.


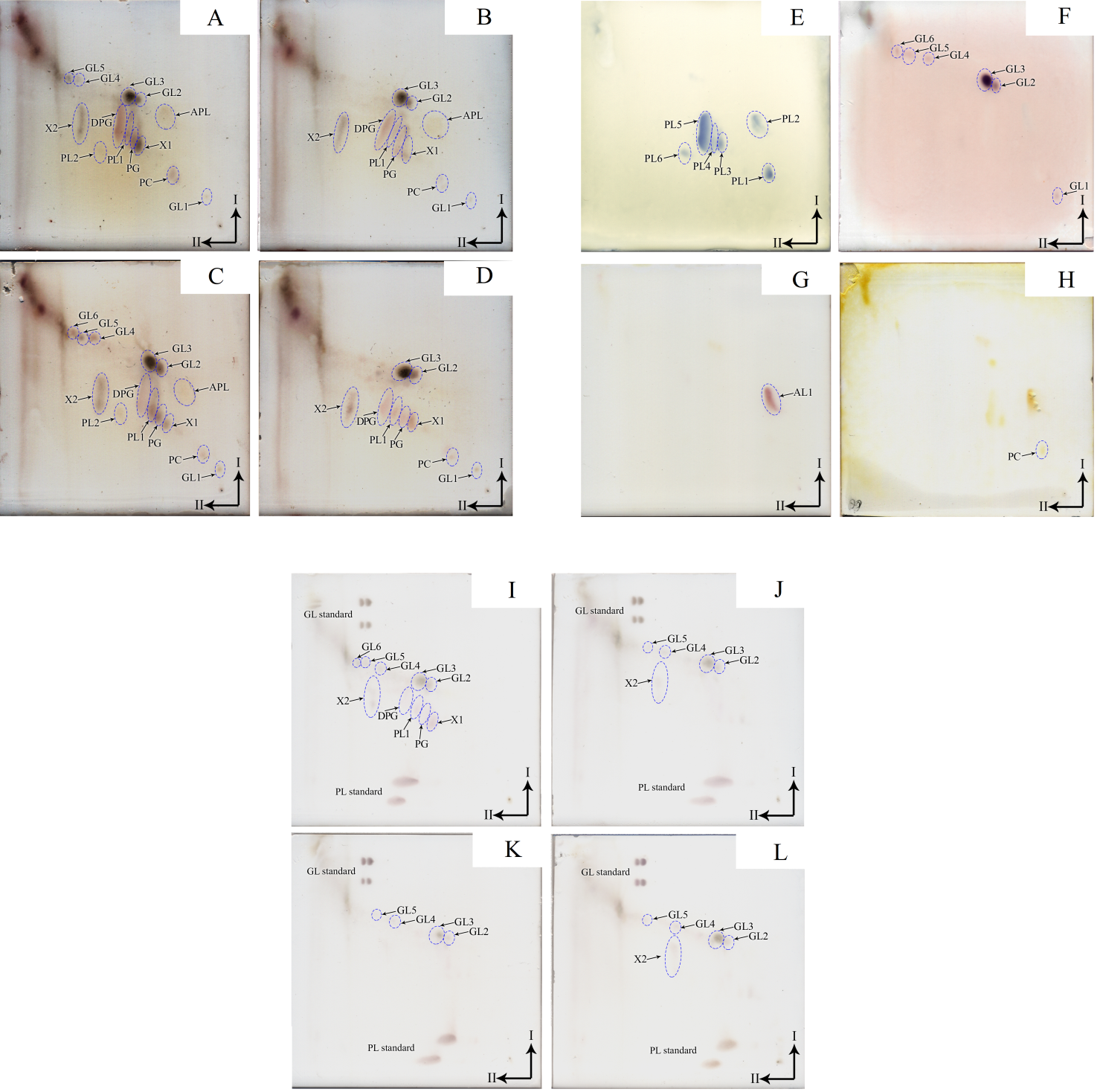


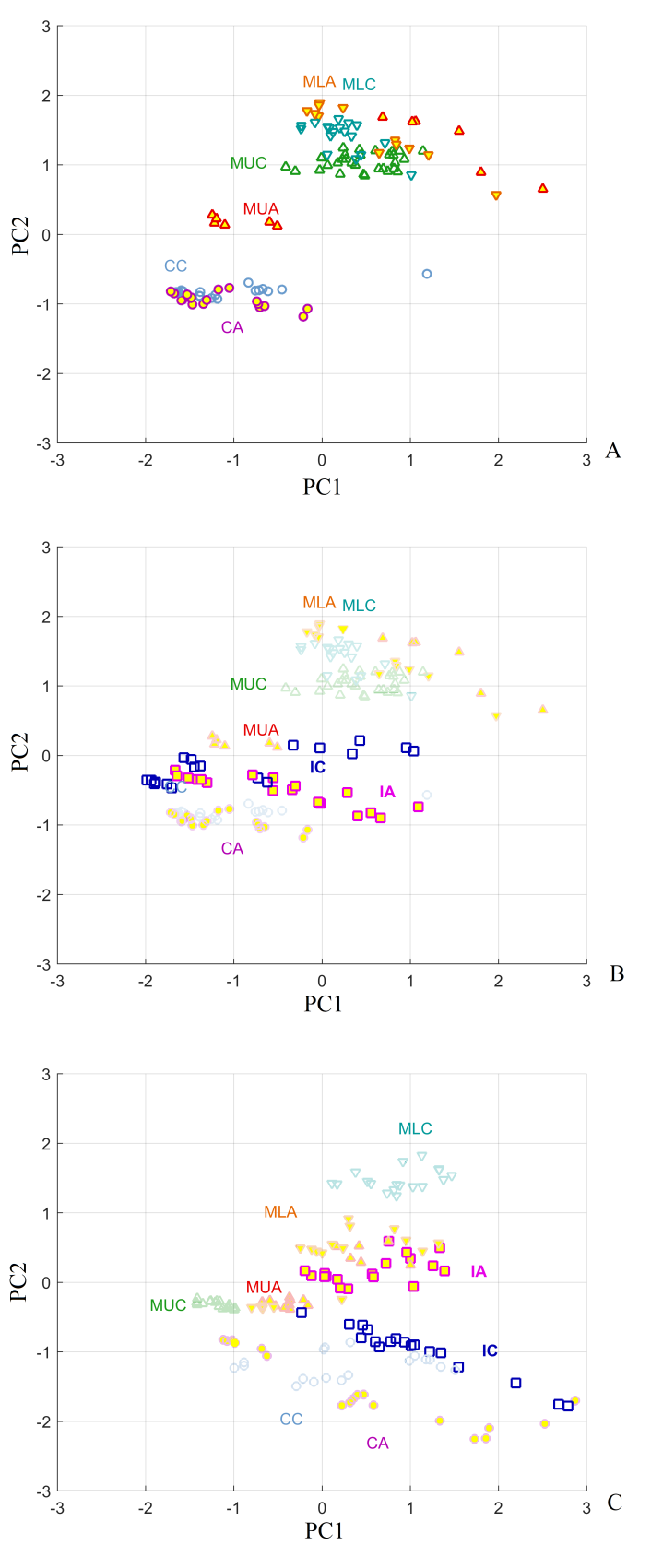


Supplementary Figure 5. Principal component analysis of SERS spectra for 72 h samples (CC – control cells, CA – epinephrine-treated cells, MUC – control upper phase of the matrix, MUA – epinephrine-treated upper phaseof the matrix, MLC


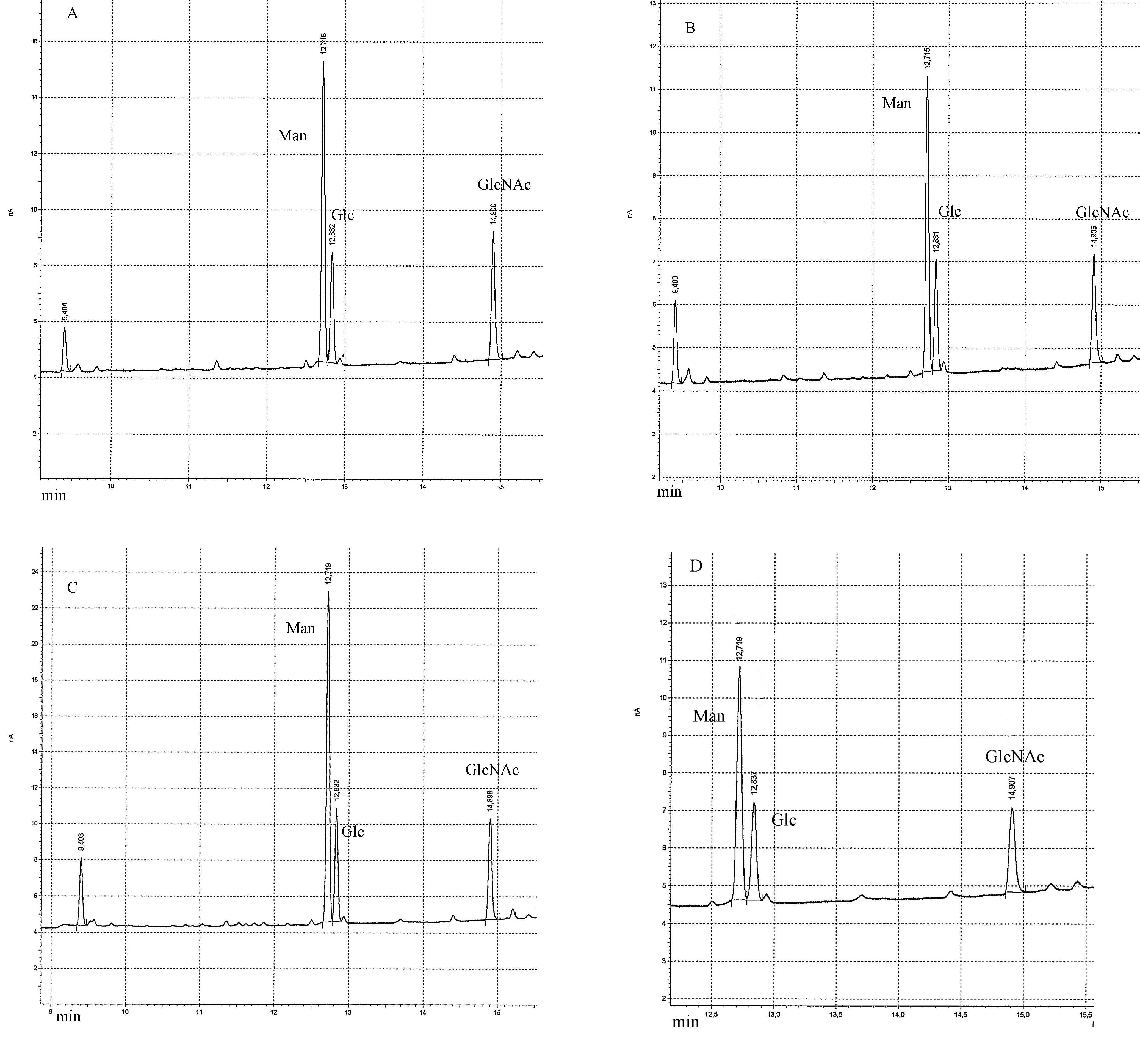


Supplementary Figure 6. GLC analysis of the alditol acetates derived from the matrix polysaccharides from *M. luteus* C01. Man, mannose; Glc, glucose; GlcNAc, 2-acetamido-2-deoxy-glucose. **A** - 24 h (control); **B** – 24 h (epinephrine), **C** – 72 h (control), **D** - 72 h (epinephrine).


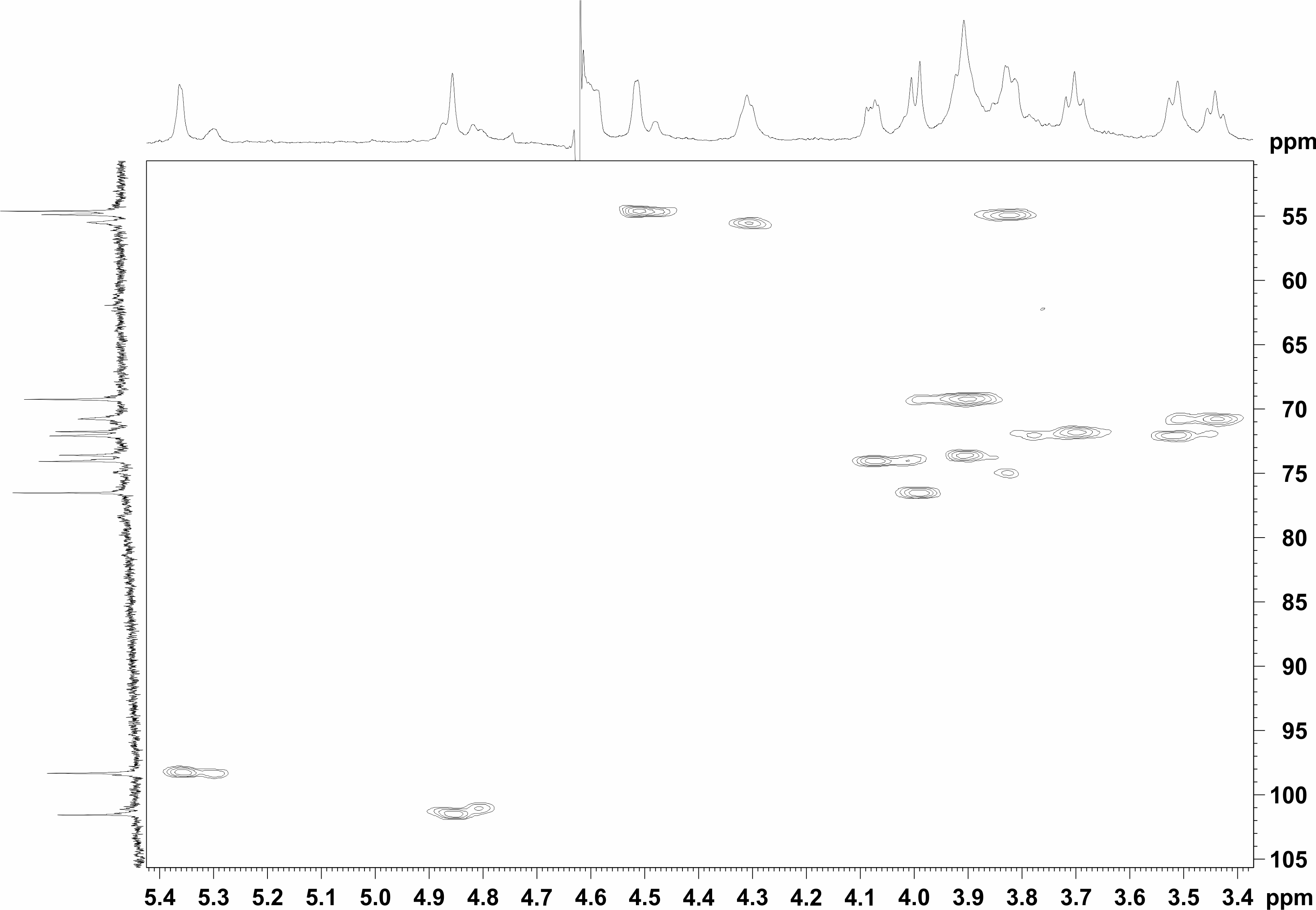


Supplementary Figure 7. Part of the 2D ^1^H,^13^C edHSQC spectrum of *M. luteus* C01 matrix polysaccharides (24 h (control)) eluted in 0.5M phosphate buffer. The corresponding parts of the ^1^H and ^13^C NMR spectra are shown along the horizontal and vertical axes, respectively.


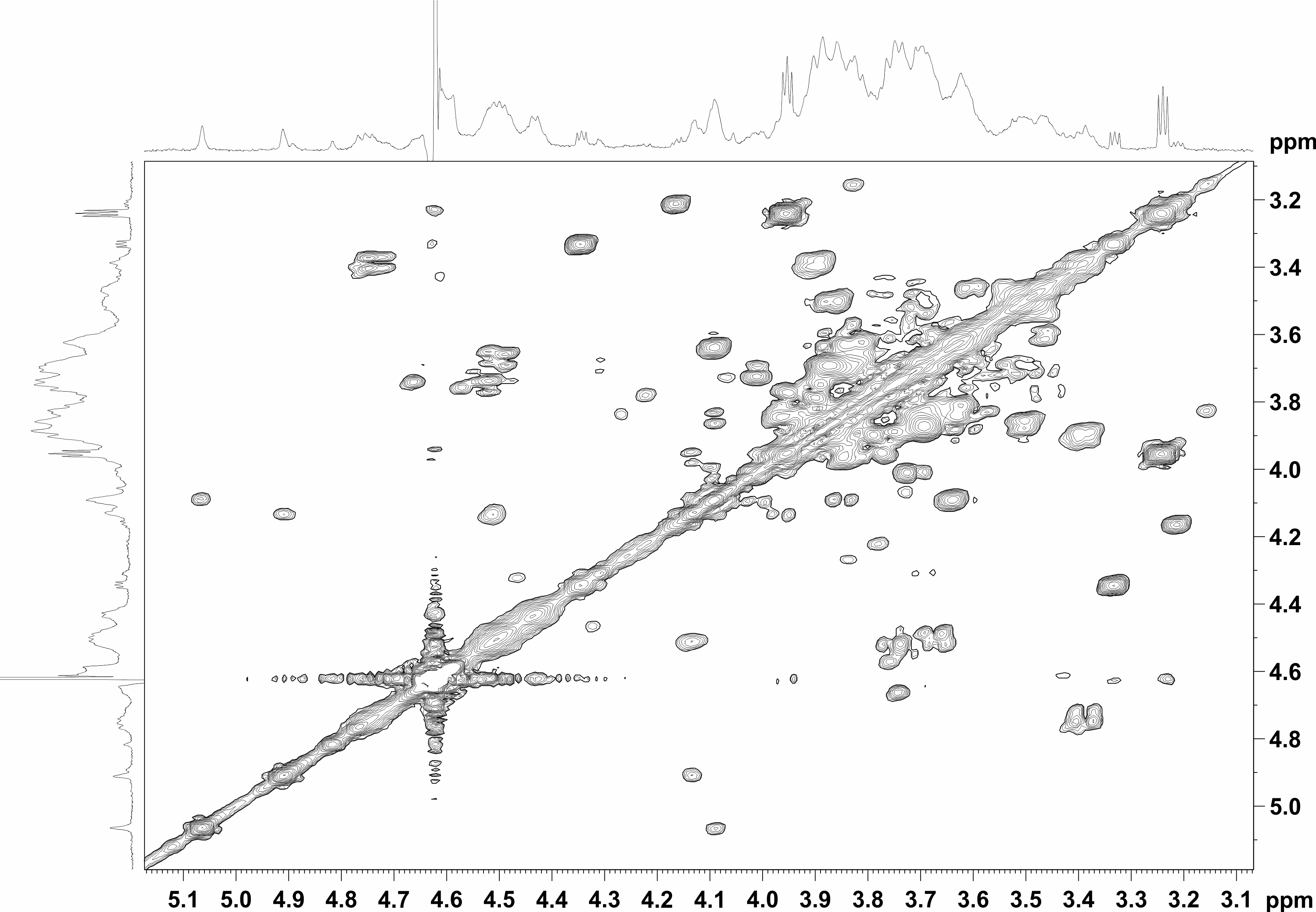


Supplementary Figure 8. Part of the 2D ^1^H,^1^H COSY spectrum of *M. luteus* C01 matrix polysaccharides (24 h (control)) eluted in 0.1M phosphate buffer. The corresponding parts of the ^1^H NMR spectra are shown along the horizontal and vertical axes.


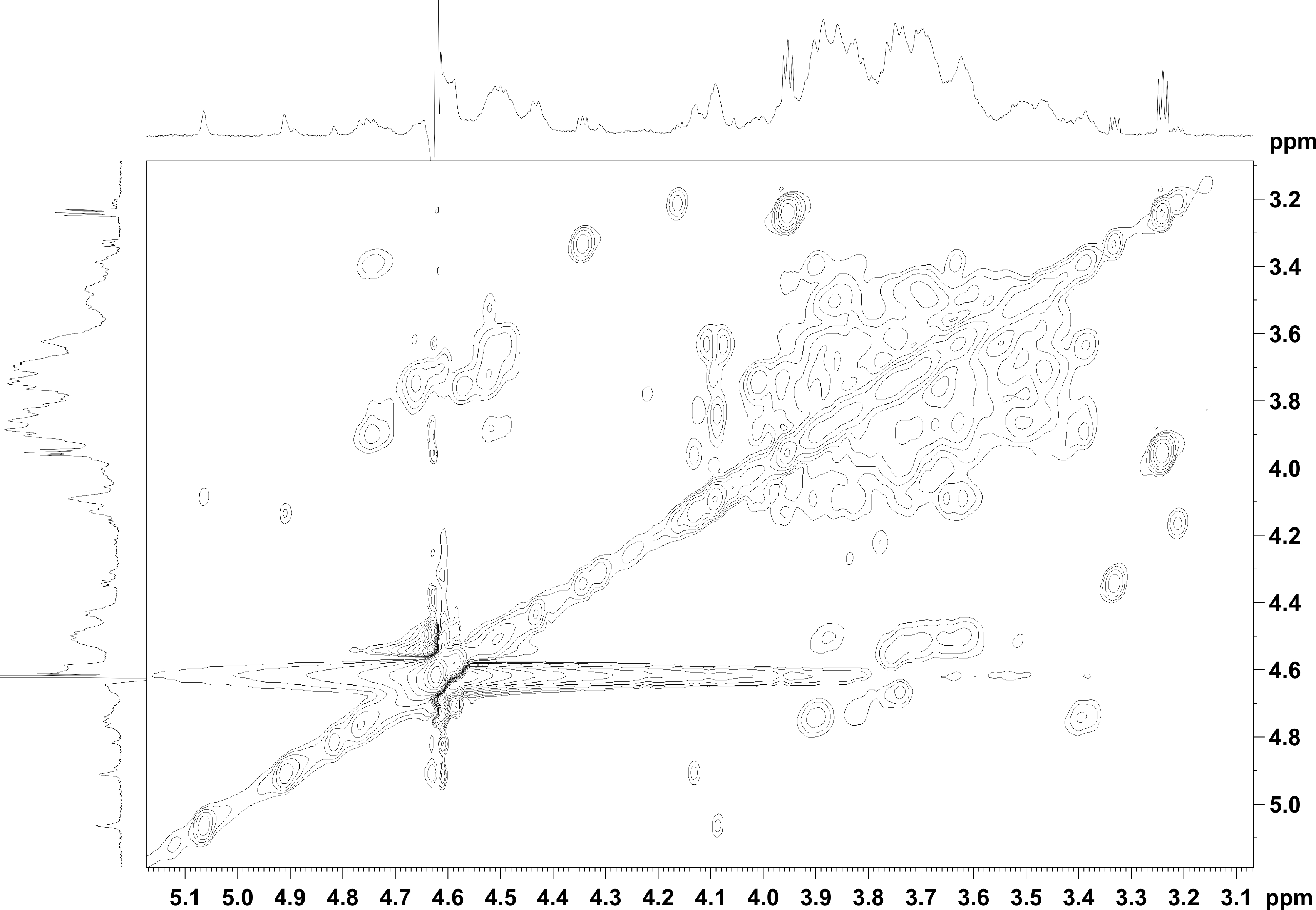


Supplementary Figure 9. Part of the 2D ^1^H,^1^H TOCSY spectrum of *M. luteus* C01 matrix polysaccharides (24 h (control)) eluted in 0.1M phosphate buffer. The corresponding parts of the ^1^H NMR spectra are shown along the horizontal and vertical axes.


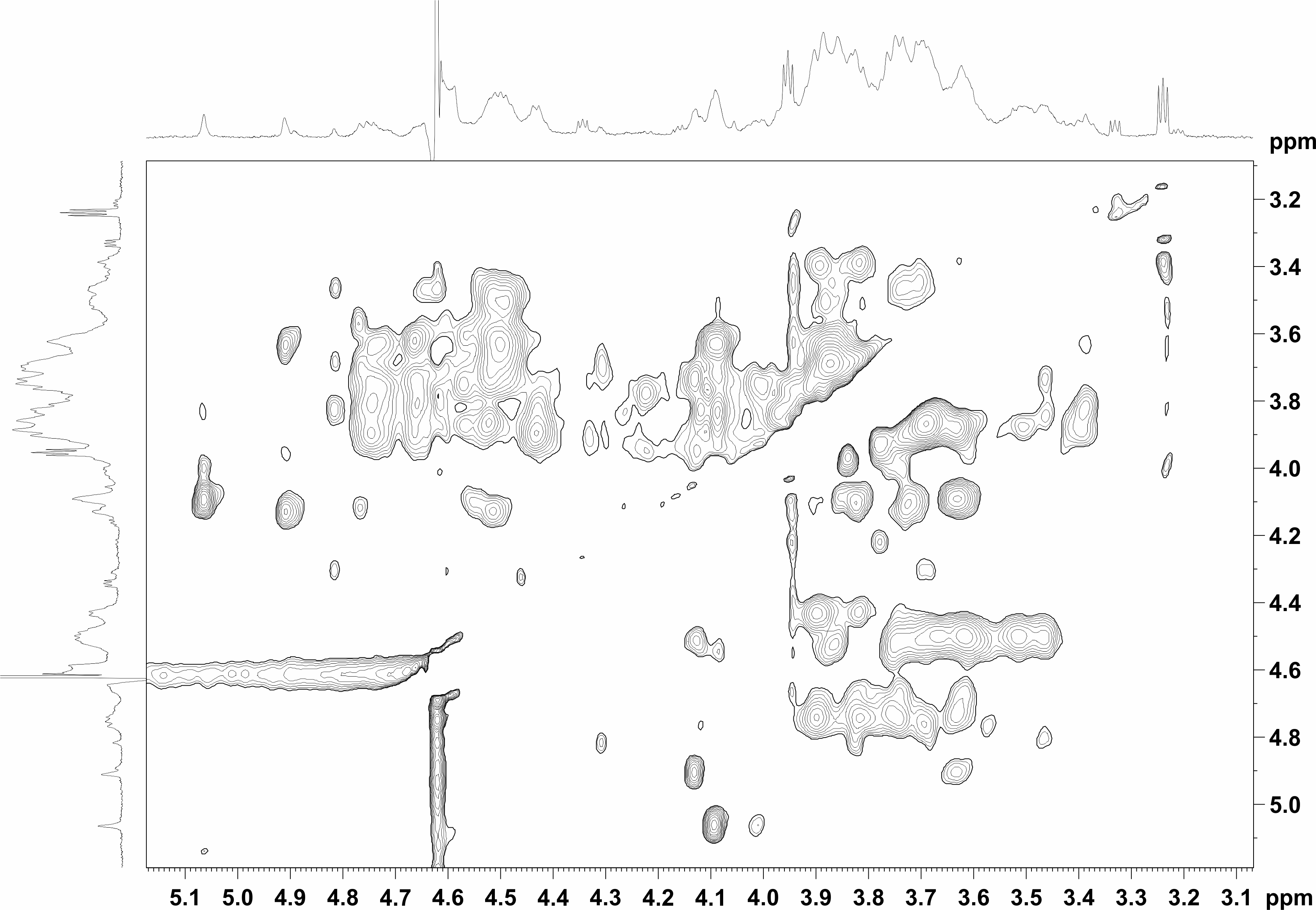


Supplementary Figure 10. Part of the 2D ^1^H,^1^H ROESY spectrum of *M. luteus* C01 matrix polysaccharides (24 h (control)) eluted in 0.1M phosphate buffer. The corresponding parts of the ^1^H NMR spectra are shown along the horizontal and vertical axes.


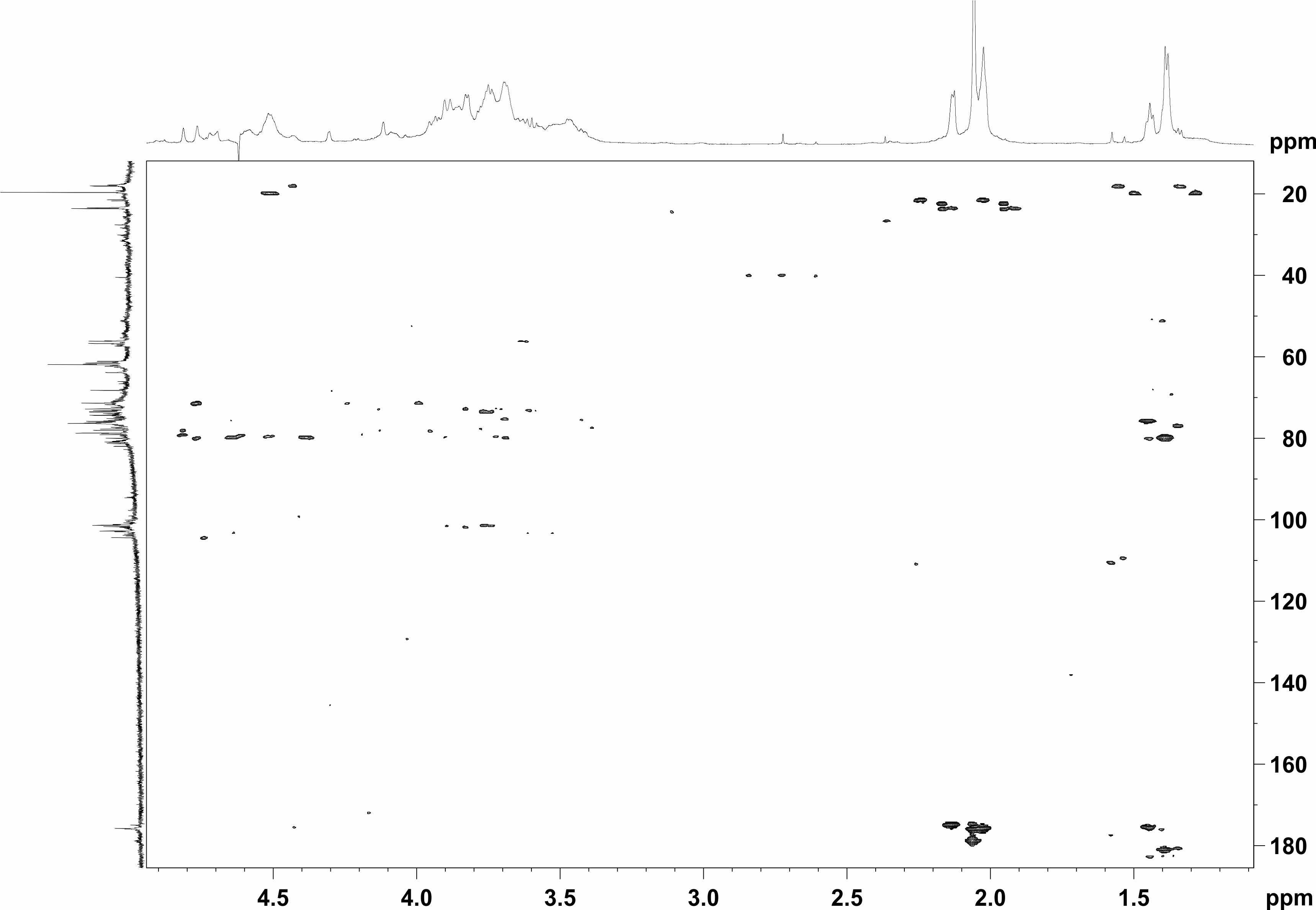


Supplementary Figure 11. Part of the 2D ^1^H,^13^C HMBC spectrum of *M. luteus* C01 matrix polysaccharides (24 h (control)) eluted in 0.1M phosphate buffer. The corresponding parts of the ^1^H and ^13^C NMR spectra are shown along the horizontal and vertical axes, respectively.


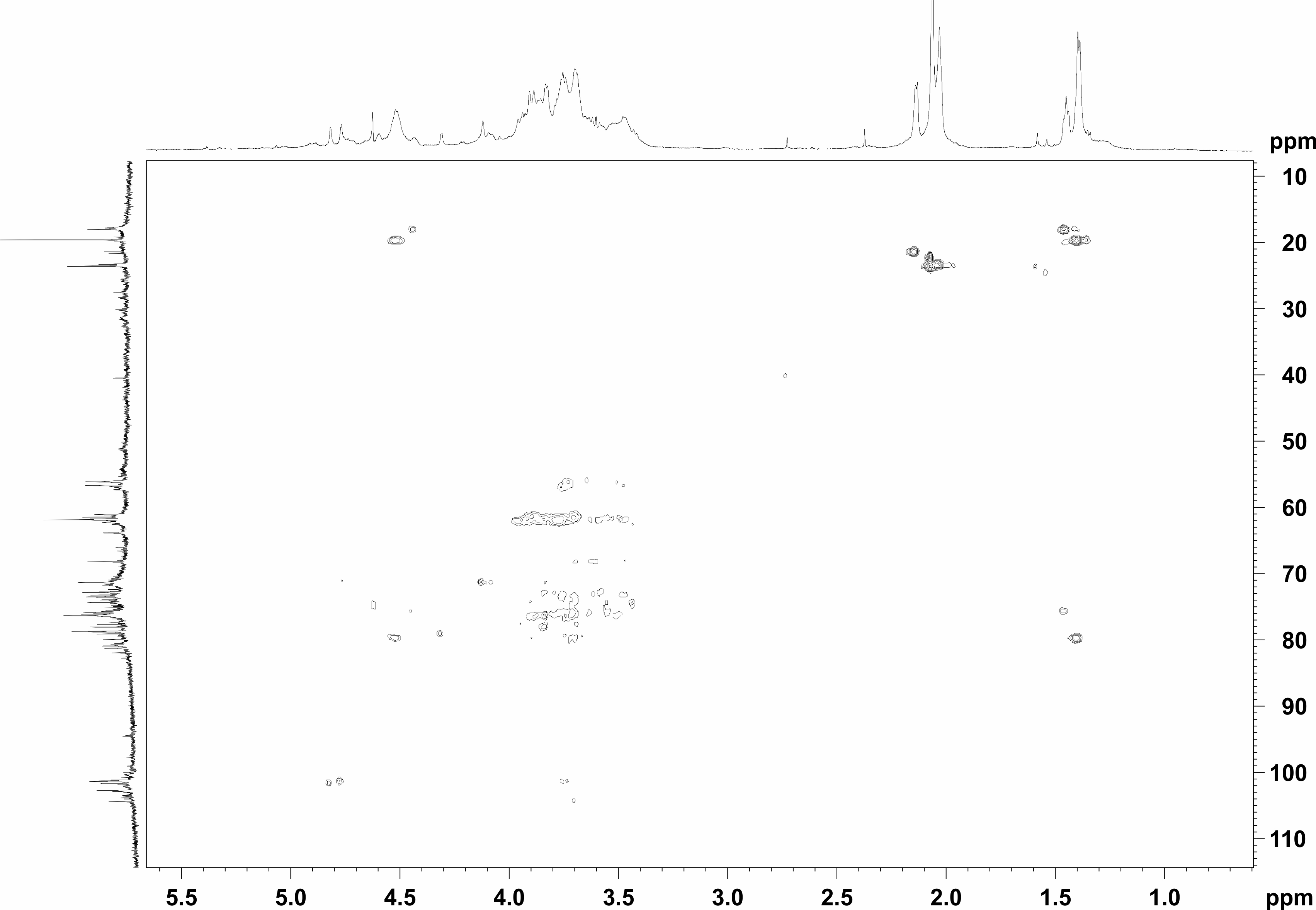


Supplementary Figure 12. Part of the 2D ^1^H,^13^C HSQC-TOCSY spectrum of *M. luteus* C01 matrix polysaccharides (24 h (control)) eluted in 0.1M phosphate buffer. The corresponding parts of the ^1^H and ^13^C NMR spectra are shown along the horizontal and vertical axes, respectively.


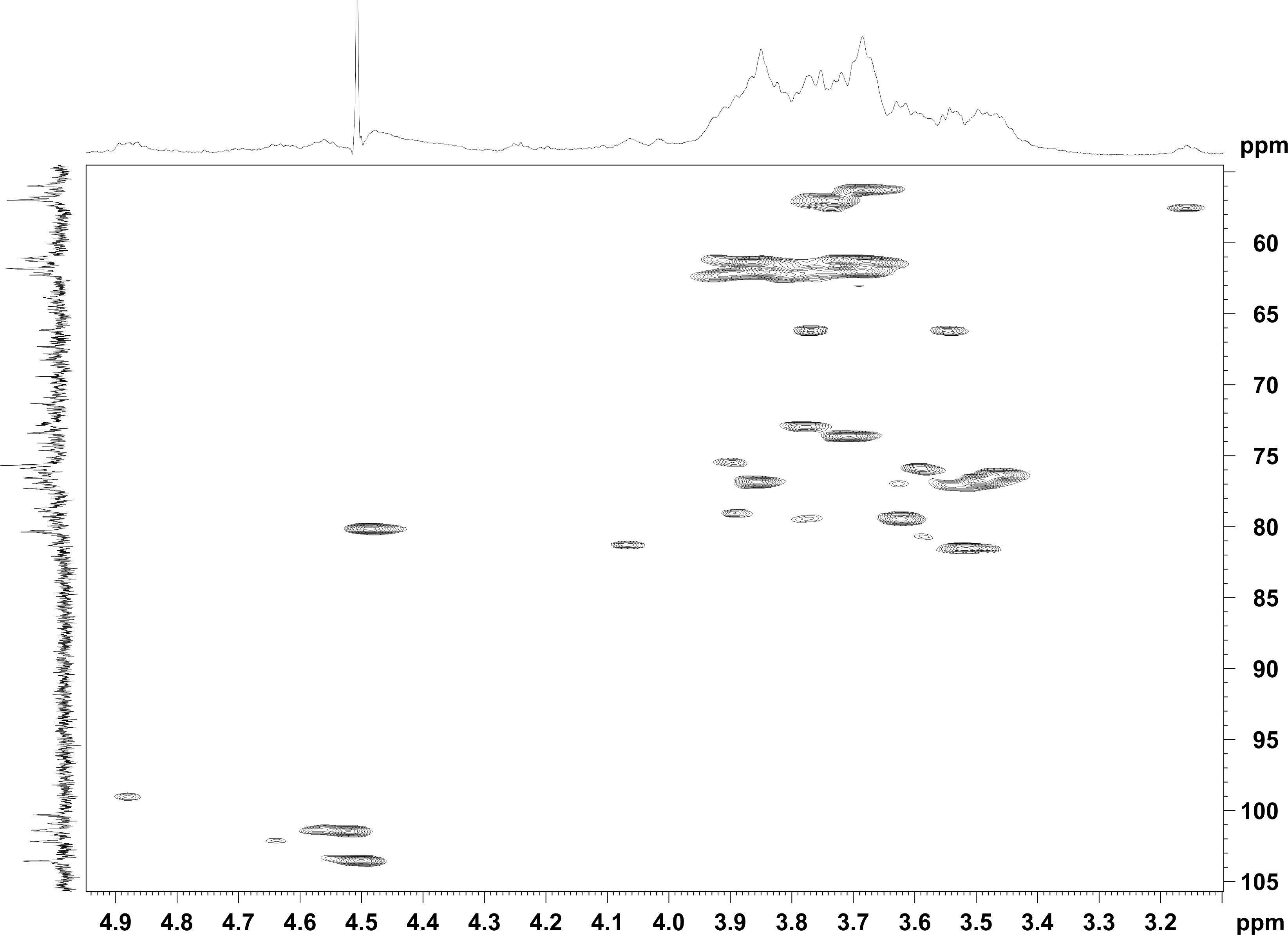


Supplementary Figure 13. Part of the 2D ^1^H,^13^C edHSQC spectrum of *M. luteus* C01 matrix polysaccharides after Smith degradation (24 h (control)). The corresponding parts of the ^1^H and ^13^C NMR spectra are shown along the horizontal and vertical axes, respectively.


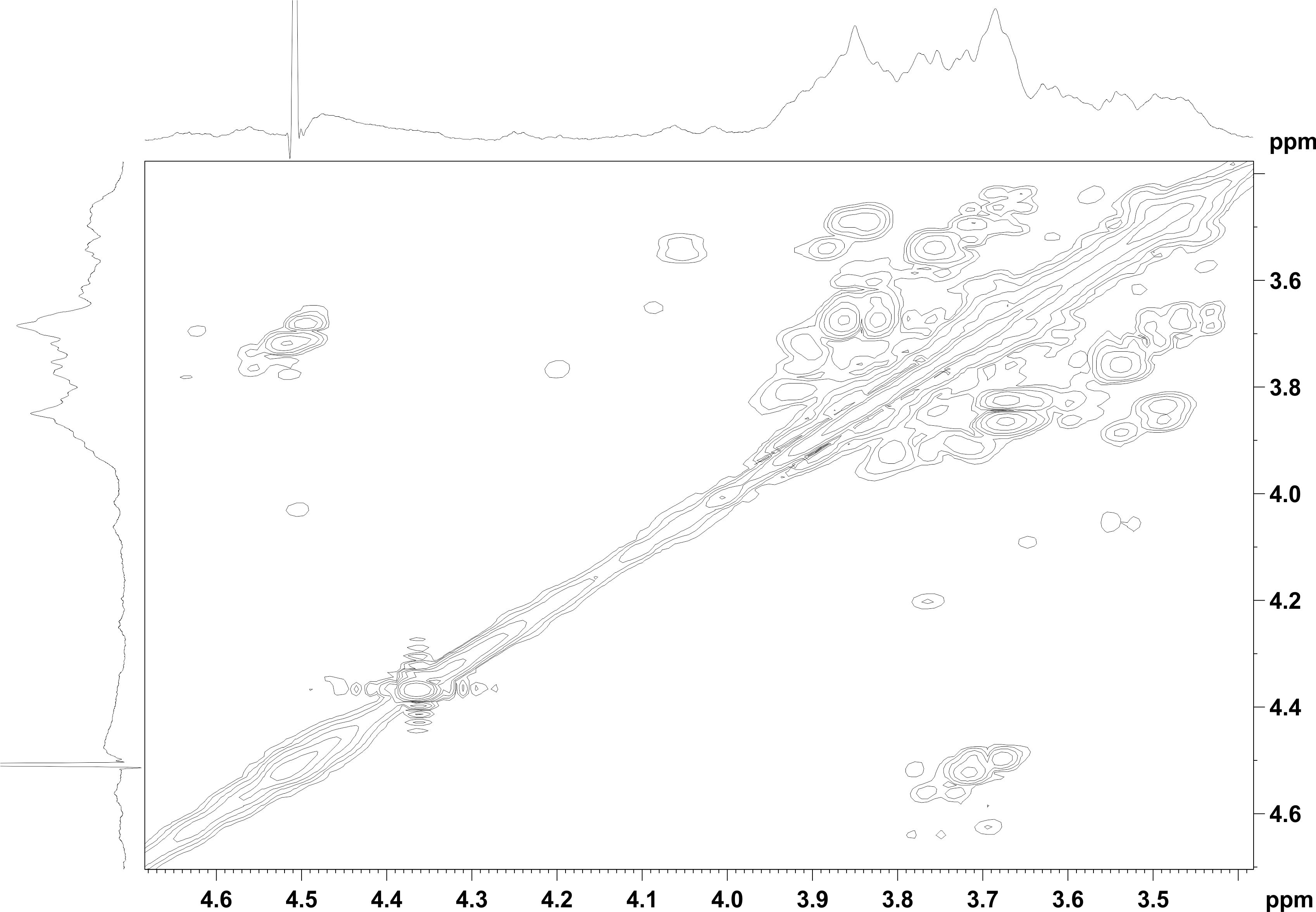


Supplementary Figure 14. Part of the 2D ^1^H,^1^H COSY spectrum of *M. luteus* C01 matrix polysaccharides after Smith degradation (24 h (control)). The corresponding parts of the ^1^H NMR spectra are shown along the horizontal and vertical axes.


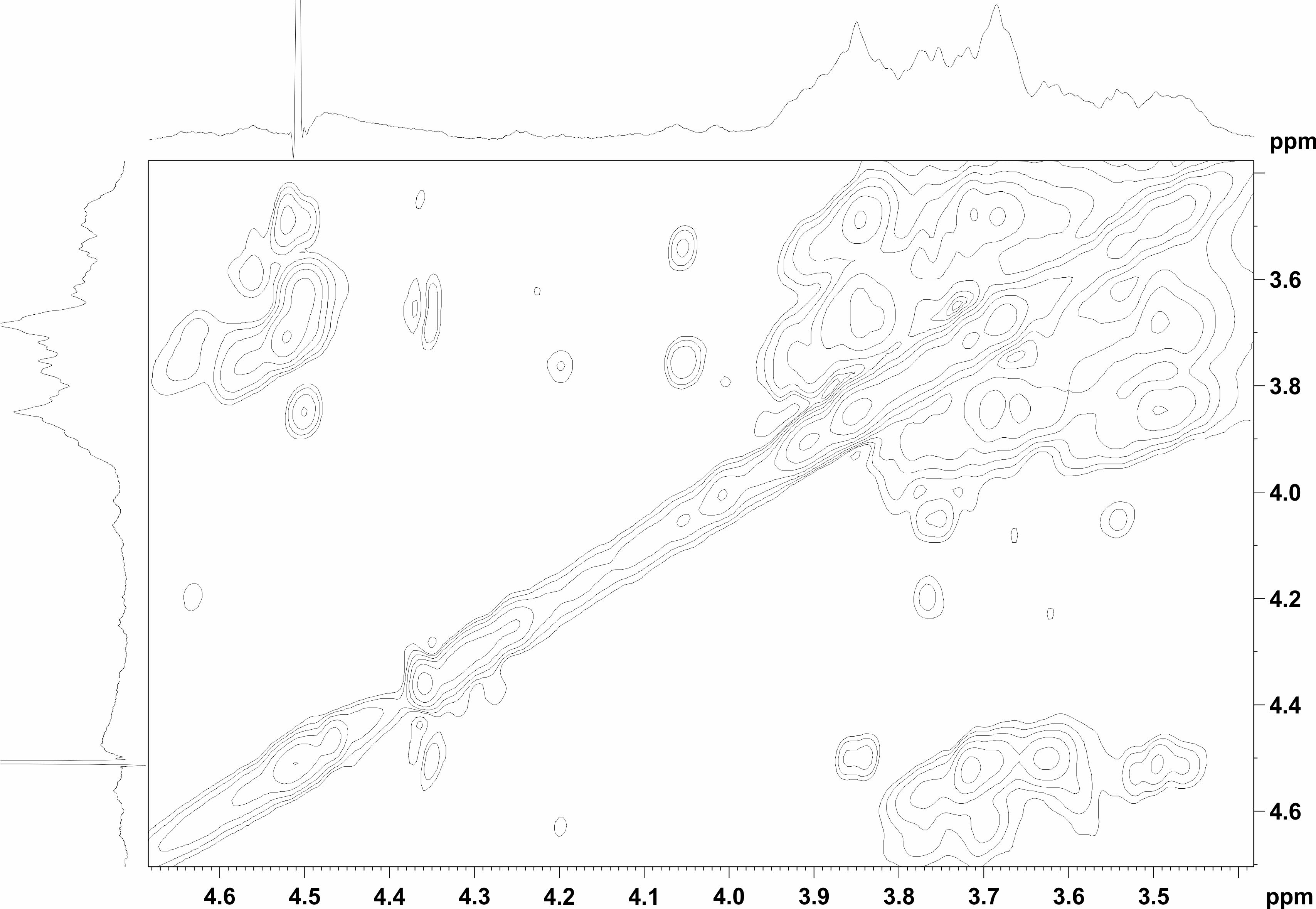


Supplementary Figure 15. Part of the 2D ^1^H,^1^H TOCSY spectrum of *M. luteus* C01 matrix polysaccharides after Smith degradation (24 h (control)). The corresponding parts of the ^1^H NMR spectra are shown along the horizontal and vertical axes.


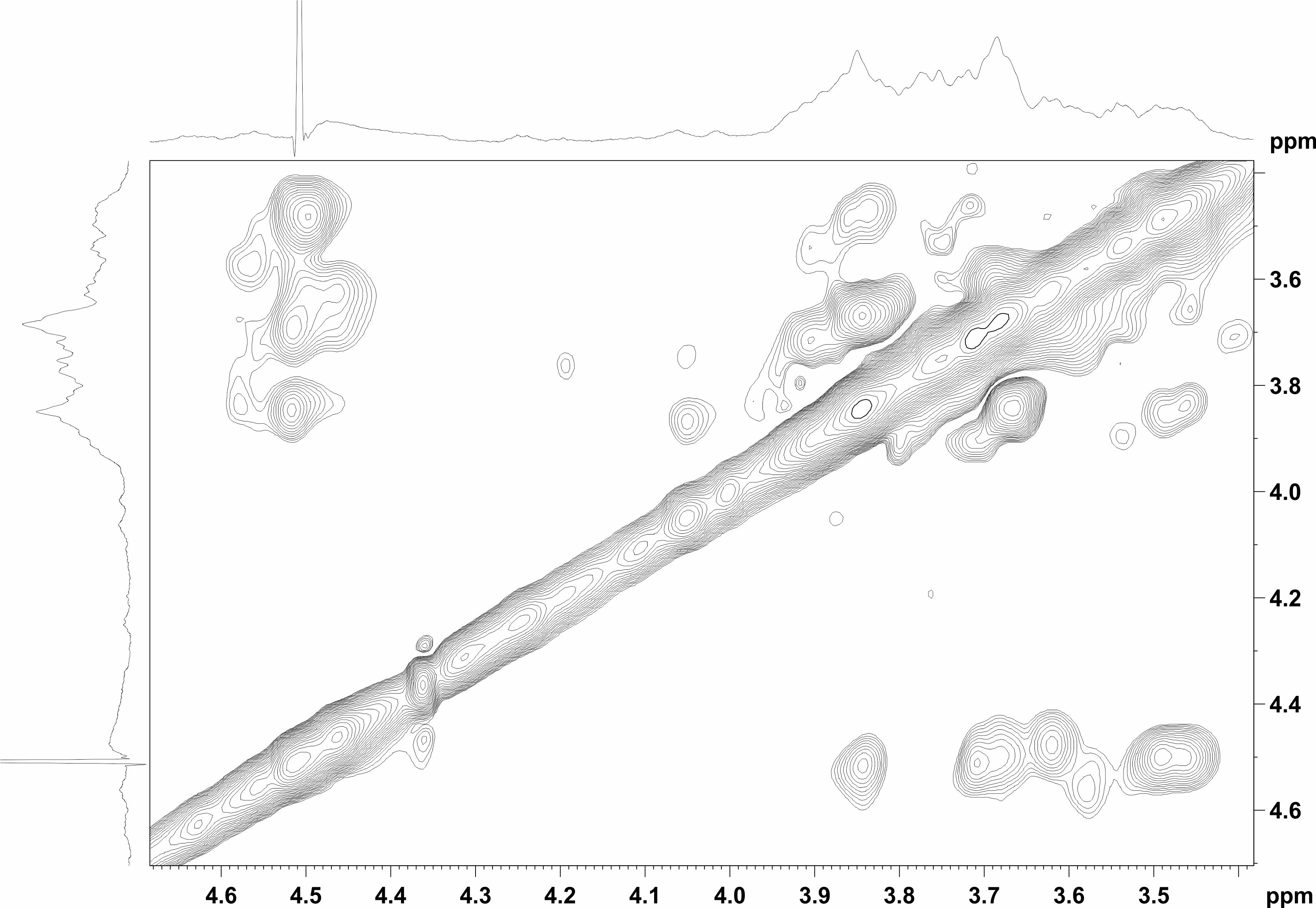


Supplementary Figure 16. Part of the 2D ^1^H,^1^H ROESY spectrum of *M. luteus* C01 matrix polysaccharides after Smith degradation (24 h (control)). The corresponding parts of the ^1^H NMR spectra are shown along the horizontal and vertical axes.


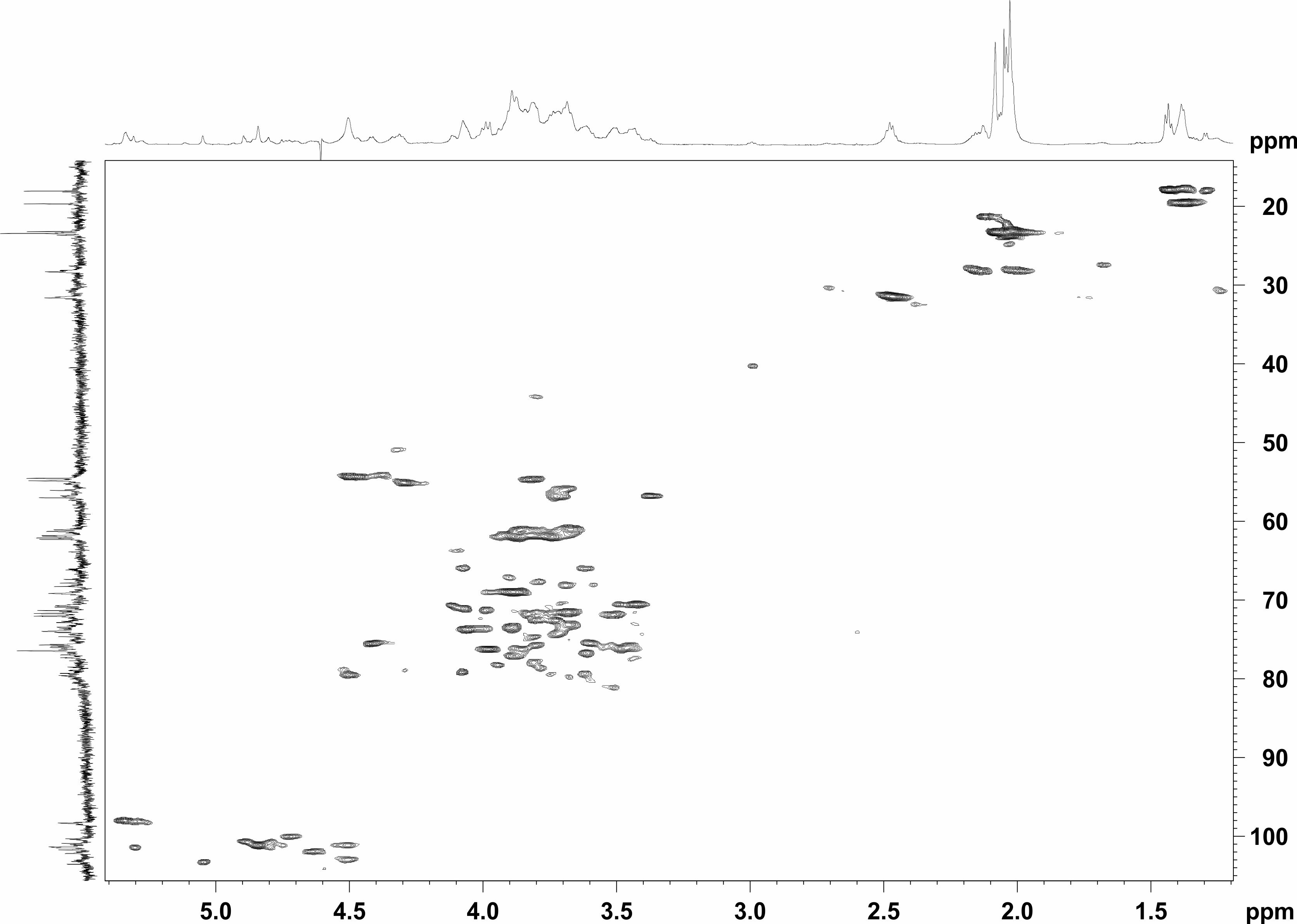


Supplementary Figure 17. Part of the 2D ^1^H,^13^C edHSQC spectrum of *M. luteus* C01 native matrix polysaccharides (24 h (control)). The corresponding parts of the ^1^H and ^13^C NMR spectra are shown along the horizontal and vertical axes, respectively.


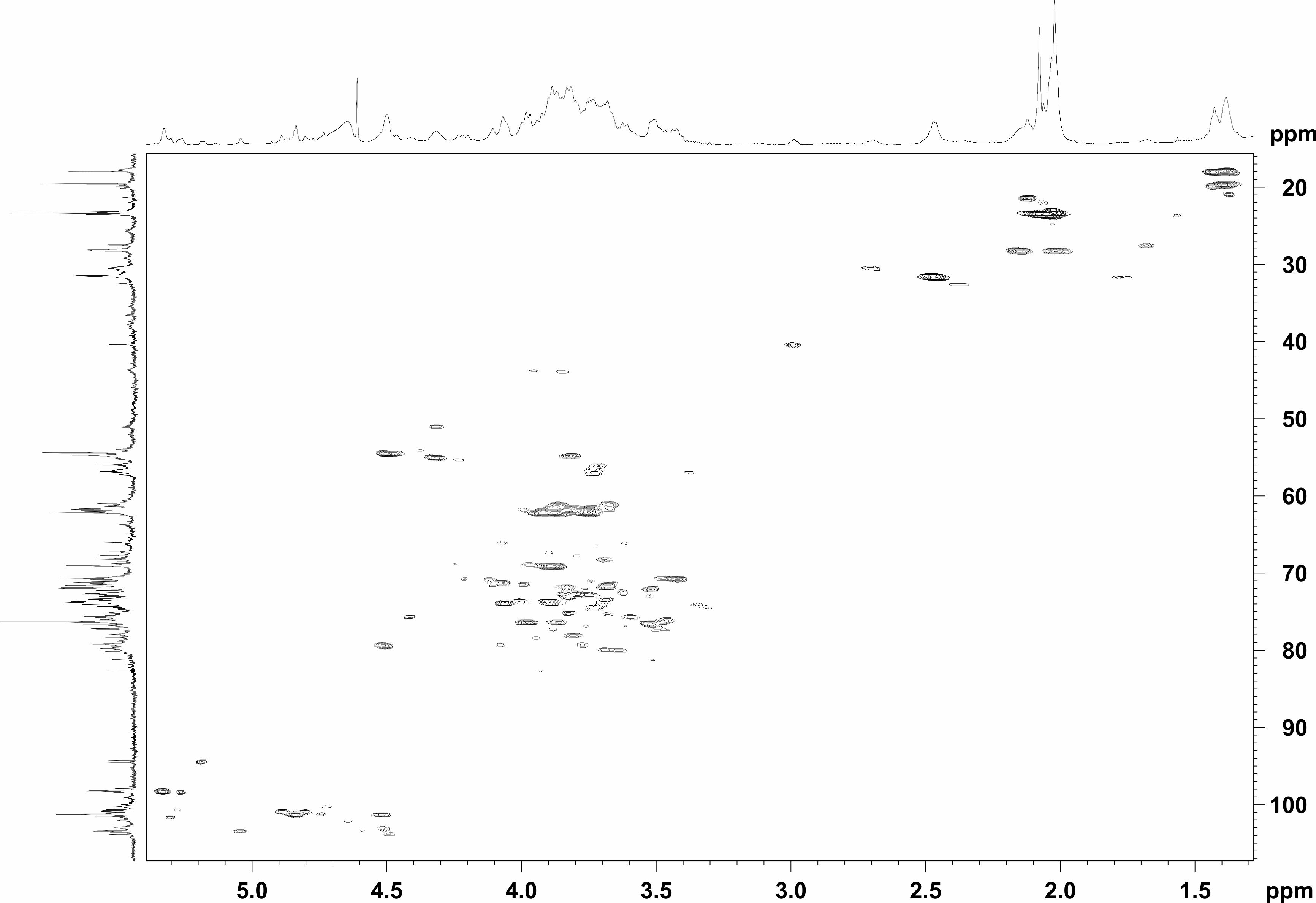


Supplementary Figure 18. Part of the 2D ^1^H,^13^C edHSQC spectrum of *M. luteus* C01 native matrix polysaccharides (24 h (epinephrine)). The corresponding parts of the ^1^H and ^13^C NMR spectra are shown along the horizontal and vertical axes, respectively.


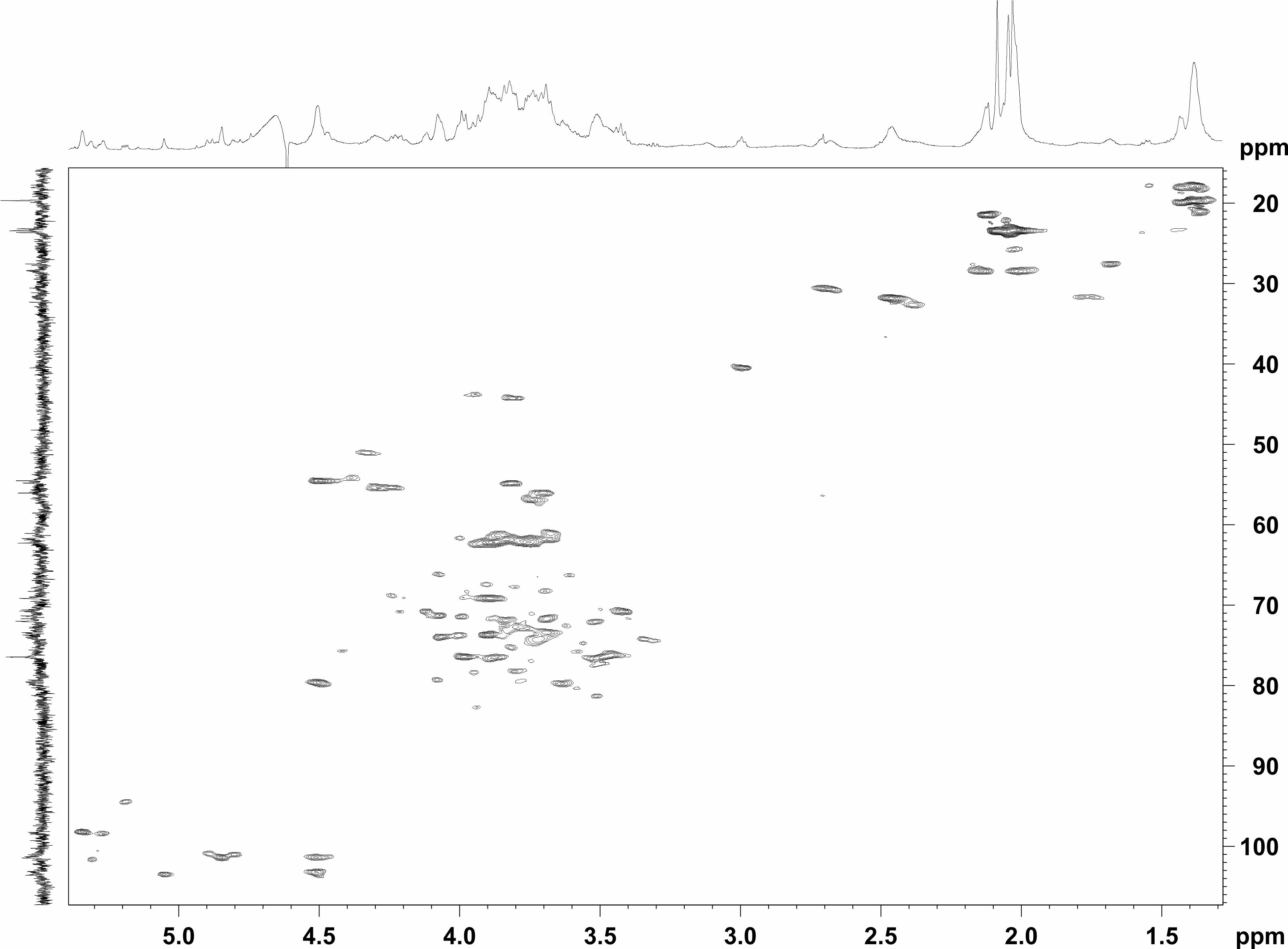


Supplementary Figure 19. Part of the 2D ^1^H,^13^C edHSQC spectrum of *M. luteus* C01 native matrix polysaccharides (72 h (control)). The corresponding parts of the ^1^H and ^13^C NMR spectra are shown along the horizontal and vertical axes, respectively.


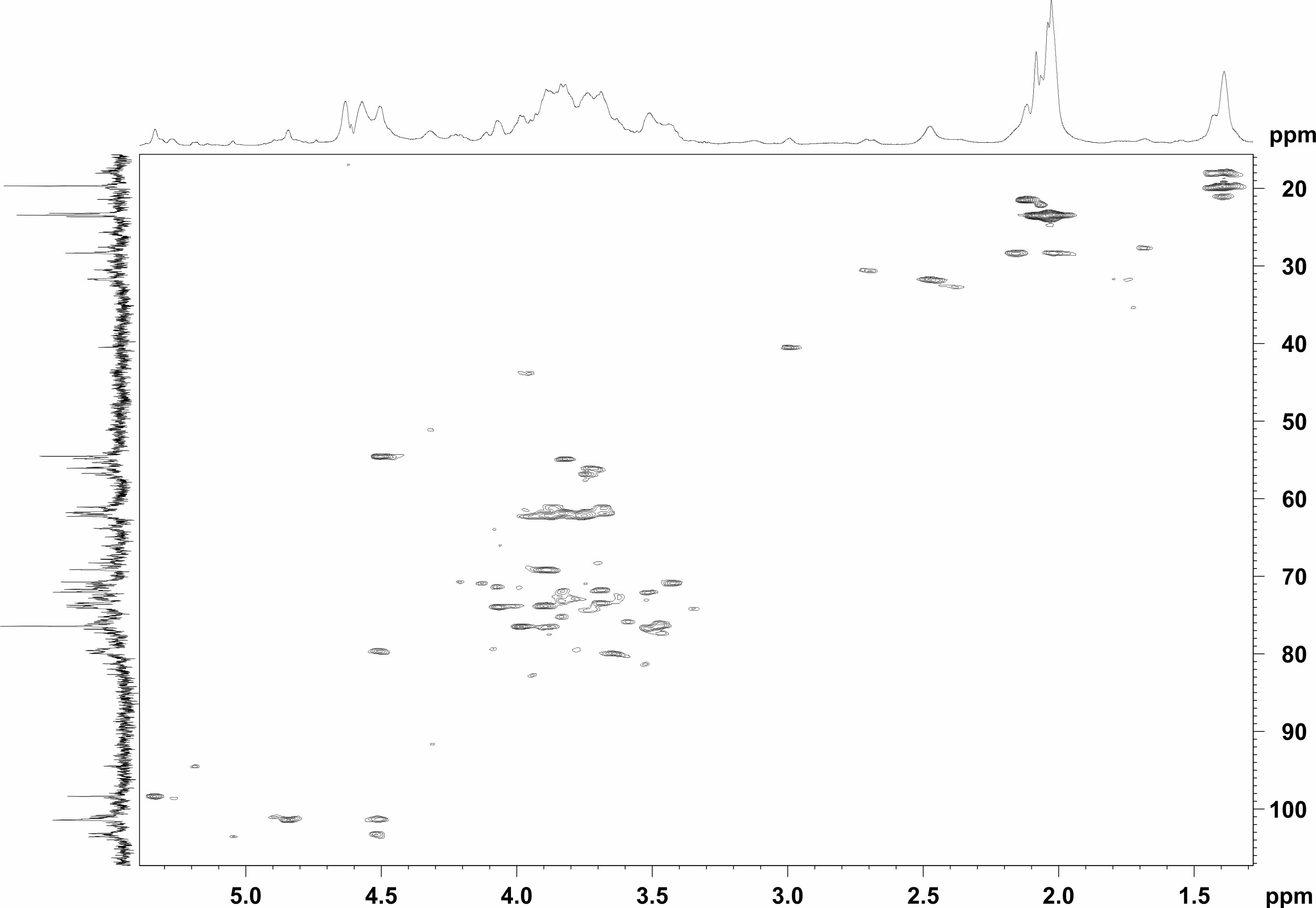


Supplementary Figure 20. Part of the 2D ^1^H,^13^C edHSQC spectrum of *M. luteus* C01 native matrix polysaccharides (72 h (epinephrine)). The corresponding parts of the ^1^H and ^13^C NMR spectra are shown along the horizontal and vertical axes, respectively.
