# Supplementary material for: Epinephrine extensively changes the biofilm matrix composition in Micrococcus luteus C01 isolated from human skin
Source: Front Microbiol. 2022 Sep 20;13:1003942. doi: 10.3389/fmicb.2022.1003942 (PMC9530943; doi:10.3389/fmicb.2022.1003942)
Supplement: Supplementary file 2 [file Table_1.DOCX]

Supplementary table S1. The most abundant proteins in the biofilm matrix of *M. luteus* C01.

| Accession | Protein name | Peptides | Unique peptides | Sequence coverage [%] | Unique sequence coverage [%] | Mol. weight [kDa] | Score | Intensity | iBAQ peptides | MS/MS count |
| --- | --- | --- | --- | --- | --- | --- | --- | --- | --- | --- |
| A0A653PUG0 | Chlorite O(2)-lyase | 12 | 11 | 59,7 | 55 | 27,175 | 209,19 | 21701000000 | 13 | 115 |
| A0A653PZD2 | ABC transporter permease | 12 | 12 | 34,3 | 34,3 | 42,086 | 189,1 | 9223400000 | 17 | 115 |
| A0A5E8QEK0 | Peroxidase | 8 | 8 | 16,3 | 16,3 | 50,252 | 135,92 | 8191400000 | 20 | 115 |
| C5CCN1 | Uncharacterized protein | 15 | 15 | 50,7 | 50,7 | 29,125 | 247,27 | 14400000000 | 12 | 116 |
| A0A5F0I7W1 | DNA translocase FtsK | 24 | 2 | 31,3 | 6,3 | 110,88 | 323,31 | 3644300000 | 43 | 118 |
| A0A2N6RT58 | Aldehyde dehydrogenase | 17 | 1 | 43,3 | 8,4 | 53,525 | 220,49 | 4754000000 | 23 | 119 |
| A0A031IFF2 | Foldase YidC | 7 | 4 | 25,9 | 15,7 | 36,847 | 89,276 | 15246000000 | 11 | 119 |
| A0A031HG31 | Na+/H+ antiporter subunit G | 6 | 6 | 36 | 36 | 18,738 | 159,36 | 3628500000 | 8 | 122 |
| C5CBV8 | Undecaprenyl-phosphate mannosyltransferase | 11 | 11 | 56,4 | 56,4 | 26,764 | 178,79 | 3426600000 | 13 | 124 |
| C5C8M8 | ^[[1]](#footnote-1)^Uncharacterized protein^1^ | 7 | 1 | 16,5 | 2,6 | 31,71 | 128,64 | 4592300000 | 8 | 124 |
| A0A4Y9HBN9 | Thiol reductant ABC exporter subunit CydC | 21 | 9 | 20,9 | 10,5 | 120,44 | 204,59 | 8219600000 | 52 | 125 |
| A0A5F0UQM0 | Lactate utilization protein C | 8 | 6 | 50,6 | 43,3 | 25,071 | 181,52 | 3401600000 | 10 | 126 |
| A0A378NNE4 | Daunorubicin/doxorubicin resistance ATP-binding protein DrrA | 12 | 0 | 56,9 | 0 | 29,108 | 160 | 13757000000 | 15 | 127 |
| A0A4U1LBU8 | Polysaccharide biosynthesis tyrosine autokinase | 10 | 1 | 23,8 | 3,4 | 49,028 | 153,53 | 13247000000 | 20 | 127 |
| A0A4U1MFH2 | DUF2662 domain-containing protein | 12 | 0 | 48,9 | 0 | 31,586 | 218,43 | 6133600000 | 15 | 128 |
| A0A031GV11 | Cation/acetate symporter ActP | 7 | 7 | 12,4 | 12,4 | 56,011 | 116,85 | 18194000000 | 11 | 128 |
| A0A031IFH7 | Cell division protein SepF | 10 | 10 | 63,3 | 63,3 | 22,208 | 135,3 | 6543900000 | 12 | 129 |
| A0A132I1X4 | DUF445 domain-containing protein | 18 | 15 | 52,9 | 44,9 | 46,788 | 276,42 | 10735000000 | 20 | 130 |
| A0A653IT60 | DUF4352 domain-containing protein | 4 | 3 | 17,8 | 9,6 | 21,342 | 97,376 | 14145000000 | 3 | 131 |
| A0A2N6RSH2 | NDP-hexose 4-ketoreductase | 31 | 29 | 40,5 | 38,2 | 93,374 | 323,31 | 4423800000 | 44 | 137 |
| A0A031IAZ7 | Cytochrome aa3 subunit 2 | 8 | 2 | 35,7 | 11,9 | 32,348 | 309,71 | 12228000000 | 12 | 137 |
| A0A5F0I888 | ABC transporter | 23 | 6 | 44 | 9 | 64,231 | 297,76 | 4199800000 | 30 | 138 |
| A0A031HM15 | Elongation factor Tu | 16 | 16 | 54,5 | 54,5 | 43,785 | 323,31 | 4915700000 | 20 | 140 |
| A0A1M7AJU5 | Methylmalonate-semialdehyde dehydrogenase [acylating] | 15 | 13 | 45,7 | 40 | 53,7 | 197,58 | 6206600000 | 24 | 140 |
| C5CBT1 | 3-ketoacyl-CoA thiolase | 16 | 3 | 46,1 | 9,2 | 46,53 | 235,67 | 9999900000 | 18 | 147 |
| A0A6N4C379 | ^[[2]](#footnote-2)^Uncharacterized protein^2^ | 13 | 13 | 51,8 | 51,8 | 20,817 | 216,24 | 27175000000 | 8 | 147 |
| A0A031GIZ8 | Thioesterase family protein | 13 | 13 | 63,2 | 63,2 | 22,22 | 164,44 | 12307000000 | 13 | 147 |
| A0A4Y8PJW7 | Iron-sulfur cluster-binding protein | 16 | 16 | 29,5 | 29,5 | 59,017 | 229,75 | 7626400000 | 29 | 148 |
| A0A509Y3B2 | HlyC/CorC family transporter | 17 | 17 | 41 | 41 | 48,762 | 198,1 | 9588100000 | 18 | 148 |
| A0A1M7BLI6 | Putative copper resistance protein D | 15 | 15 | 16,5 | 16,5 | 80,708 | 224,69 | 8315000000 | 25 | 150 |
| A0A5F0I760 | Peptidase aloeverae | 23 | 0 | 34,4 | 0 | 101,45 | 323,31 | 6544500000 | 29 | 151 |
| A0A653LY24 | ABC-type Fe3+ transport system, periplasmic component | 12 | 12 | 43 | 43 | 38,583 | 283,02 | 5482100000 | 10 | 151 |
| A0A509Y3R5 | NAD-dependent succinate-semialdehyde dehydrogenase | 20 | 20 | 69,8 | 69,8 | 49,102 | 323,31 | 11877000000 | 22 | 152 |
| A0A4Y8PHX6 | ^[[3]](#footnote-3)^Uncharacterized protein^3^ | 15 | 15 | 24,2 | 24,2 | 74,713 | 323,31 | 9181200000 | 17 | 153 |
| A0A031G1V8 | Succinate-semialdehyde dehydrogenase | 14 | 10 | 31,7 | 21 | 53 | 211,64 | 8864600000 | 23 | 155 |
| A0A653IPQ6 | Putative membrane protein | 20 | 20 | 40,6 | 40,6 | 60,233 | 253,39 | 7121400000 | 27 | 159 |
| A0A509Y5N1 | Na+/H+ antiporter subunit A | 12 | 12 | 10,9 | 10,9 | 110,2 | 190,92 | 10925000000 | 33 | 161 |
| A0A031IM96 | Phage holin family protein | 16 | 16 | 46,6 | 46,6 | 33,157 | 147,09 | 21641000000 | 14 | 162 |
| A0A1R4IGQ9 | ATP synthase subunit alpha | 21 | 2 | 35,7 | 3,9 | 59,291 | 160,7 | 13022000000 | 34 | 166 |
| A0A5F0I8U8 | ABC transporter ATP-binding protein | 11 | 11 | 48,6 | 48,6 | 27,061 | 234,73 | 14871000000 | 11 | 167 |
| A0A031GZP3 | GTPase Era | 27 | 3 | 53,1 | 9,4 | 61,616 | 323,31 | 8906000000 | 30 | 168 |
| A0A4Y8PKF3 | FMN-binding glutamate synthase family protein | 24 | 0 | 40,1 | 0 | 72,928 | 294,88 | 9040700000 | 32 | 169 |
| A0A4V5PZD3 | DUF1461 domain-containing protein | 12 | 0 | 43,8 | 0 | 43,464 | 323,31 | 10628000000 | 20 | 172 |
| A0A4U1MBF7 | Acyl-CoA dehydrogenase | 27 | 21 | 49,6 | 41,4 | 77,845 | 318,57 | 12353000000 | 39 | 174 |
| A0A5F0IBK0 | Glycine dehydrogenase (decarboxylating) | 28 | 0 | 39,5 | 0 | 104,08 | 323,31 | 10328000000 | 37 | 179 |
| A0A653IWV7 | Cytochrome C biogenesis protein ResB (Modular protein) | 16 | 12 | 18,4 | 15,1 | 103,41 | 323,31 | 7903300000 | 27 | 180 |
| A0A2I1XY38 | ^[[4]](#footnote-4)^Uncharacterized protein^4^ | 20 | 1 | 54,6 | 2,4 | 53,742 | 323,31 | 5708800000 | 33 | 183 |
| C5C9Q9 | ^[[5]](#footnote-5)^Uncharacterized protein^5^ | 10 | 9 | 69,8 | 66,3 | 21,705 | 323,31 | 12974000000 | 10 | 185 |
| A0A2N6RLF0 | Dephospho-CoA kinase | 17 | 17 | 25,3 | 25,3 | 85,336 | 277,69 | 7449000000 | 22 | 189 |
| A0A6N4C1A9 | Sodium:alanine symporter | 11 | 11 | 25,8 | 25,8 | 55,381 | 323,31 | 21501000000 | 9 | 191 |
| A0A562FVX3 | ^[[6]](#footnote-6)^Uncharacterized protein^6^ J28 | 7 | 3 | 45,5 | 19,8 | 19,498 | 201,72 | 68496000000 | 5 | 191 |
| A0A2N6RL67 | ^[[7]](#footnote-7)^Uncharacterized protein^7^ | 24 | 23 | 43,9 | 42,7 | 72,811 | 323,31 | 11078000000 | 26 | 194 |
| A0A5F0IAH8 | Iron-siderophore ABC transporter substrate-binding protein | 11 | 4 | 48 | 19,8 | 37,506 | 323,31 | 27137000000 | 13 | 199 |
| A0A562FW66 | Glutamate transport system ATP-binding protein | 16 | 2 | 55,5 | 7,7 | 30,071 | 323,31 | 21699000000 | 14 | 202 |
| A0A509Y333 | Glutamate ABC transporter substrate-binding protein | 11 | 3 | 46,8 | 16,8 | 31,074 | 323,31 | 29343000000 | 11 | 210 |
| A0A653IUF5 | Glyceraldehyde-3-phosphate dehydrogenase (NAD-dependent, glycolytic) | 15 | 15 | 50,3 | 50,3 | 36,614 | 268,52 | 30502000000 | 15 | 218 |
| A0A653PE11 | ABC-type multidrug transport system, ATPase component | 15 | 1 | 59,8 | 3,7 | 34,427 | 284,47 | 8220800000 | 21 | 219 |
| A0A1M7B968 | Cu2+-exporting ATPase | 19 | 1 | 40,5 | 5,1 | 74,404 | 320,95 | 7538800000 | 21 | 221 |
| A0A2N6RKZ3 | RNA polymerase subunit sigma-70 | 22 | 1 | 44,5 | 5,7 | 50,766 | 323,31 | 7043600000 | 18 | 231 |
| A0A1M7ASZ6 | Phosphate import ATP-binding protein PstB | 15 | 1 | 54,8 | 10 | 28,468 | 323,31 | 21217000000 | 14 | 234 |
| A0A031ISH3 | ABC transporter, ATP-binding protein | 21 | 21 | 44,7 | 44,7 | 56,721 | 304,17 | 18990000000 | 26 | 234 |
| A0A562FP89 | Signal recognition particle receptor FtsY | 16 | 12 | 39,6 | 30,6 | 42,334 | 258,58 | 37994000000 | 23 | 252 |
| D3LQK9 | ATP synthase subunit b | 12 | 4 | 59,2 | 13,6 | 19,758 | 297,88 | 55940000000 | 11 | 255 |
| A0A031H793 | bPH_2 domain-containing protein | 9 | 9 | 52,4 | 52,4 | 18,267 | 323,31 | 18568000000 | 8 | 259 |
| A0A653IQX5 | Protein translocase subunit SecA | 33 | 1 | 42,5 | 2,4 | 101 | 323,31 | 12111000000 | 51 | 262 |
| A0A2N6RI12 | 1-pyrroline-5-carboxylate dehydrogenase | 42 | 3 | 42,1 | 3,9 | 129,29 | 323,31 | 20025000000 | 73 | 267 |
| C5CCA8 | Cytochrome bc1 complex cytochrome b | 15 | 2 | 21,6 | 1,4 | 62,755 | 248,19 | 49234000000 | 20 | 267 |
| A0A031H2Y9 | Probable malate:quinone oxidoreductase | 19 | 7 | 52,6 | 19,8 | 53,187 | 323,31 | 22382000000 | 23 | 269 |
| A0A5F0I5R8 | Phosphate-binding protein PstS | 15 | 14 | 59,5 | 57,6 | 38,326 | 323,31 | 25536000000 | 12 | 280 |
| A0A5E8QA42 | ABC transporter ATP-binding protein | 24 | 1 | 52,9 | 1,5 | 62,606 | 316,33 | 14841000000 | 29 | 283 |
| A0A4Y8PLE4 | SSD domain-containing protein | 32 | 13 | 38,7 | 17,9 | 113,59 | 323,31 | 22101000000 | 41 | 293 |
| A0A562G510 | Carbon starvation protein | 14 | 3 | 18,7 | 5,9 | 79,422 | 323,31 | 21151000000 | 20 | 297 |
| A0A132HYV3 | S-(Hydroxymethyl)mycothiol dehydrogenase | 13 | 13 | 39,7 | 39,7 | 39,638 | 323,31 | 13554000000 | 18 | 312 |
| A0A2I1XY66 | Chaperone protein DnaK | 33 | 5 | 60,6 | 7,8 | 66,504 | 323,31 | 41529000000 | 31 | 318 |
| A0A1R4J414 | ATP synthase gamma chain | 17 | 17 | 53,2 | 53,2 | 32,65 | 323,31 | 38616000000 | 15 | 331 |
| A0A6N4FFH9 | TerC/Alx family metal homeostasis membrane protein | 16 | 0 | 24,8 | 0 | 46,387 | 262,92 | 41826000000 | 16 | 331 |
| D3LQK8 | ATP synthase subunit delta | 19 | 1 | 70,8 | 5,5 | 28,627 | 323,31 | 43819000000 | 16 | 332 |
| A0A653T9U7 | Serine/threonine-protein kinase | 19 | 2 | 34,3 | 7,8 | 70,324 | 323,31 | 17135000000 | 22 | 337 |
| A0A031IF52 | Succinate dehydrogenase iron-sulfur subunit | 21 | 21 | 76,4 | 76,4 | 29,554 | 323,31 | 46940000000 | 13 | 338 |
| A0A5E8QDN2 | SPFH/Band 7/PHB domain protein | 20 | 2 | 55,6 | 6,1 | 42,316 | 323,31 | 29685000000 | 17 | 339 |
| A0A5E8QFG8 | ^[[8]](#footnote-8)^Uncharacterized protein^8^ | 26 | 4 | 84,2 | 22,3 | 32,171 | 323,31 | 19467000000 | 16 | 351 |
| A0A1R4JDP0 | Cytochrome bc1 complex Rieske iron-sulfur subunit | 14 | 3 | 40,2 | 11,7 | 39,384 | 323,31 | 37644000000 | 14 | 352 |
| A0A5E8QGB1 | Catalase | 22 | 0 | 53,2 | 0 | 56,857 | 323,31 | 26073000000 | 25 | 366 |
| C5C834 | Uncharacterized protein | 21 | 0 | 40,6 | 0 | 42,913 | 323,31 | 77297000000 | 14 | 373 |
| A0A5E8QG41 | Peptidase | 24 | 1 | 38,2 | 1,2 | 101,19 | 323,31 | 46082000000 | 28 | 374 |
| A0A2I1XHF1 | Long-chain fatty acid--CoA ligase | 31 | 1 | 67,3 | 1,8 | 66,096 | 323,31 | 32470000000 | 30 | 376 |
| A0A1M7AV31 | ATP-dependent zinc metalloprotease FtsH | 30 | 30 | 49 | 49 | 75,054 | 323,31 | 22309000000 | 39 | 405 |
| A0A1M7ARS4 | Nucleotide-binding universal stress protein, UspA family | 13 | 0 | 52,2 | 0 | 33,217 | 323,31 | 35923000000 | 14 | 406 |
| A0A5F0I5P1 | ABC transporter substrate-binding protein | 22 | 9 | 63,4 | 33,2 | 60,578 | 323,31 | 29915000000 | 25 | 412 |
| A0A5E8QCZ5 | ABC transporter ATP-binding protein | 27 | 21 | 58,8 | 49,7 | 67,268 | 323,31 | 35553000000 | 28 | 429 |
| A0A6N4C5X5 | Peptidase S8 | 16 | 8 | 33,7 | 18,6 | 64,195 | 323,31 | 71769000000 | 18 | 433 |
| A0A031I9G7 | Glycerol-3-phosphate dehydrogenase | 31 | 2 | 62,3 | 5,1 | 63,978 | 323,31 | 27779000000 | 30 | 447 |
| A0A4Y8PM29 | ABC transporter family substrate-binding protein | 23 | 2 | 49,3 | 5,3 | 66,997 | 323,31 | 37912000000 | 26 | 531 |
| A0A1M7A9Z4 | Multiple sugar transport system ATP-binding protein | 27 | 27 | 69,8 | 69,8 | 48,069 | 323,31 | 41881000000 | 24 | 538 |
| A0A653IV33 | Dihydrolipoyl dehydrogenase | 34 | 22 | 72,5 | 52,7 | 48,952 | 323,31 | 97906000000 | 26 | 670 |
| A0A6N4BYN9 | NADH dehydrogenase FAD-containing subunit | 36 | 1 | 77,3 | 8,5 | 50,497 | 323,31 | 1,02E+11 | 25 | 687 |
| A0A4Y8PKB7 | Succinate dehydrogenase flavoprotein subunit | 37 | 16 | 71,1 | 35 | 65,935 | 323,31 | 77608000000 | 27 | 730 |
| A0A509Y352 | UPF0182 protein C0205_00430 | 37 | 21 | 47,4 | 33,5 | 112,19 | 323,31 | 58657000000 | 34 | 737 |
| A0A2N6RPI7 | ATP synthase subunit beta | 35 | 31 | 88,2 | 83,9 | 52,473 | 323,31 | 80263000000 | 23 | 1201 |
| A0A5F0I8F8 | ATP synthase subunit alpha | 39 | 0 | 77,9 | 0 | 59,244 | 323,31 | 1,47E+11 | 34 | 1227 |

1. ^1^A transmembrane, tryptophan-rich sensory protein [↑](#footnote-ref-1)
2. ^2^A putative membrane DoxX-family protein [↑](#footnote-ref-2)
3. ^3^ A putative discoidine protein [↑](#footnote-ref-3)
4. ^4^ A putative asparagine synthetase

   ^5^ A membrane binding divIVA family protein

   ^6^ A putative transmembrane peptidoglikan-binding protein [↑](#footnote-ref-4)
5. [↑](#footnote-ref-5)
6. [↑](#footnote-ref-6)
7. ^7^ A putative TPM domain containing phosphatase [↑](#footnote-ref-7)
8. ^8^ PRC and duf2382 domains-containing protein. RNA processing? [↑](#footnote-ref-8)
