# Supplementary material for: Epinephrine extensively changes the biofilm matrix composition in Micrococcus luteus C01 isolated from human skin
Source: Front Microbiol. 2022 Sep 20;13:1003942. doi: 10.3389/fmicb.2022.1003942 (PMC9530943; doi:10.3389/fmicb.2022.1003942)
Supplement: Supplementary file 3 [file Table_2.docx]

Supplementary table 2. Proteins with changed expression in epinephrine samples after 72 h of incubation in comparison with 24 h samples.

| Accession | Protein name | Fold change | Mol. weight [kDa] | MS/MS count |
| --- | --- | --- | --- | --- |
| A0A5F0IBK0 | Glycine dehydrogenase (decarboxylating) | 74,10381994 | 104,08 | 179 |
| A0A4Y8PJQ9 | ABC transporter ATP-binding protein | 32,27363025 | 29,869 | 82 |
| A0A653QWC7 | DNA protection during starvation protein | 28,87621648 | 22,266 | 10 |
| D3LMG6 | Uncharacterized protein | 25,78111214 | 6,053 | 94 |
| A0A653IWD5 | Uncharacterized protein | 24,31537002 | 12,146 | 18 |
| A0A1M7AJU5 | Methylmalonate-semialdehyde dehydrogenase [acylating] | 21,62323615 | 53,7 | 140 |
| A0A4Y9HAD5 | Siderophore-interacting protein | 18,85257763 | 31,451 | 32 |
| A0A1R4IGQ9 | ATP synthase subunit alpha | 17,01094781 | 59,291 | 166 |
| A0A5F0I6E2 | Phage holin family protein | 16,3695682 | 16,423 | 40 |
| C5C846 | Universal stress protein UspA-like protein | 14,5812817 | 12,133 | 82 |
| A0A5F0I760 | Peptidase | 14,48754213 | 101,45 | 151 |
| A0A5E8QDS8 | NfeD family protein | 13,86412569 | 16,038 | 66 |
| C5C7T0 | MIP family channel protein | 13,26398119 | 24,409 | 32 |
| A0A509Y4H3 | Protein-disulfide isomerase | 12,43455729 | 30,942 | 27 |
| C5CBK5 | Uncharacterized protein | 11,57098828 | 10,878 | 42 |
| D3LN59 | Superoxide dismutase | 11,36880625 | 22,918 | 63 |
| A0A132HEY6 | 2,3-bisphosphoglycerate-dependent phosphoglycerate mutase | 11,35285517 | 27,831 | 41 |
| A0A4U1LCZ2 | Agmatinase | 10,88247948 | 35,919 | 36 |
| A0A4Y8PND7 | Uncharacterized protein | 10,73971292 | 18,269 | 67 |
| A0A031IM63 | S-adenosylmethionine synthase | 10,40909562 | 44,787 | 108 |
| A0A653IQM1 | Thioredoxin | 10,11203801 | 11,473 | 44 |
| A0A653SH09 | Electron transfer flavoprotein subunit beta | 9,986140575 | 27,068 | 94 |
| A0A509Y330 | Citrate synthase | 9,907278761 | 48,148 | 54 |
| P33102 | 50S ribosomal protein L18 | 9,716038247 | 12,961 | 27 |
| A0A132HR96 | Chaperone protein DnaK | 9,398240728 | 66,474 | 28 |
| A0A2I1XVZ4 | FHA domain-containing protein | 9,341900635 | 19,306 | 71 |
| A0A5E8QCD1 | Phosphate transport system permease protein | 9,310603043 | 33,737 | 30 |
| A0A2N6RPI7 | ATP synthase subunit beta | 9,257121514 | 52,473 | 1201 |
| A0A2I1XWJ2 | DUF3710 domain-containing protein | 9,193917874 | 27,91 | 55 |
| A0A4V6WRJ9 | Protein-tyrosine-phosphatase | 8,917089996 | 27,189 | 24 |
| A0A1M7DLB9 | 2,4-dienoyl-CoA reductase | 8,780989489 | 40,054 | 17 |
| A0A378NJ14 | Magnesium transport protein CorA | 8,576374259 | 41,498 | 36 |
| D3LR19 | Uncharacterized protein | 8,267816289 | 5,3583 | 13 |
| A0A031HM15 | Elongation factor Tu | 8,125019594 | 43,785 | 140 |
| A0A2N6RPB6 | ABC transporter | 7,892630781 | 29,058 | 28 |
| A0A031HS73 | Alanine aminopeptidase | 7,723290146 | 96,24 | 78 |
| A0A509Y696 | Amino acid ABC transporter ATP-binding protein | 7,654502125 | 28,079 | 40 |
| A0A4Y8PMY3 | Catalase OX=566027 | 7,616465489 | 80,137 | 73 |
| D3LKZ8 | Cyclic nucleotide-binding domain protein | 7,546393755 | 24,521 | 60 |
| A0A5E8QR72 | Integral membrane protein | 7,468908085 | 25,361 | 69 |
| D3LPR4 | Antibiotic biosynthesis monooxygenase | 7,415557015 | 11,959 | 32 |
| A0A5E8QCA6 | Protein kinase | 7,338066137 | 55,566 | 6 |
| A0A653SAT3 | 3-hydroxyacyl-CoA dehydrogenase | 7,251864784 | 76,979 | 74 |
| A0A1M7EC07 | Putative efflux protein, MATE family | 7,18555587 | 47,675 | 10 |
| A0A378NH58 | Manganese transport protein MntH | 7,14986842 | 48,117 | 49 |
| A0A4Y8PN11 | Fructose-bisphosphate aldolase | 6,982236968 | 36,325 | 74 |
| A0A4U1LD92 | DUF2029 domain-containing protein | 6,942004498 | 50,355 | 64 |
| A0A5F0I8C1 | Uncharacterized protein | 6,89275431 | 31,85 | 10 |
| A0A1M7BC23 | Methionine aminopeptidase | 6,872362912 | 33,907 | 33 |
| A0A2N6RP99 | DUF4233 domain-containing protein | 6,667120146 | 18,612 | 21 |
| A0A031HT42 | Isoleucine--tRNA ligase | 6,629112399 | 125,71 | 44 |
| A0A5F0UMP1 | Uncharacterized protein | 6,540456237 | 19,163 | 20 |
| A0A2N6RL67 | Uncharacterized protein | 6,440595954 | 72,811 | 194 |
| A0A653PUG0 | Chlorite O(2)-lyase | 6,406459519 | 27,175 | 115 |
| A0A031H213 | Acyl-CoA thioester hydrolase, YbgC/YbaW family | 6,28657317 | 16,86 | 47 |
| A0A562FTX5 | 3-oxoacyl-[acyl-carrier protein] reductase | 6,269732118 | 45,156 | 91 |
| A0A1R4IF19 | 2,3-butanediol dehydrogenase, R-alcohol forming, (R)-and (S)-acetoin-specific | 6,269367411 | 37,876 | 34 |
| A0A5E8QCZ2 | LysM peptidoglycan-binding domain-containing protein | 6,255345052 | 71,408 | 33 |
| C5CBB4 | Amino acid ABC transporter, permease protein, 3-TM region, His/Glu/Gln/Arg/opine family | 6,244270278 | 31,476 | 32 |
| D3LQS1 | Transporter, major facilitator family protein | 6,233113475 | 48,158 | 16 |
| A0A031IAG7 | Transaldolase | 6,210460037 | 39,18 | 25 |
| A0A5F0IA42 | Hydroxymethylpyrimidine kinase e | 6,160000751 | 29,43 | 40 |
| A0A509Y5P3 | Copper resistance protein CopC | 6,134105919 | 22,764 | 23 |
| A0A562FMT2 | Lsr2 protein | 6,106034961 | 9,1163 | 19 |
| A0A132HM90 | Trehalose transport system permease protein SugB | 6,036514056 | 32,757 | 31 |
| D3LMB8 | 2-oxo acid dehydrogenase acyltransferase (Catalytic domain) (Fragment) | 5,959401043 | 25,79 | 32 |
| A0A2I1XT33 | Oxaloacetate decarboxylase | 5,921389564 | 56,192 | 58 |
| A0A5F0I661 | Protoheme IX farnesyltransferase | 5,743598308 | 36,46 | 55 |
| A0A2N6REI0 | 3,4-dihydroxyphenylacetate 2,3-dioxygenase | 5,657158395 | 44,265 | 73 |
| C5C9L0 | ABC-type spermidine/putrescine transport system, ATPase component | 5,622712381 | 40,392 | 52 |
| A0A4Y8PL89 | FAD-dependent oxidoreductase | 5,608794043 | 55,386 | 79 |
| A0A378NKV4 | Protein translocase subunit SecE | 5,453713534 | 7,9915 | 37 |
| A0A6N4F6I1 | Sodium:glutamate symporter | 5,344670443 | 48,364 | 32 |
| A0A6N4F9W0 | ATP-binding cassette domain-containing protein | 5,277960469 | 27,172 | 114 |
| D3LKU8 | Nucleoside diphosphate kinase | 5,270666681 | 15,291 | 73 |
| A0A4Y8PME9 | Uncharacterized protein | 5,147477199 | 37,562 | 17 |
| A0A1M7ASZ6 | Phosphate import ATP-binding protein PstB | 5,131859993 | 28,468 | 234 |
| C5C8M8 | Uncharacterized protein | 5,111496661 | 31,71 | 124 |
| A0A653IVB3 | Dipeptide transporter membrane component of ABC superfamily | 5,075154766 | 31,453 | 23 |
| A0A031HXD9 | Uncharacterized protein | 4,994175011 | 35,089 | 13 |
| A0A6N4C321 | Cytochrome c oxidase subunit | 4,928765349 | 63,977 | 113 |
| A0A1M6ZPK3 | Translation factor SUA5 | 4,840083858 | 25,616 | 84 |
| D3LQT0 | Imidazoleglycerol-phosphate dehydratase | 4,835470285 | 22,498 | 21 |
| A0A031IS63 | L-lactate permease | 4,785739981 | 57,411 | 43 |
| A0A4Y8ZJ85 | 30S ribosomal protein S3 (Fragment) | 4,724365457 | 26,08 | 6 |
| A0A5F0I6M5 | Lactoylglutathione lyase | 4,711383407 | 15,103 | 30 |
| A0A562FQF8 | Uncharacterized protein (Fragment) | 4,691088156 | 13,487 | 26 |
| A0A031GTE4 | Uncharacterized protein | 4,60387427 | 62,278 | 44 |
| D3LSF8 | Uncharacterized protein | 4,584682568 | 20,15 | 35 |
| A0A031H0A6 | Demethylmenaquinone methyltransferase | 4,517003663 | 26,868 | 16 |
| A0A031IM29 | 3-hydroxybutyryl-CoA dehydrogenase | 4,516898162 | 33,709 | 8 |
| A0A031H0K6 | PA domain protein | 4,478785439 | 48,741 | 20 |
| A0A410XQB1 | Uncharacterized protein | 4,459598277 | 53,792 | 50 |
| A0A1M7ARS4 | Nucleotide-binding universal stress protein, UspA family | 4,334408686 | 33,217 | 406 |
| A0A031G1Y7 | Uncharacterized protein | 4,230183555 | 14,661 | 66 |
| A0A653J027 | DUF21 domain-containing protein | 4,142150781 | 35,516 | 16 |
| A0A5F0UN76 | F0F1 ATP synthase subunit epsilon | 4,141345854 | 9,2052 | 108 |
| A0A653LY24 | ABC-type Fe3+ transport system, periplasmic component | 4,140017428 | 38,583 | 151 |
| C5C750 | Mn-containing catalase | 4,123035531 | 31,399 | 27 |
| A0A410XQ89 | DNA gyrase subunit A | 4,106128721 | 99,199 | 74 |
| D3LR61 | Glutamine--fructose-6-phosphate aminotransferase [isomerizing] | 4,056032247 | 67,659 | 11 |
| C5CCF3 | Uncharacterized protein | 4,036923011 | 21,792 | 58 |
| A0A378NNU4 | Acyl-CoA dehydrogenase, short-chain specific | 4,012220485 | 42,21 | 90 |
| C5C835 | Peptidyl-prolyl cis-trans isomerase | 3,978271462 | 19,094 | 24 |
| A0A562FW94 | Putative zinc finger protein | 3,972672215 | 30,458 | 26 |
| A0A378NNE4 | Daunorubicin/doxorubicin resistance ATP-binding protein DrrA | 3,798429275 | 29,108 | 127 |
| A0A132HXJ4 | Ribonucleoside-diphosphate reductase subunit beta | 3,796262149 | 37,516 | 109 |
| A0A031H4U6 | Alanine--tRNA ligase | 3,735174946 | 97,165 | 25 |
| A0A5F0UJA9 | Disulfide bond formation protein DsbA | 3,733978454 | 26,507 | 12 |
| A0A1M7DEP3 | Forkhead associated (FHA) domain, binds pSer, pThr, pTyr | 3,723028721 | 32,3 | 14 |
| A0A5F0IAH8 | Iron-siderophore ABC transporter substrate-binding protein | 3,673344666 | 37,506 | 199 |
| D3LLM3 | TIGR01906 family protein | 3,656228515 | 43,555 | 32 |
| A0A4Y8PLK1 | Heme A synthase | 3,649718352 | 35,043 | 55 |
| A0A5F0ICU2 | FAD-binding protein | 3,608116396 | 50,915 | 63 |
| A0A653IZW6 | Copper (Cu(I)) transporter ATPase | 3,591748934 | 84,519 | 8 |
| A0A4U1LBE9 | Nif3-like dinuclear metal center hexameric protein | 3,566270344 | 31,599 | 34 |
| D3LPH3 | SNARE-like domain protein | 3,531829007 | 23,382 | 6 |
| D3LNP8 | Hydrolase, alpha/beta domain protein | 3,486727278 | 30,406 | 22 |
| A0A5F0I6C5 | M13 family peptidase | 3,459478758 | 75,904 | 46 |
| A0A031G3L1 | Dolichyl-phosphate-mannose--protein mannosyltransferase | 3,411191508 | 59,38 | 17 |
| A0A5E8QCZ5 | ABC transporter ATP-binding protein | 3,407199626 | 67,268 | 429 |
| D3LKR4 | Signal recognition particle protein | 3,406608077 | 56,531 | 47 |
| A0A5F0UMV5 | Ketol-acid reductoisomerase (NADP(+)) | 3,394683456 | 36,969 | 19 |
| A0A653MZQ1 | NAD(P)/FAD-dependent oxidoreductase | 3,341442125 | 42,16 | 69 |
| A0A031GU25 | Putative succinyl-diaminopimelate desuccinylase DapE | 3,332946058 | 39,38 | 22 |
| A0A1R4JVW6 | Lactoylglutathione lyase | 3,302766035 | 15,194 | 21 |
| A0A031H5M9 | Methionine aminopeptidase | 3,285534364 | 29,093 | 41 |
| A0A132HZN4 | Ureidoglycolate lyase | 3,240933066 | 32,803 | 57 |
| A0A1M7BQ59 | Trk system potassium uptake protein TrkA | 3,19927539 | 24,21 | 51 |
| D3LSC8 | 7,8-dihydroneopterin aldolase | 3,155928509 | 13,483 | 24 |
| A0A562FP97 | Non-specific serine/threonine protein kinase | 3,134171238 | 41,072 | 30 |
| A0A031GAL4 | Non-specific serine/threonine protein kinase | 3,096066758 | 79,423 | 85 |
| A0A031GFD9 | 4-hydroxy-tetrahydrodipicolinate synthase | 3,087734628 | 31,609 | 12 |
| A0A031HQ70 | Redoxin family protein | 3,072896178 | 21,479 | 32 |
| A0A653IV33 | Dihydrolipoyl dehydrogenase | 3,04108798 | 48,952 | 670 |
| A0A4Y8PLS3 | Sec-independent protein translocase protein TatB | 3,035226327 | 21,107 | 33 |
| A0A031HMU6 | ABC-type uncharacterized transport system, periplasmic component | 3,007257085 | 34,151 | 32 |
| A0A6N4F5Q6 | BCCT family transporter | 2,988522013 | 76,283 | 90 |
| D3LLQ3 | Transcriptional regulator, AsnC family | 2,984960295 | 10,042 | 23 |
| A0A031GMP1 | Phenylacetate-CoA oxygenase, PaaI subunit | 2,916706327 | 30,793 | 10 |
| D3LQ56 | Elongation factor P | 2,90748173 | 20,418 | 10 |
| A0A4Y8PLP1 | Antigen 84 | 2,882218516 | 21,279 | 78 |
| A0A031ID36 | Diaminopimelate epimerase | 2,842845437 | 33,219 | 21 |
| A0A6N4C881 | Methionine ABC transporter ATP-binding protein | 2,834799978 | 22,113 | 36 |
| C5CBT1 | 3-ketoacyl-CoA thiolase | 2,752446153 | 46,53 | 147 |
| D3LLH5 | Uncharacterized protein | 2,74556989 | 10,606 | 22 |
| A0A5F0IC01 | PLP-dependent aminotransferase family protein | 2,74384021 | 43,261 | 14 |
| C5CCA5 | Cytochrome aa3 subunit 3 | 2,740148632 | 23,513 | 15 |
| A0A6N4FHA7 | Dipeptide ABC transporter ATP-binding protein | 2,738476673 | 62,593 | 15 |
| A0A509Y3R5 | NAD-dependent succinate-semialdehyde dehydrogenase | 2,729489764 | 49,102 | 152 |
| A0A378NMW3 | Putrescine importer PuuP | 2,690122869 | 58,785 | 33 |
| A0A562FT77 | Histidine kinase | 2,667896724 | 68,731 | 11 |
| A0A1R4JN49 | Undecaprenyl-phosphate galactosephosphotransferase | 2,658571975 | 54,409 | 12 |
| A0A031GZR4 | Acetyl-coenzyme A synthetase | 2,656948621 | 71,087 | 44 |
| A0A5F0I679 | Uncharacterized protein e | 2,653893176 | 13,612 | 55 |
| A0A4Y8PHL4 | Uncharacterized protein | 2,647909511 | 30,739 | 44 |
| A0A2N6RHY6 | Uncharacterized protein | 2,606296541 | 19,594 | 50 |
| C5CA40 | Uncharacterized protein | 2,563471723 | 13,663 | 33 |
| A0A5E8QB86 | DNA recombination protein RmuC | 2,532819062 | 45,064 | 87 |
| D3LPC0 | DUF3566 domain-containing protein | 2,525556395 | 17,063 | 44 |
| A0A2N6RKZ3 | RNA polymerase subunit sigma-70 | 2,474347558 | 50,766 | 231 |
| A0A5F0I8F8 | ATP synthase subunit alpha | 2,465675391 | 59,244 | 1227 |
| A0A031HRC0 | Trigger factor | 2,452786249 | 52,178 | 42 |
| A0A5F0IBI2 | Uncharacterized protein | 2,408520412 | 31,627 | 13 |
| A0A5E8QRC0 | Amino acid ABC transporter ATP-binding protein | 2,399505757 | 28,655 | 97 |
| A0A031HY75 | Magnesium transporter | 2,396904805 | 46,828 | 41 |
| A0A4Y8PKQ5 | RidA family protein | 2,381084307 | 16,784 | 33 |
| A0A031G5V6 | CAAX amino terminal protease family protein | 2,36352495 | 29,092 | 11 |
| A0A5F0IB21 | Uncharacterized protein e | 2,357756415 | 16,63 | 14 |
| A0A2I1XS51 | IMP dehydrogenase | 2,350594805 | 37,348 | 31 |
| A0A2N6RS12 | Uncharacterized protein | 2,345314617 | 37,896 | 21 |
| A0A4Y8PM05 | Uncharacterized protein | 2,326003949 | 26,358 | 92 |
| A0A5F0UNM1 | UDP-N-acetyl-D-mannosamine dehydrogenase | 2,315222942 | 44,11 | 10 |
| A0A4Y8PK61 | ABC transporter ATP-binding protein | 2,304377149 | 66,796 | 46 |
| A0A562FPF4 | 1-acyl-sn-glycerol-3-phosphate acyltransferase | 2,285050064 | 25,501 | 39 |
| A0A2I1XHF1 | Long-chain fatty acid--CoA ligase | 2,261947205 | 66,096 | 376 |
| C5C8J5 | Predicted membrane protein | 2,249873166 | 28,978 | 15 |
| A0A1M7CQB9 | Polyribonucleotide nucleotidyltransferase | 2,247994069 | 80,453 | 29 |
| A0A509Y333 | Glutamate ABC transporter substrate-binding protein | 2,247143244 | 31,074 | 210 |
| A0A031I9N9 | Phosphoglycerate kinase | 2,237444479 | 42,677 | 13 |
| A0A2N6RSL8 | DUF4190 domain-containing protein | 2,234681381 | 19,377 | 14 |
| A0A1R4K9B9 | Cell division inhibitor | 2,234055133 | 55,954 | 39 |
| D3LLW5 | Uncharacterized protein | 2,23135817 | 15,013 | 22 |
| C5C7D5 | CAAX amino terminal protease family | 2,229094688 | 33,676 | 15 |
| A0A4Y8PHX6 | Uncharacterized protein | 2,224083508 | 74,713 | 153 |
| A0A5F0I888 | ABC transporter | 2,214901019 | 64,231 | 138 |
| A0A5E8QGG3 | Succinate--CoA ligase [ADP-forming] subunit beta | 2,202887824 | 40,796 | 74 |
| A0A031IFF2 | Foldase YidC | 2,202362688 | 36,847 | 119 |
| D3LR86 | 10 kDa chaperonin | 2,202190905 | 10,346 | 13 |
| A0A5F0I8I3 | Threonine/serine exporter family protein | 2,188927586 | 67,496 | 28 |
| A0A653IPQ6 | Putative membrane protein | 2,184908727 | 60,233 | 159 |
| A0A270B1W3 | 3-dehydroquinate synthase | 2,143138501 | 41,373 | 29 |
| A0A031I9G7 | Glycerol-3-phosphate dehydrogenase | 2,141875183 | 63,978 | 447 |
| A0A031GXC0 | DivIVA domain protein | 2,13638681 | 13,214 | 42 |
| C5C8Q0 | Micrococcal nuclease-like nuclease | 2,129877892 | 29,211 | 7 |
| A0A031GTE1 | Cation diffusion facilitator family transporter | 2,125290181 | 35,015 | 23 |
| A0A132HXX3 | Chromosome partition protein Smc | 2,08670113 | 84,389 | 42 |
| A0A653RVD7 | MFS transporter | 2,086405043 | 51,688 | 52 |
| A0A6N4C0D6 | Sodium:solute symporter | 2,071516274 | 52,012 | 12 |
| A0A4U1MBF7 | Acyl-CoA dehydrogenase | 2,039036138 | 77,845 | 174 |
| A0A1R4J414 | ATP synthase gamma chain | 1,995588267 | 32,65 | 331 |
| A0A6N4C379 | Uncharacterized protein | 1,975990992 | 20,817 | 147 |
| A0A031I8P0 | SURF1-like protein | 1,967628144 | 33,562 | 29 |
| C5CCN1 | Uncharacterized protein | 1,96744259 | 29,125 | 116 |
| A0A031GSR4 | NAD dependent epimerase/dehydratase family | 1,950869196 | 36,562 | 5 |
| A0A031H0M4 | ABC transporter, ATP-binding protein | 1,941967283 | 64,585 | 63 |
| A0A1M7DTY7 | Calcium-transporting ATPase CtpE | 1,922001086 | 88,245 | 84 |
| A0A132HYV3 | S-(Hydroxymethyl)mycothiol dehydrogenase | 1,915413356 | 39,638 | 312 |
| A0A1M7AKE6 | Membrane protein | 1,902145444 | 39,05 | 61 |
| D3LQK8 | ATP synthase subunit delta | 1,899199572 | 28,627 | 332 |
| A0A653PIY1 | Phosphatidylglycerol--prolipoprotein diacylglyceryl transferase | 1,857147829 | 36,206 | 27 |
| A0A378NUP4 | Beta-(1-->2)glucan export ATP-binding/permease protein NdvA | 1,795605402 | 9,0263 | 12 |
| A0A653IWI6 | MarR family transcriptional regulator | 1,747096885 | 16,057 | 47 |
| D3LM24 | 30S ribosomal protein S7 | 1,726847419 | 17,146 | 14 |
| A0A5F0IAF3 | Uncharacterized protein | 1,724649511 | 64,039 | 46 |
| A0A5E8QF41 | Glutaminase OX=1270 | 1,718256551 | 65,549 | 71 |
| A0A2I1XY38 | Uncharacterized protein | 1,713183862 | 53,742 | 183 |
| A0A5E8QFG8 | Uncharacterized protein | 1,676041718 | 32,171 | 351 |
| A0A031G7E5 | Thymidine phosphorylase | 1,67181331 | 46,184 | 10 |
| A0A4U1LL35 | NADP-dependent oxidoreductase | 1,669976857 | 34,828 | 14 |
| A0A653IY01 | Metallophosphoesterase | 1,616597744 | 33,418 | 45 |
| A0A1R4JC68 | Lead, cadmium, zinc and mercury transporting ATPase Copper-translocating P-type ATPase | 1,54334624 | 77,355 | 65 |
| A0A2I1XKK3 | Phosphoenolpyruvate carboxykinase [GTP] | 1,527187665 | 67,8 | 47 |
| A0A1R4J0T4 | Cysteine synthase | 1,519091928 | 32,51 | 23 |
| A0A509Y352 | UPF0182 protein C0205_00430 | 1,514414332 | 112,19 | 737 |
| A0A5E8QD19 | 1-deoxy-D-xylulose-5-phosphate synthase | 1,495067896 | 71,346 | 23 |
| A0A031GPQ3 | Bacterial type II secretion system domain protein F | 1,494558682 | 30,755 | 11 |
| A0A6N4BYN9 | NADH dehydrogenase FAD-containing subunit | 1,454945788 | 50,497 | 687 |
| A0A031G0E8 | Geranylgeranyl reductase family protein | 1,45247421 | 44,564 | 9 |
| A0A5E8QG41 | Peptidase OX=1270 | 1,340483086 | 101,19 | 374 |
| A0A1M7BCP4 | Cell division protein FtsZ | 1,309024363 | 41,949 | 69 |
| A0A6N4F9N0 | ATP-binding cassette domain-containing protein | 0,741644632 | 27,645 | 70 |
| A0A2N6RT58 | Aldehyde dehydrogenase | 0,724072345 | 53,525 | 119 |
| A0A031I5Q8 | Putative membrane protein | 0,707126413 | 30,35 | 71 |
| A0A4Y8ZJP3 | Uncharacterized protein (Fragment) | 0,698975233 | 16,108 | 13 |
| A0A5F0I7W1 | DNA translocase FtsK | 0,674260527 | 110,88 | 118 |
| A0A653SJD9 | Thiosulfate reductase | 0,656694092 | 60,185 | 77 |
| A0A1R4JDP0 | Cytochrome bc1 complex Rieske iron-sulfur subunit | 0,641858312 | 39,384 | 352 |
| D3LS08 | Response regulator receiver domain protein | 0,637343675 | 25,282 | 19 |
| A0A6N4F717 | MFS transporter | 0,629239132 | 54,258 | 57 |
| A0A5E8QFG0 | NAD_binding_9 domain-containing protein | 0,627546554 | 70,238 | 18 |
| A0A6N4FFH9 | TerC/Alx family metal homeostasis membrane protein | 0,621253252 | 46,387 | 331 |
| A0A031GNH5 | Hydrolase, NUDIX family | 0,618366936 | 26,277 | 6 |
| A0A031GV11 | Cation/acetate symporter ActP | 0,607317691 | 56,011 | 128 |
| A0A6N4C3M0 | Uncharacterized protein | 0,602640594 | 31,69 | 40 |
| C5CC75 | 50S ribosomal protein L11 | 0,600602282 | 14,843 | 6 |
| A0A653J101 | ABC transporter permease | 0,599648075 | 19,276 | 14 |
| A0A653R2Q1 | 5-oxoproline transporter | 0,598305029 | 44,071 | 95 |
| A0A4Y8PM30 | Dicarboxylate/amino acid:cation symporter | 0,598073046 | 44,604 | 66 |
| A0A031H0D1 | ABC transporter, permease protein | 0,596698816 | 35,456 | 43 |
| A0A1M7B968 | Cu2+-exporting ATPase | 0,591652252 | 74,404 | 221 |
| A0A5F0IA71 | PhoX family phosphatase | 0,590139834 | 76,32 | 38 |
| A0A562G510 | Carbon starvation protein | 0,58823375 | 79,422 | 297 |
| A0A4Y8X2Q8 | D-inositol 3-phosphate glycosyltransferase | 0,587772762 | 79,218 | 28 |
| A0A5F0I9S4 | ABC transporter permease | 0,576073977 | 48,23 | 84 |
| A0A6N4FI86 | Sphingosine kinase | 0,564826047 | 42,058 | 78 |
| A0A5E8QA42 | ABC transporter ATP-binding protein | 0,563611659 | 62,606 | 283 |
| A0A5F0UMS7 | Amino acid adenylation domain-containing protein | 0,56024554 | 140,69 | 82 |
| A0A1R4J497 | Uncharacterized protein | 0,559014394 | 12,022 | 7 |
| A0A4Y8PMK5 | Patatin family protein | 0,553727451 | 33,415 | 12 |
| A0A5F0IB40 | MFS transporter | 0,548850783 | 49,763 | 45 |
| A0A4U1KYN1 | Sugar transferase | 0,539692352 | 54,076 | 73 |
| A0A562FUA9 | UPF0234 protein L597_002200000040 | 0,539234233 | 17,953 | 7 |
| A0A031GAF7 | FHA domain protein | 0,53784704 | 11,036 | 7 |
| A0A4Y8X1K9 | Adenylosuccinate synthetase | 0,537296003 | 46,598 | 8 |
| A0A2N6RLF0 | Dephospho-CoA kinase | 0,530980329 | 85,336 | 189 |
| C5CCE9 | Uncharacterized protein | 0,527649558 | 11,222 | 8 |
| A0A2I1XTF6 | Uncharacterized protein | 0,524949602 | 33,493 | 9 |
| A0A509Y4A8 | Methylamine utilization protein MauE | 0,513729766 | 36,182 | 33 |
| A0A509Y5N1 | Na+/H+ antiporter subunit A | 0,513669322 | 110,2 | 161 |
| A0A031GJB7 | Endonuclease/exonuclease/phosphatase family protein | 0,509219495 | 94,189 | 93 |
| A0A2N6RSH2 | NDP-hexose 4-ketoreductase | 0,499031639 | 93,374 | 137 |
| D3LKW4 | ABC transporter, permease protein | 0,4971575 | 29,758 | 46 |
| A0A132I1X4 | DUF445 domain-containing protein | 0,489169824 | 46,788 | 130 |
| A0A4Y8PKX5 | D-inositol 3-phosphate glycosyltransferase | 0,488763422 | 42,776 | 4 |
| A0A1R4JL91 | Phosphoribosylformylglycinamidine synthase subunit PurL | 0,487622308 | 82,031 | 5 |
| A0A653IT60 | DUF4352 domain-containing protein | 0,478204029 | 21,342 | 131 |
| A0A4P8H6F4 | TrwC relaxase | 0,476702339 | 98,624 | 2 |
| A0A509Y3B2 | HlyC/CorC family transporter | 0,474904525 | 48,762 | 148 |
| A0A6N4C708 | Uncharacterized protein | 0,470773705 | 18,957 | 27 |
| A0A6N4FHY7 | Uncharacterized protein | 0,469935875 | 19,328 | 53 |
| D3LME7 | Sec-independent protein translocase protein TatA | 0,469510277 | 9,2525 | 40 |
| C5CCW0 | Putative hydrolase, NUDIX family domain protein | 0,465022283 | 32,46 | 13 |
| A0A6N4FF78 | Amidohydrolase | 0,458012697 | 43,214 | 18 |
| A0A653Q1J6 | Putative transporter subunit: ATP-binding component of ABC superfamily transporter | 0,457612417 | 36,14 | 102 |
| A0A653J3L8 | Adenylate cyclase | 0,455239139 | 40,683 | 13 |
| A0A031IIG8 | Phospho-N-acetylmuramoyl-pentapeptide-transferase OX=1270 | 0,45435146 | 39,51 | 32 |
| A0A378NM65 | Protein of uncharacterized function (DUF3710) | 0,449074285 | 27,996 | 7 |
| A0A4Y8PM59 | Protein-export membrane protein SecF | 0,447570765 | 36,961 | 73 |
| A0A653T9U7 | Serine/threonine-protein kinase | 0,446348363 | 70,324 | 337 |
| A0A031G8C8 | Na+/H+ antiporter subunit D | 0,44417696 | 57,039 | 4 |
| A0A5F0I8B3 | Uncharacterized protein | 0,442673255 | 26,742 | 22 |
| A0A1M7BLA2 | Menaquinone-dependent protoporphyrinogen oxidase | 0,44119 | 17,159 | 26 |
| A0A5F0I8I8 | Preprotein translocase subunit YajC | 0,435339317 | 15,163 | 86 |
| A0A4Y8PNF9 | 3-phosphoshikimate 1-carboxyvinyltransferase | 0,43489886 | 49,027 | 6 |
| A0A410XQF5 | WcbI domain-containing protein | 0,430036524 | 34,262 | 5 |
| A0A031GVR7 | Elongation factor Ts | 0,428344287 | 29,408 | 61 |
| D3LRV9 | Uncharacterized protein | 0,427456114 | 8,5065 | 29 |
| A0A031GMJ1 | Uncharacterized protein | 0,425977101 | 77,766 | 111 |
| A0A4U1ME21 | Protein GrpE | 0,425850218 | 23,381 | 18 |
| A0A031HYU7 | Putative membrane protein | 0,425403614 | 36,956 | 26 |
| A0A031ICM1 | Mn2+/Fe2 transporter | 0,420956945 | 48,216 | 6 |
| A0A2N6RI12 | 1-pyrroline-5-carboxylate dehydrogenase | 0,414871487 | 129,29 | 267 |
| C5CCA8 | Cytochrome bc1 complex cytochrome b subunit | 0,410901071 | 62,755 | 267 |
| A0A031H077 | Cell envelope-like function transcriptional attenuator common domain protein | 0,408189685 | 39,425 | 83 |
| A0A031GJH5 | Aminotransferase, class III | 0,407704822 | 50,187 | 5 |
| A0A031ISH3 | ABC transporter, ATP-binding protein | 0,405782745 | 56,721 | 234 |
| A0A5E8QGG7 | Vitamin K epoxide reductase | 0,402190236 | 23,841 | 104 |
| A0A5F0IAS9 | Uncharacterized protein | 0,393389188 | 42,886 | 22 |
| C5CCJ9 | Amino acid transporter | 0,39217717 | 68,754 | 50 |
| A0A5E8QFN5 | Histidine kinase | 0,392147099 | 64,426 | 51 |
| D3LRU5 | Uncharacterized protein | 0,390261045 | 50,135 | 64 |
| A0A4Y9HBN9 | Thiol reductant ABC exporter subunit CydC | 0,386858118 | 120,44 | 125 |
| A0A4Y8PKF3 | FMN-binding glutamate synthase family protein | 0,375642671 | 72,928 | 169 |
| A0A653IT38 | Uncharacterized protein | 0,374379394 | 94,68 | 10 |
| A0A4Y8PKE3 | Alanine:cation symporter family protein | 0,374045942 | 50,476 | 107 |
| A0A6N4FGM8 | PqqD family peptide modification chaperone | 0,366680259 | 38,201 | 6 |
| A0A653IQ80 | (Fe-S)-binding protein | 0,366304748 | 30,726 | 59 |
| A0A031ICS1 | Sec-independent protein translocase protein TatC | 0,36607156 | 31,534 | 65 |
| A0A031HWZ9 | Amino acid permease | 0,364521619 | 53,123 | 24 |
| A0A653TT59 | Magnesium transporter MgtE | 0,363968308 | 48,299 | 8 |
| A0A6N4F5K7 | Pyridine nucleotide-disulfide oxidoreductase | 0,361825792 | 52,167 | 8 |
| A0A4V5PZD3 | DUF1461 domain-containing protein | 0,360598652 | 43,464 | 172 |
| A0A2N6RI54 | Uncharacterized protein | 0,358593368 | 9,484 | 35 |
| A0A031G8X7 | Cell cycle protein, FtsW/RodA/SpoVE family | 0,356122446 | 50,072 | 74 |
| A0A378NKZ8 | Vibriobactin utilization protein ViuB | 0,35513134 | 29,747 | 6 |
| D3LLS9 | Putative mycothione reductase | 0,352716759 | 54,236 | 14 |
| A0A4Y8WZ50 | Glycerol-3-phosphate dehydrogenase | 0,351845668 | 62,766 | 6 |
| A0A5F0I610 | SPFH/Band 7/PHB domain protein | 0,350283553 | 42,247 | 48 |
| D3LR26 | 30S ribosomal protein | 0,350062106 | 10,675 | 7 |
| A0A1R4JE65 | Cytochrome bc1 complex cytochrome b subunit | 0,348062646 | 62,798 | 35 |
| A0A562FSS5 | 50S ribosomal protein L3 | 0,347844597 | 22,969 | 8 |
| A0A2N6RSP9 | Nitrate reductase | 0,346914288 | 45,38 | 81 |
| A0A031HLY3 | Amidohydrolase, imidazolonepropionase | 0,344708515 | 43,201 | 86 |
| A0A2N6RGF1 | DUF3071 domain-containing protein | 0,343105502 | 51,504 | 8 |
| D3LKU5 | 50S ribosomal protein L21 | 0,339635358 | 10,679 | 9 |
| A0A509Y375 | M3 family metallopeptidase | 0,33789627 | 74,613 | 8 |
| A0A4Y8PJY5 | Septum formation initiator family protein | 0,337489252 | 28,24 | 48 |
| A0A653LS22 | Putative phosphomannomutase | 0,335225792 | 58,888 | 4 |
| A0A653NMS3 | Putative short-chain fatty acid transporter | 0,333784239 | 48,95 | 81 |
| A0A562FRP6 | Protein-disulfide isomerase | 0,333012292 | 31,042 | 39 |
| A0A031H0A1 | Siderophore-interacting FAD-binding domain protein | 0,330217677 | 37,238 | 4 |
| A0A1R4JH83 | TPR-repeat-containing protein | 0,32782394 | 49,061 | 9 |
| D3LMB9 | 50S ribosomal protein L31 type B | 0,325990428 | 9,6187 | 13 |
| A0A5F0IBV8 | Sodium:proton antiporter | 0,32276407 | 11,837 | 44 |
| C5CA36 | Cell division protein FtsI/penicillin-binding protein 2 | 0,320473084 | 65,08 | 23 |
| A0A031H5N4 | DNA-directed RNA polymerase subunit alpha | 0,319812246 | 36,215 | 9 |
| P33100 | 50S ribosomal protein L14 | 0,319030431 | 13,297 | 8 |
| A0A1M7AKN8 | Pyruvate dehydrogenase (Quinone) | 0,317255888 | 61,454 | 7 |
| A0A031HMF0 | Glycosyltransferase | 0,315035374 | 22,996 | 8 |
| A0A1M7EC16 | SulP family inorganic anion transporter | 0,311888436 | 52,267 | 52 |
| A0A5F0I8C2 | Transport permease protein | 0,309708493 | 32,627 | 26 |
| A0A031IVK8 | Basic membrane protein | 0,306038201 | 40,358 | 23 |
| A0A031H3N9 | ABC transporter, solute-binding protein | 0,302018114 | 48,6 | 5 |
| A0A653NTK0 | Putative membrane protein | 0,297939472 | 14,155 | 32 |
| A0A031GGI2 | ABC transporter, ATP-binding protein | 0,291114999 | 35,347 | 60 |
| D3LS10 | CarD-like protein | 0,28250053 | 18,073 | 8 |
| D3LMD2 | Undecaprenyl-diphosphatase SK58 | 0,274542725 | 29,613 | 19 |
| D3LRI2 | 30S ribosomal protein S6 | 0,267916279 | 11,65 | 9 |
| A0A5F0IAR4 | PHP domain-containing protein | 0,265457875 | 30,579 | 15 |
| C5CAS8 | Uncharacterized protein | 0,258299244 | 17,707 | 9 |
| D3LS33 | Aconitate hydratase | 0,258161091 | 97,728 | 74 |
| A0A653LU13 | Inositol-1-monophosphatase | 0,246288778 | 31,378 | 14 |
| C5CBL7 | Multisubunit Na+/H+ antiporter, MnhE subunit | 0,2462687 | 15,587 | 108 |
| A0A031GHI5 | SDR family NAD(P)-dependent oxidoreductase | 0,245012766 | 31,458 | 13 |
| A0A5F0I8C9 | 30S ribosomal protein S16 | 0,240341312 | 16,376 | 55 |
| A0A5E8QH35 | CDP-alcohol phosphatidyltransferase family protein | 0,235973674 | 22,076 | 26 |
| A0A031ILR8 | Membrane protein | 0,228657831 | 14,006 | 58 |
| A0A1R4I9U9 | Urocanate hydratase | 0,228399408 | 61,539 | 11 |
| A0A2N6RPG8 | Acetyl-CoA C-acyltransferase | 0,2259606 | 41,282 | 28 |
| A0A4P8H8J1 | 30S ribosomal protein S13 | 0,225539873 | 13,774 | 13 |
| A0A2I1XXJ8 | Acetyl-/propionyl-CoA carboxylase subunit alpha | 0,214573556 | 65,464 | 24 |
| A0A4Y8PKD2 | Acyl-CoA thioesterase II | 0,201116831 | 37,095 | 80 |
| A0A509Y3E9 | 2-isopropylmalate synthase | 0,190454699 | 63,645 | 6 |
| D3LRW4 | ABC transporter, ATP-binding protein | 0,187777224 | 24,349 | 33 |
| A0A031H094 | Methionine--tRNA ligase | 0,180527669 | 60,852 | 4 |
| D3LR24 | 50S ribosomal protein L23 | 0,154720684 | 11,239 | 18 |
| A0A4Y8ZJJ8 | 30S ribosomal protein S5 (Fragment) | 0,150988495 | 22,223 | 9 |
| A0A0C2RQ40 | Aldehyde dehydrogenase | 0,145238019 | 55,472 | 11 |
| A0A4Y8PM29 | ABC transporter family substrate-binding protein | 0,122471296 | 66,997 | 531 |
| A0A562FNW9 | Cell division protein FtsW | 0,108869991 | 45,613 | 25 |
| C5C6P1 | Multisubunit Na+/H+ antiporter, MnhC subunit | 0,105896089 | 20,034 | 23 |
| C5CB56 | Potassium uptake protein, TrkH family | 0,071246656 | 45,283 | 17 |
| A0A1R4IG71 | ATP synthase subunit c | 0,037137199 | 7,2405 | 40 |
| A0A378NJU3 | Probable amino-acid-binding protein yxeM | 0,020828509 | 31,171 | 10 |
