# Supplementary material for: Epinephrine extensively changes the biofilm matrix composition in Micrococcus luteus C01 isolated from human skin
Source: Front Microbiol. 2022 Sep 20;13:1003942. doi: 10.3389/fmicb.2022.1003942 (PMC9530943; doi:10.3389/fmicb.2022.1003942)
Supplement: Supplementary file 4 [file Table_3.docx]

Supplementary table S3. Proteins with changed expression in control samples after 72 h of incubation in comparison with 24 h samples. Light green—increase in concentration; dark pink—decrease in concentration.

| Accession | Protein name | Fold change | Mol. weight [kDa] | MS/MS count |
| --- | --- | --- | --- | --- |
| A0A653IWD5 | Uncharacterized protein^[[1]](#footnote-1)^ | 116,1419238 | 12,146 | 18 |
| D3LSF8 | Uncharacterized protein^[[2]](#footnote-2)^ | 27,64114061 | 20,15 | 35 |
| A0A5F0IBK0 | Glycine dehydrogenase (decarboxylating) | 24,40704263 | 104,08 | 179 |
| A0A509Y4T3 | BCCT family transporter | 12,82402495 | 66 | 21 |
| A0A5E8QCZ2 | LysM peptidoglycan-binding domain-containing protein | 10,78697994 | 71,408 | 33 |
| A0A378NNU4 | Acyl-CoA dehydrogenase, short-chain specific | 10,61985879 | 42,21 | 90 |
| A0A031H5M9 | Methionine aminopeptidase | 8,867470926 | 29,093 | 41 |
| A0A509Y3L1 | MBL fold metallo-hydrolase | 7,341070971 | 24,133 | 20 |
| A0A653IQM1 | Thioredoxin | 7,065090089 | 11,473 | 44 |
| D3LR86 | 10 kDa chaperonin | 6,92453661 | 10,346 | 13 |
| A0A2I1XT33 | Oxaloacetate decarboxylase | 6,501522894 | 56,192 | 58 |
| D3LMG6 | Uncharacterized protein^[[3]](#footnote-3)^ | 5,750687375 | 6,053 | 94 |
| A0A6N4F6Z9 | Uncharacterized protein | 5,5087922 | 7,7429 | 15 |
| D3LLD7 | DUF305 domain-containing protein | 5,437588953 | 23,776 | 7 |
| A0A031HXD9 | Uncharacterized protein^[[4]](#footnote-4)^ | 5,302486273 | 35,089 | 13 |
| A0A132HR96 | Chaperone protein DnaK | 5,134185409 | 66,474 | 28 |
| D3LQT0 | Imidazoleglycerol-phosphate dehydratase | 5,06926481 | 22,498 | 21 |
| C5C9L0 | ABC-type spermidine/putrescine transport system, ATPase component | 4,890684909 | 40,392 | 52 |
| A0A6N4C0D6 | Sodium:solute symporter OX=1270 | 4,658845897 | 52,012 | 12 |
| A0A031H0A6 | Demethylmenaquinone methyltransferase | 4,603068872 | 26,868 | 16 |
| C5C835 | Peptidyl-prolyl cis-trans isomerase | 4,528861299 | 19,094 | 24 |
| A0A031HS73 | Alanine aminopeptidase | 4,501025548 | 96,24 | 78 |
| A0A031HM56 | Glutamate dehydrogenase | 4,29633408 | 48,149 | 22 |
| D3LR61 | Glutamine--fructose-6-phosphate aminotransferase [isomerizing] | 4,254221534 | 67,659 | 11 |
| A0A1M7DLB9 | 2,4-dienoyl-CoA reductase | 4,095803853 | 40,054 | 17 |
| A0A132H872 | Phosphoesterase | 4,038649044 | 19,385 | 12 |
| A0A4V6WRJ9 | Protein-tyrosine-phosphatase | 4,029980073 | 27,189 | 24 |
| D3LLW5 | Uncharacterized protein^[[5]](#footnote-5)^ | 4,029397595 | 15,013 | 22 |
| C5CBK5 | Uncharacterized protein^[[6]](#footnote-6)^ | 3,939071713 | 10,878 | 42 |
| A0A1M7ASZ6 | Phosphate import ATP-binding protein PstB | 3,887864711 | 28,468 | 234 |
| D3LNP1 | ATP-dependent Clp protease proteolytic | 3,850679005 | 24,332 | 8 |
| A0A1M7AMP7 | Uncharacterized protein^[[7]](#footnote-7)^ | 3,657824018 | 26,253 | 11 |
| A0A5F0I5R8 | Phosphate-binding protein PstS | 3,545236712 | 38,326 | 280 |
| A0A031HRC0 | Trigger factor | 3,469758875 | 52,178 | 42 |
| A0A4Y8ZP62 | 50S ribosomal protein L7/L12 (Fragment) | 3,375832449 | 8,6456 | 10 |
| A0A509Y330 | Citrate synthase | 3,369727994 | 48,148 | 54 |
| C5C846 | Universal stress protein UspA-like protein | 3,289177865 | 12,133 | 82 |
| A0A4Y8PLP1 | Antigen 84 | 3,239853489 | 21,279 | 78 |
| A0A2N6RPI7 | ATP synthase subunit beta | 2,956188991 | 52,473 | 1201 |
| A0A031HQ70 | Redoxin family protein | 2,94061002 | 21,479 | 32 |
| A0A1M7BQ59 | Trk system potassium uptake protein TrkA | 2,888972279 | 24,21 | 51 |
| A0A653PUG0 | Chlorite O(2)-lyase | 2,836195758 | 27,175 | 115 |
| A0A031H4R3 | Chaperone protein DnaJ | 2,781807752 | 40,228 | 41 |
| A0A5E8QF41 | Glutaminase | 2,660075575 | 65,549 | 71 |
| A0A031GVR3 | ABC transporter, permease protein | 2,475200415 | 24,044 | 62 |
| A0A5F0I6C5 | M13 family peptidase | 2,410886403 | 75,904 | 46 |
| A0A5E8QG41 | Peptidase OX=1270 GN=CYJ94_01980 PE=3 | 2,310479341 | 101,19 | 374 |
| A0A031IAZ7 | Cytochrome aa3 subunit 2 | 2,235278252 | 32,348 | 137 |
| D3LPC0 | DUF3566 domain-containing protein | 2,160971954 | 17,063 | 44 |
| A0A031I8P0 | SURF1-like protein | 2,123477703 | 33,562 | 29 |
| A0A5F0IA93 | ABC transporter permease | 2,122588881 | 45,059 | 47 |
| A0A5F0I8F8 | ATP synthase subunit alpha | 1,998590286 | 59,244 | 1227 |
| C5CBL5 | Multisubunit Na+/H+ antiporter, MnhC subunit | 1,960907358 | 15,009 | 29 |
| A0A562FVN7 | 60 kDa chaperonin | 1,939695588 | 56,721 | 45 |
| A0A031G1Y7 | Uncharacterized protein^[[8]](#footnote-8)^ | 1,846824327 | 14,661 | 66 |
| D3LQK8 | ATP synthase subunit delta | 1,800433317 | 28,627 | 332 |
| A0A509Y3R5 | NAD-dependent succinate-semialdehyde dehydrogenase | 1,776730548 | 49,102 | 152 |
| A0A031IFF2 | Foldase YidC | 1,642343724 | 36,847 | 119 |
| A0A031I9G7 | Glycerol-3-phosphate dehydrogenase | 1,505753791 | 63,978 | 447 |
| A0A5F0I8U8 | ABC transporter ATP-binding protein | 0,604125676 | 27,061 | 167 |
| A0A5E8QD60 | Na+/H+ antiporter subunit E | 0,597738148 | 22,278 | 62 |
| A0A031ISH3 | ABC transporter, ATP-binding protein | 0,582104798 | 56,721 | 234 |
| A0A4Y9HBN9 | Thiol reductant ABC exporter subunit CydC | 0,541158945 | 120,44 | 125 |
| A0A031H3G9 | Sodium/proline symporter | 0,535595294 | 57,523 | 79 |
| A0A1M7DTY7 | Calcium-transporting ATPase CtpE | 0,526028526 | 88,245 | 84 |
| A0A4Y8PJW7 | Iron-sulfur cluster-binding protein | 0,517072011 | 59,017 | 148 |
| A0A2N6RI54 | Uncharacterized protein^[[9]](#footnote-9)^ | 0,509423299 | 9,484 | 35 |
| A0A6N4FFH9 | TerC/Alx family metal homeostasis membrane protein | 0,493834717 | 46,387 | 331 |
| A0A4Y8ZII3 | MFS transporter (Fragment) | 0,49139657 | 51,295 | 35 |
| A0A653IQ80 | (Fe-S)-binding protein | 0,464172187 | 30,726 | 59 |
| C5CCW0 | Putative hydrolase, NUDIX family domain protein | 0,459886384 | 32,46 | 13 |
| A0A1M7A3T0 | DUF2505 domain-containing protein | 0,443907697 | 17,981 | 39 |
| A0A4Y8PKD2 | Acyl-CoA thioesterase II | 0,441403339 | 37,095 | 80 |
| C5CCE9 | Uncharacterized protein^[[10]](#footnote-10)^ | 0,425512549 | 11,222 | 8 |
| C5CAS8 | Uncharacterized protein | 0,419284916 | 17,707 | 9 |
| A0A132HYV3 | S-(Hydroxymethyl)mycothiol dehydrogenase | 0,416147223 | 39,638 | 312 |
| A0A031GV11 | Cation/acetate symporter ActP | 0,40823142 | 56,011 | 128 |
| A0A031GIZ8 | Thioesterase family protein | 0,390430142 | 22,22 | 147 |
| A0A031GMJ1 | Uncharacterized protein^[[11]](#footnote-11)^ | 0,383726101 | 77,766 | 111 |
| A0A653Q1J6 | Putative transporter subunit: ATP-binding component of ABC superfamily transporter | 0,379616028 | 36,14 | 102 |
| A0A653NMS3 | Putative short-chain fatty acid transporter | 0,367177008 | 48,95 | 81 |
| A0A2N6RPM3 | Translation initiation factor IF-2 | 0,340594175 | 97,264 | 47 |
| D3LR25 | 50S ribosomal protein L2 | 0,332136873 | 30,59 | 62 |
| D3LRW4 | ABC transporter, ATP-binding protein | 0,313549901 | 24,349 | 33 |
| A0A5F0I6F9 | ABC transporter ATP-binding protein | 0,307696025 | 23,863 | 22 |
| A0A4U1LCZ2 | Agmatinase | 0,306798317 | 35,919 | 36 |
| P33102 | 50S ribosomal protein L18 | 0,306130464 | 12,961 | 27 |
| A0A031GJQ5 | ABC-type transport system involved in multi-copper enzyme maturation, permease component | 0,292766806 | 44,188 | 35 |
| A0A5F0IAS9 | Uncharacterized protein | 0,266103597 | 42,886 | 22 |
| A0A653NTK0 | Putative membrane protein | 0,24246494 | 14,155 | 32 |
| C5C9H6 | bPH_2 domain-containing protein | 0,239100961 | 19,065 | 20 |
| D3LR36 | 50S ribosomal protein L6 | 0,238726665 | 19,066 | 8 |
| D3LLR8 | Transcriptional regulator MraZ | 0,236395424 | 16,274 | 37 |
| A0A4Y8PM29 | ABC transporter family | 0,223049409 | 66,997 | 531 |
| A0A1M7AJU5 | Methylmalonate-semialdehyde dehydrogenase [acylating] | 0,220663644 | 53,7 | 140 |
| D3LQM4 | Thiazole synthase | 0,214940386 | 28,252 | 9 |
| A0A2N6RPG8 | Acetyl-CoA C-acyltransferase | 0,201467936 | 41,282 | 28 |
| A0A031GHC3 | Isocitrase | 0,194499673 | 47,904 | 5 |
| A0A4Y8ZJJ8 | 30S ribosomal protein S5 (Fragment) | 0,164841364 | 22,223 | 9 |
| D3LR24 | 50S ribosomal protein L23 | 0,156741653 | 11,239 | 18 |
| A0A1R4I9U9 | Urocanate hydratase | 0,139925782 | 61,539 | 11 |
| A0A562G3K7 | TVP38/TMEM64 family membrane protein | 0,132358729 | 27,262 | 17 |
| A0A2I1XXJ8 | Acetyl-/propionyl-CoA carboxylase subunit alpha | 0,116461145 | 65,464 | 64,16 |
| A0A562FTW1 | Regulator of protease activity HflC (Stomatin/prohibitin superfamily) | 0,107276897 | 42,321 | 20,498 |
| A0A378NJU3 | Probable amino-acid-binding protein yxeM | 0,080896601 | 31,171 | 8,3932 |

1. A membrane protein [↑](#footnote-ref-1)
2. A copper chaperone [↑](#footnote-ref-2)
3. A DUF3117-containing protein [↑](#footnote-ref-3)
4. A tripartite tricarboxylate transporter substrate binding protein [↑](#footnote-ref-4)
5. A putative tic20 protein (chloroplasts?) Membrane [↑](#footnote-ref-5)
6. A transmembrane protein [↑](#footnote-ref-6)
7. Twin arginin translocation pathway signal sequence [↑](#footnote-ref-7)
8. A transmembrane protein [↑](#footnote-ref-8)
9. A transmembrane protein [↑](#footnote-ref-9)
10. A DNA binding protein [↑](#footnote-ref-10)
11. 5'-nucleotidase C-terminal domain-containing protein [↑](#footnote-ref-11)
